# A Chemical Approach to Assess the Impact of Post-translational Modification on MHC Peptide Binding and Effector Cell Engagement

**DOI:** 10.1021/acschembio.4c00312

**Published:** 2024-08-16

**Authors:** Joey J. Kelly, Nathaniel Bloodworth, Qianqian Shao, Jeffrey Shabanowitz, Donald Hunt, Jens Meiler, Marcos M. Pires

**Affiliations:** † Department of Chemistry University of Virginia Charlottesville, Virginia 22904, United States; ‡ Division of Clinical Pharmacology, Department of MedicineVanderbilt University Medical Center, Nashville, Tennessee 37240, United States; § Institute of Drug Discovery, Faculty of MedicineUniversity of Leipzig, Leipzig, SAC 04103, Germany; ∥ Center for Structural Biology Vanderbilt University, Nashville, Tennessee 37232, United States; ⊥ Department of Chemistry Vanderbilt University, Nashville, Tennessee 37232, United States

## Abstract

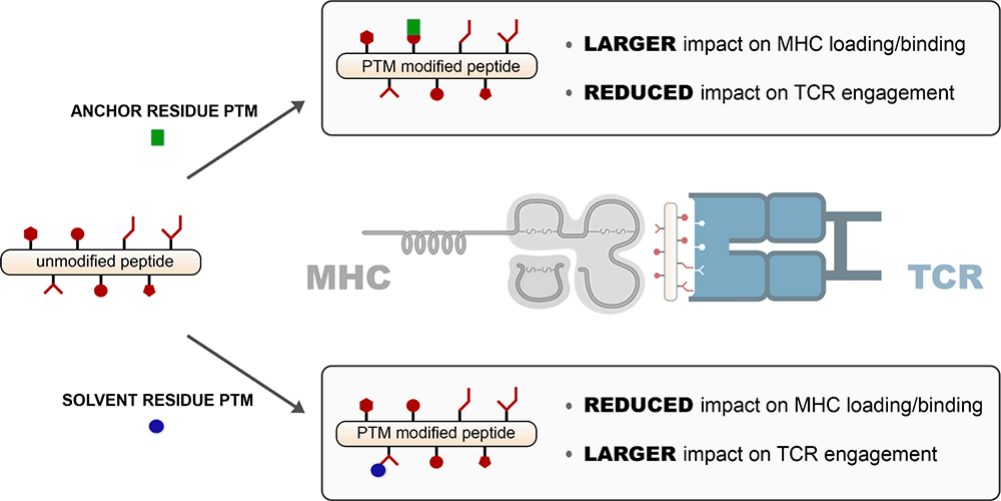

The human major histocompatibility complex (MHC) plays a pivotal role in the presentation of peptidic fragments from proteins, which can originate from self-proteins or from nonhuman antigens, such as those produced by viruses or bacteria. To prevent cytotoxicity against healthy cells, thymocytes expressing T cell receptors (TCRs) that recognize self-peptides are removed from circulation (negative selection), thus leaving T cells that recognize nonself-peptides. Current understanding suggests that post-translationally modified (PTM) proteins and the resulting peptide fragments they generate following proteolysis are largely excluded from negative selection; this feature means that PTMs can generate nonself-peptides that potentially contribute to the development of autoreactive T cells and subsequent autoimmune diseases. Although it is well-established that PTMs are prevalent in peptides present on MHCs, the precise mechanisms by which PTMs influence the antigen presentation machinery remain poorly understood. In the present work, we introduce chemical modifications mimicking PTMs on synthetic peptides. This is the first systematic study isolating the impact of PTMs on MHC binding and also their impact on TCR recognition. Our findings reveal various ways PTMs alter antigen presentation, which could have implications for tumor neoantigen presentation.

## Introduction

**Figure 1:**
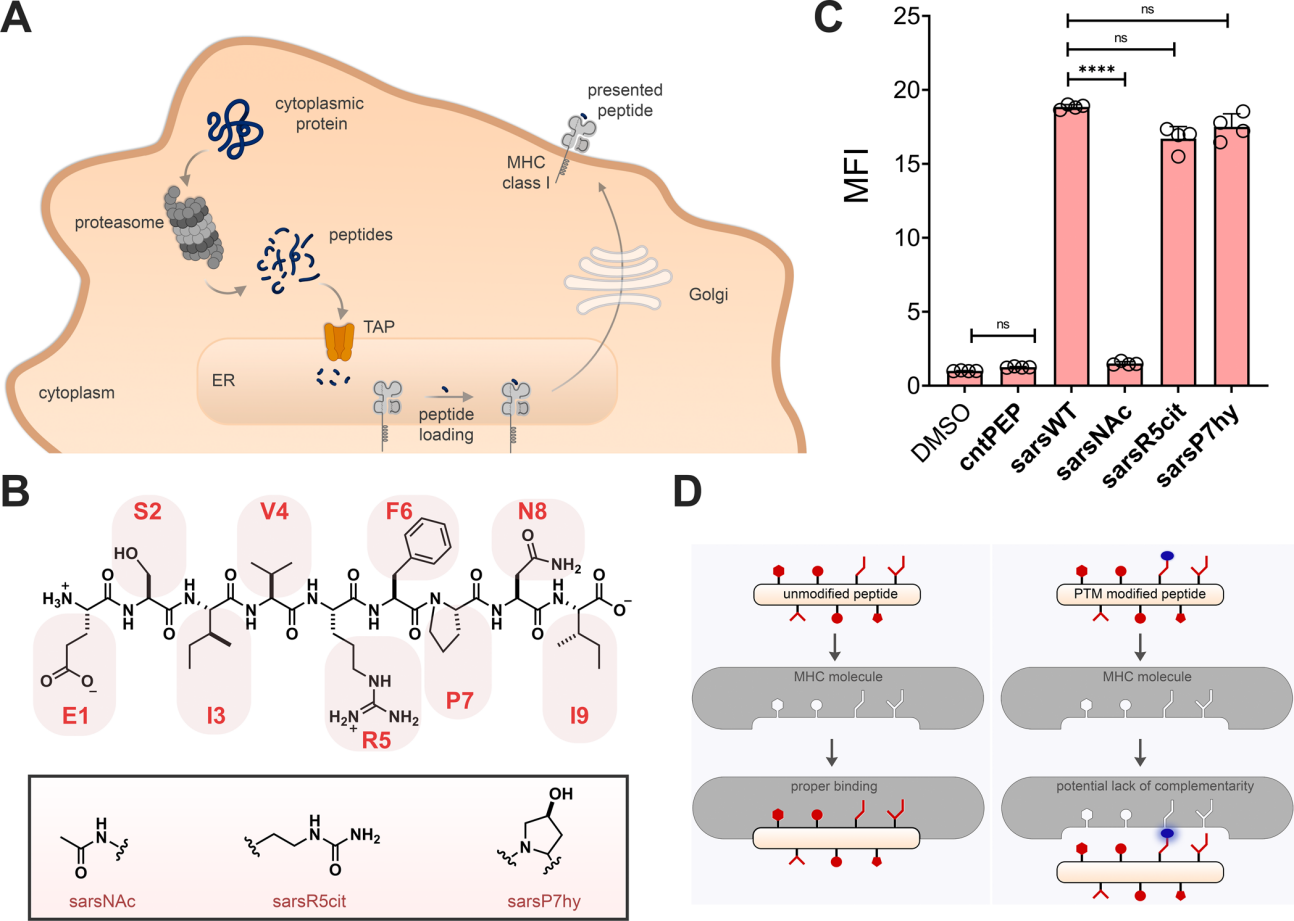
(A) Schematic representation of the process involving the proteolytic processing of cytosolic protein into peptides. Subsequent steps lead to the loading of the peptides onto MHC molecules that are then transported to cell surface for presentation. (B). Chemical structures of **sarsWT** and the PTM modified variants. (C) Flow cytometry analysis of RMA-S cells treated with specific peptide (20 μM) detected by APC conjugated antimouse H-2K^b^ antibody. Data are represented as mean ± SD (*n* = 3). P-values were determined by a two-tailed *t*-test (**** *p* < 0.0001, ns = not significant). (D) A general schematic representation of how the orientation of the PTM within the presented peptide could negatively impact binding to MHC molecules (*right*) compared to the unmodified peptide (*left*). Note that modifications can also occur on the termini of the peptides.

To maintain homeostasis, the human immune system must efficiently recognize and destroy cells that have accumulated genetic mutations or have been invaded by pathogenic microorganisms.^1,2^ Self-identification to the immune system is a primary mechanism deployed by human cells to flag the presence of nonself-proteins or proteins produced by genetic lesions.^3^ To this end, presentation of antigenic peptidic fragments via the major histocompatibility complex (MHC) to surveying immune cells serves as a key system to recognize diseased cells (Figure 1A).^2^ MHC is present in the membrane of every nucleated cell and is responsible for presenting both antigenic and endogenous protein fragments to the extracellular space. The recognition of peptide-MHC complexes (pMHCs) by T cell receptors (TCRs)^4^ initiates an immune cell response, the nature of which depends on the type of the T cell (primarily CD4+ and CD8+ cells).^5^

Critically, precise recognition and binding of pMHCs by TCRs must operate with a high level of fidelity since the response to self-peptides in healthy cells can result in cellular injury.^6^ Through negative selection, thymocytes expressing TCRs that bind tightly to self-peptides are removed from circulation.^4,7^ In theory, changes to the primary sequence of a protein could yield autoreactive pMHC as long as the primary sequence was not part of the negative selection process. Some primary sequence changes can be permanent such as amino acid changes due to gene mutations, while others can be transient, like post-translational modifications (PTMs). In healthy states, enzymatic PTMs are added by specific sets of enzymes to modulate protein activity, localization, and interactions of the protein with other cellular components.^8^ Because PTMs are covalent modifications, most are stable enough to persist through protease digestion and loading onto MHC molecules, potentially leading to the presentation of modified peptide during immune surveillance.^9^

Remarkably, human proteins containing PTMs have been shown to be excluded from negative selection, despite the potential that pMHCs with PTM-modified peptides to yield autoreactive cells.^10^ It has been hypothesized that this exclusion is due to the relatively low abundance of PTMs in healthy and young adults in the prime phase of negative selection, compared to the unmodified parent proteins. However, thymus involution after adolescence may contribute to age-related autoimmune diseases.^11,12^ Additionally, various factors, such as imbalances of PTM writers and erasers in diseased states, cellular stresses, or aging, can dramatically increase the prevalence of PTMs across the proteome.^13^ Collectively, the increased level of pMHCs bearing PTMs mean that a higher proportion of potentially autoreactive cells is present.^14−20^ In other words, when peptides with PTM modifications are presented, the immune system may perceive them as nonself, triggering a pathogenic autoimmune response.^21−24^ A recent example of this phenomenon involved cysteine carboxyethylation by a cellular metabolite, leading to a pathogenic neoantigen presented on pMHC and resulting in autoreactive T cell responses.^25^

Various PTMs have been previously found in pMHCs.^26−34^ Notably, increased conversion of arginine to citrulline in the myelin sheath has been shown to lead to the development of self-reactive T cells that exacerbate the progression of autoimmune diseases such as Rheumatoid Arthritis (RA) and Multiple Sclerosis (MS).^35−40^ Moreover, there is mounting evidence that pMHCs presenting peptides with PTMs are prevalent in tumors.^41−44^ A recent report revealed that PTMs can shape the antigenic landscape.^44^ Despite the importance of PTMs in potentially generating autoreactive peptides, several aspects of PTM biology in this field remain poorly defined. One primary barrier to better understanding the impact of PTMs on pMHCs has been the complexity of assigning the role of PTMs in the context of the many steps leading to MHC presentation. These processes may include changes in a protein half-life, altered proteolytic processing to generate presentable peptides, or binding affinity of individual peptides to MHC molecules. Among these factors, peptide binding affinity to MHC molecules is a primary determinant of the peptide repertoire within pMHCs.^45,46^ Here, we systematically isolate the impact of PTMs on the ability of the peptides to bind to MHC molecules and be recognized by cognate T cells.

## Results and Discussion

In general, PTMs can be challenging to detect in the context of complex biological systems, and the levels of PTMs can change in response to cellular cues.^47^ Traditional cellular assays do not typically establish the ratio of PTM proteins relative to their unmodified counterparts.^48^ These challenges are amplified when analyzing PTMs on peptides from pMHCs.^49^ Extracting peptides from isolated MHCs involves challenging separation steps and downstream mass spectrometry analysis.^50^ On the other hand, a top-down approach of treating cells with PTM-modified proteins also poses a challenge because exogenous proteins are not readily processed to yield peptides that are presented by MHC I molecules.^51^ Even if the peptides get processed, the ultimate pMHC presentation involves multiple factors including peptide half-life,^52^ uptake efficiency, or peptide binding affinity to MHC. Consequently, assessment of how various naturally occurring PTMs impact peptide binding to MHCs and recognition by TCRs has not been systematically conducted.

To address these challenges, we based our approach on the RMA-S cell line, which lacks the TAP importer.^53,54^ TAP is essential for delivering peptides from the cytosol to the endoplasmic reticulum. By incubating peptides with RMA-S cells at lower temperatures, we allowed peptides to associate with surface-bound MHCs (Figure S1). Upon increasing the temperature, we observed the stabilization of pMHCs on the cell surface. The quantity of MHC on the cell surface correlated with the binding affinity^55−60^ of the peptide to the MHC molecule, quantified via a fluorescently labeled anti-MHC antibody.^61^ Given the features of this well-established cell line, we anticipated that RMA-S cells would effectively reveal the impact of PTMs on peptides that bind to MHC with high affinity. Moreover, RMA-S cells have been used extensively for isolating the affinity of a peptide for MHC.

To start, we sought to identify a peptide from SARS-CoV-2 spike protein that could be displayed on MHC molecules. We used NetMHCPan4.0,^62^ an algorithm that estimates the propensity of peptides to bind MHC, to select a peptide sequence (ESIVRFPNI, referred to as **sarsWT**) for stabilization of the specific MHC molecule (H-2K^b^) on the surface of RMA-S cells. The peptide was synthesized using standard solid-phase peptide synthesis techniques. The culture medium of the RMA-S cells was supplemented with **sarsWT** at 26 °C to enable association of the peptide with surface bound MHCs. Following **sarsWT** incubation, the temperature was raised to 37 °C. Subsequently, the cells were incubated with APC conjugated antimouse H-2K^b^ antibody and analyzed by flow cytometry where fluorescence is expected to correspond with the amount of **sarsWT** bound to the MHC. The peptide SNFVSAGI (**cntPEP**) was used as a negative control since it has been reported to not interact appreciably with H-2K^b^.^63^ Various concentrations of **sarsWT** were used to optimize the RMA-S stabilization assay with the goal of increasing the number of pMHC molecules formed on the surface of the cells (Figure S2). We found an increased fluorescence signal corresponding to increasing concentrations of **sarsWT,** indicating that the peptide bound and stabilized the MHC on the surface of the cell. The amount of stable pMHCs that formed at the surface also saturated at 50 μM of peptide and showed an EC_50_ of 1.7 μM. These results confirmed the suitability of RMA-S to report on the stabilization of the pMHC complex by **sarsWT**.

Another parameter to consider is the time in which the MHC binding peptide has to associate with the MHC to reach MHC saturation. To evaluate this time frame, the workflow described above was conducted; however, the peptide incubation time at 37 °C was varied from 2 to 6 h (Figure S3). The results showed that the fluorescent signal increased with increased incubation periods. This is likely due to accumulation of stabilized pMHCs in the presence of excess high-affinity peptide. Nevertheless, a 6 h incubation at 37 °C provided the highest signal-to-noise ratio and was used for downstream assays.

With an optimized protocol in hand, we then synthesized a panel of peptides containing PTMs to investigate their potential impact on MHC binding (Figure 1B). We envisioned that modifying residues on **sarsWT** would be representative of how the selected modifications may impact binding of different MHC peptides. The specific PTMs chosen were a proline to hydroxyproline,^64,65^ arginine to citrulline,^66^ and *N*-terminus acetylation^67^ modifications, as they are naturally occurring PTMs. By performing the RMA-S stabilization assay, we found that the citrullination and hydroxy-proline modifications had no significant effect on MHC binding, but the *N*-terminal acetylation modification completely disrupted MHC binding (Figure 1C). Gratifyingly, there is precedence for *N*-acetylated peptides to be displaced in MHC class I.^68,69^ These results demonstrate that naturally occurring PTMs can alter pMHC complex formation and influence the antigen presentation pathway depending on the interface of the peptide and the MHC molecule (Figure 1D).

**Figure 2:**
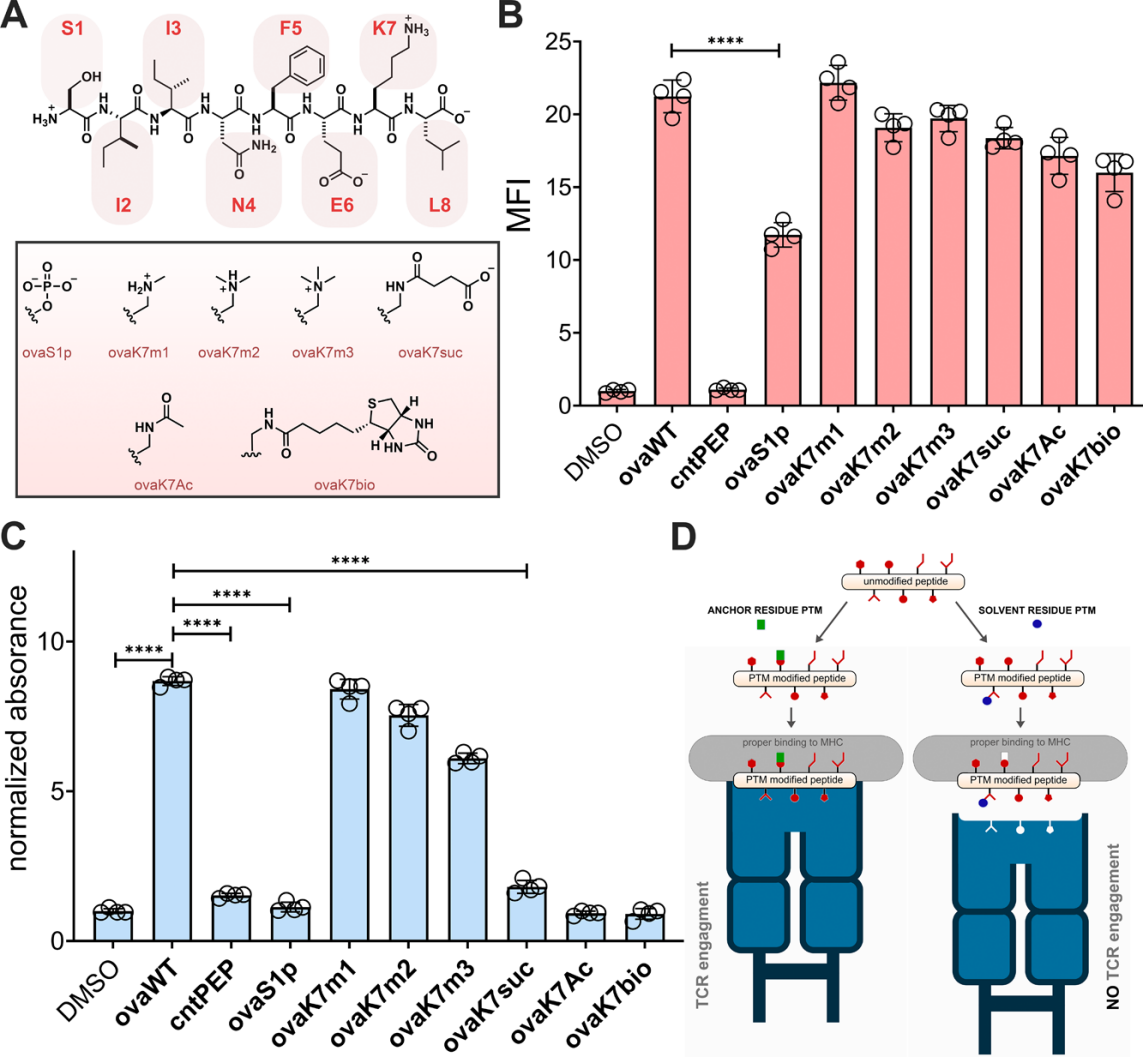
(A) Chemical structures of **ovaWT** and the PTM modified variants. (B) Flow cytometry analysis of RMA-S cells treated with specific peptide (20 μM) detected by APC conjugated antimouse H-2K^b^ antibody. (C) RMA-S cells were incubated with peptide and B3Z T cells overnight at an effector to target ratio of 1:1. β-galactosidase expression was then measured via the colorimetric reagent CPRG on a plate reader at 570 nm. (D) Schematic representation of how PMTs can impact engagement with TCRs. Data are represented as mean ± SD (*n* = 4). P-values were determined by a two-tailed *t* test (**** *p* < 0.0001)

Next, we shifted our focus to the peptide epitope SIINFEKL (**ovaWT**) from the model antigen ovalbumin (OVA) to interrogate how other PTMs impact MHC binding as well as TCR recognition.^70^ To this end, we synthesized a new library of OVA peptides containing PTMs on its serine and lysine residues and performed a concentration scan to assess MHC binding using the RMA-S stabilization assay (Figure 2A). Interestingly, there was a significant decrease in MHC binding when serine was phosphorylated despite predictions that this site does not contribute significantly to binding (Figure 2B).^71^ Our results highlight the potential deficiency of current models to predict how PTMs could impact peptide binding to MHC molecules given how PTM products can be structurally distinct relative to the side chains of the canonical amino acids.

The side chain of lysine (K7) on **ovaWT** was explored next. Lysine was decorated with different types of PTMs that included three different charge states. Previously, the same lysine on SIINFEKL had been altered with various groups including fluorophores and caging groups.^72−74^ Our data showed considerable accommodation of structural alterations to the lysine side chain (Figure 2B). With larger modifications such as biotin, binding levels are altered but the change is not significant. These findings can be explained by the solvent exposed nature of lysine on the pMHC complex and the length of the lysine side chain, which may reduce structurally unfavorable interactions with the MHC molecule (Figure 1D).^75^ Critically, for **ovaWT** the anchor residues that are more important for the overall binding affinity to MHC molecules (Phe5 and Leu8) are not amendable to conventional PTMs.^76^

We then sought to detect changes in peptide binding via a competition assay using the parent cell line (RMA) that has a competent TAP system. To this end, we performed a competition assay using the panel of OVA peptides barring PTM mark in live RMA cells. The assay was performed by coincubating RMA cells individually with PTM-modified peptides and also **ovaFl** (SIINFEK(FITC)L), in which the γ position of lysine is modified with fluoresceine. **ovaFl** has long been utilized by the field to interrogate binding of untagged peptides for MHC molecules. A decrease in cellular fluorescence should indicate higher association levels with MHC molecules, thereby informing on the relative affinity of the PTM-modified molecules. Critically, this assay retains MHC molecules within a physiologically relevant context and this alternative readout should complement the RMA-S MHC stabilization assay. As expected, the trend in binding affinity in the competition experiment closely mirrored our results with the RMA-S stabilization assay indicating that the RMA-S assay can accurately report on relative MHC binding affinities in a cellular context (Figure S4). As further evidence and to specifically detect PTM-modified peptides bound on MHC, **ovaWT** and **ovaK7m3** treated RMA-S cells had their MHC-bound peptides eluted and identified using mass spectrometry. The canonical binding peptide, ovaWT, was clearly detected after elution from live RMA-S cells (Figures S5 and S6). Likewise, the **ovaK7m3** was detected by mass spectrometry and this provides direct evidence that trimethylation of K7 does not disrupt binding of this peptide to MHC (Figures S7 and S8). While PTMs on solvent exposed residues could potentially retain binding to MHCs, their overall impact may be more strongly reflected in the pMHC engagement with TCRs. TCRs recognize T cell exposed motifs of the bound peptide within pMHCs.^77,78^ Therefore, PTMs at these positions may have a greater impact on T cell activation.

To assess the impact of **ovaWT** PTMs on TCR recognition, we utilized the B3Z T cell hybridoma cell line that contains an OVA specific TCR along with a NFAT-LacZ reporter gene that encodes for β-galactosidase on an IL-2 inducible promoter.^79^ Upon B3Z recognition of the OVA pMHC complex on RMA-S cells, IL-2 production promotes β-galactosidase expression, which can hydrolyze chlorophenol red-β-D-galactopyranoside (CPRG), and the resulting color change is representative of activation levels. Our results showed that the PTMs have a much more significant effect on TCR recognition than on peptide binding to MHC (Figure 2C). While relatively modest disruption of TCR recognition was seen for increasing the degree of methylation on the lysine residue, near complete disruption of TCR recognition was seen for all PTMs that imparted a change in charge of either the serine or lysine residue. Additionally, the reduction in TCR recognition for both the phosphoserine and biotinylated OVA cannot be fully explained solely by their decrease in MHC binding. Instead, the PTMs that are well accommodated for MHC binding could be displayed away from the binding cleft and alter engagement with TCRs on cognate T cells (Figure 2D).

**Figure 3:**
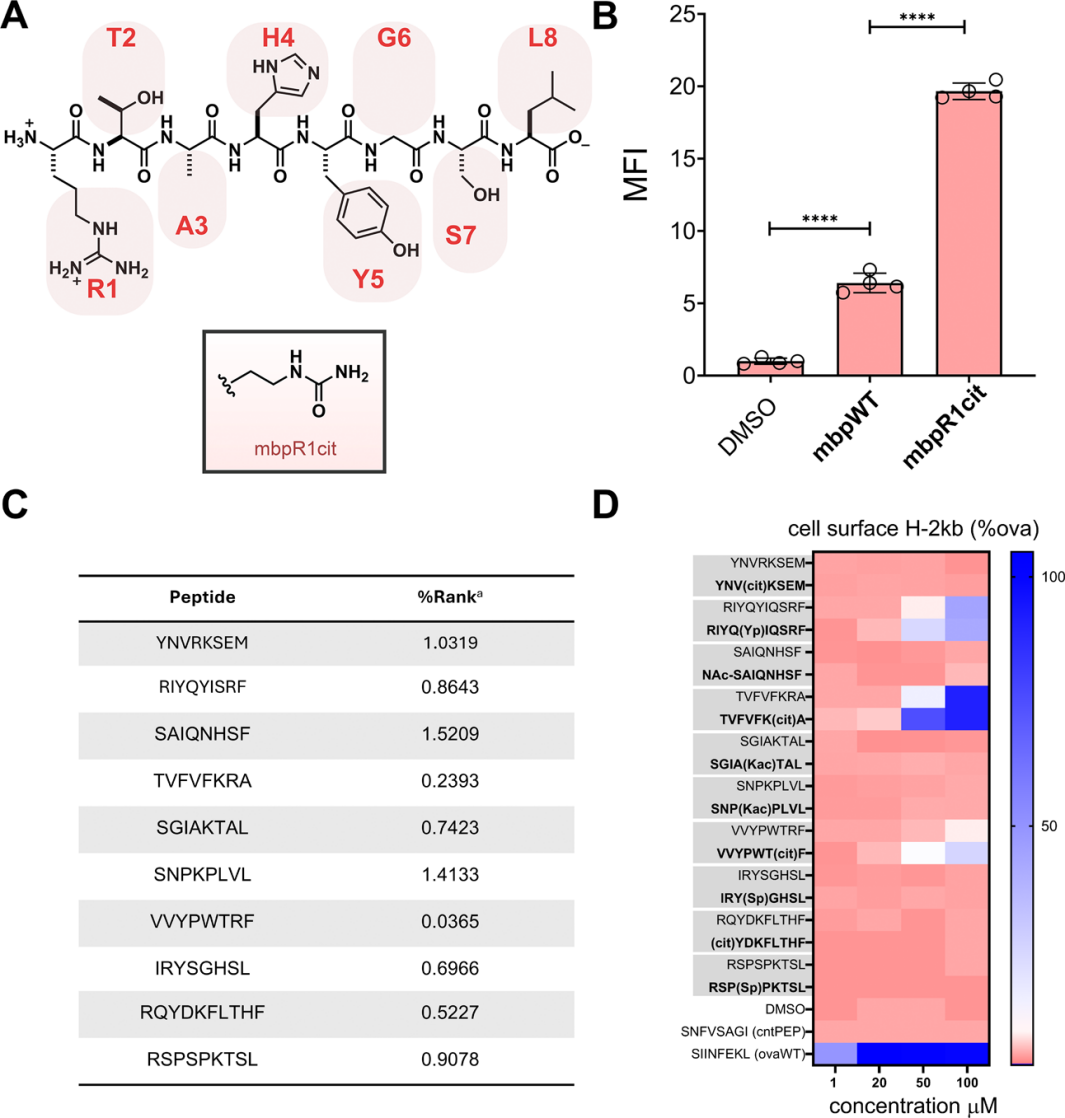
(A) Chemical structures of **mbpWT** and the PTM modified variant. (B) Flow cytometry analysis of RMA-S cells treated with specific peptide (20 μM) detected by APC conjugated antimouse H-2Kb antibody. (C) NetMHCpan 4.1 predicted binding scores of tumor associated peptides. %Rank is the prediction score for comparing MHC binding across random peptides where a %Rank of 1 indicates that a peptide scored in the top 1% of random peptides. A %Rank of <2 or <0.5 are the cutoffs for weak binding or strong binding to MHC respectively. A %Rank >2 indicates nonbinders. (D) A heat map of wild-type and PTM modified peptides relative MHC binding analyzed by using the RMA-S stabilization assay. RMA-S cells were incubated with peptide at indicated concentration of peptides and detected via flow cytometry with APC conjugated antimouse H-2K^b^ antibodies. Abbreviations: Cit (citrullination), Yp (phosphorylated tyrosine), Kac (lysine acetylated at the γ position), NAc (acetylation at the α position), and Sp (phosphorylated serine). Data are represented as mean ± SD (*n* = 4). P-values were determined by a two-tailed *t* test (**** *p* < 0.0001).

To confirm the relevance of these findings in a broader context, we also performed the T cell activation assay on DC2.4 dendritic cells, which also express H-2K^b^. Unlike the RMA-S cells, peptides were incubated with DC2.4 cells at 37 °C and were expected to load onto MHC molecules if they display sufficient affinity toward H-2K^b^. Satisfyingly, the pattern of T cells activation with PTM modified **ovaWT** peptides showed a similar profile with DC2.4 cells as RMA-S cells (Figure S9). Our results confirmed that the impact of PTMs on T cell activation may be consistent across multiple cell types. Finally, we sought to investigate whether we could assess PTMs on a peptide that has potential pathological implications. To this end, a number of autoimmune diseases, including RA and MS, have been described to involve the citrullination of arginine.^80^ Citrullination is carried out by peptidylarginine deiminases (PADs) and higher levels of citrullinated proteins have been found in older mice relative to young mice.^81^ A shift in higher levels of citrullination past the full scope of negative selection could provide a pathway for autoreactivity. Using a peptide originating from a known PAD substrate myelin basic protein (RTAHYGSL, **mbpWT)** as the baseline (Figure 3A), we found that there was a statistically significant increase in peptide binding to MHC molecules upon citrullination (Figure 3B). This result is consistent with the possibility that PTMs can impact presentation of peptides in the context of disease-linked peptides.

We then sought to uncover if PTMs are playing a role in influencing the presentation of cancer antigens. However, only a limited number of studies have identified a wide range of PTM modified MHC peptides presented on cancer cells.^43,44^ Additionally, the specific impact of these PTMs on peptide-MHC binding remains unclear. To this end, we synthesized 10 PTM modified MHC peptides previously identified on cancer cells and compared their binding affinity to MHC to their respective unmodified versions.^43,44^ Out of this library, only 3 PTM modified peptides were found to bind to MHC at the biologically relevant concentrations, highlighting the need for secondary validation assays for peptide libraries identified through mass spectrometry screens (Figure 3C,D). Interestingly, out of the peptides that bound, the PTM versions all showed significant improvement in MHC stabilization on RMA-S cells suggesting that PTMs may enhance the presentation of cancer associated peptides (Figure S10).

**Figure 4:**
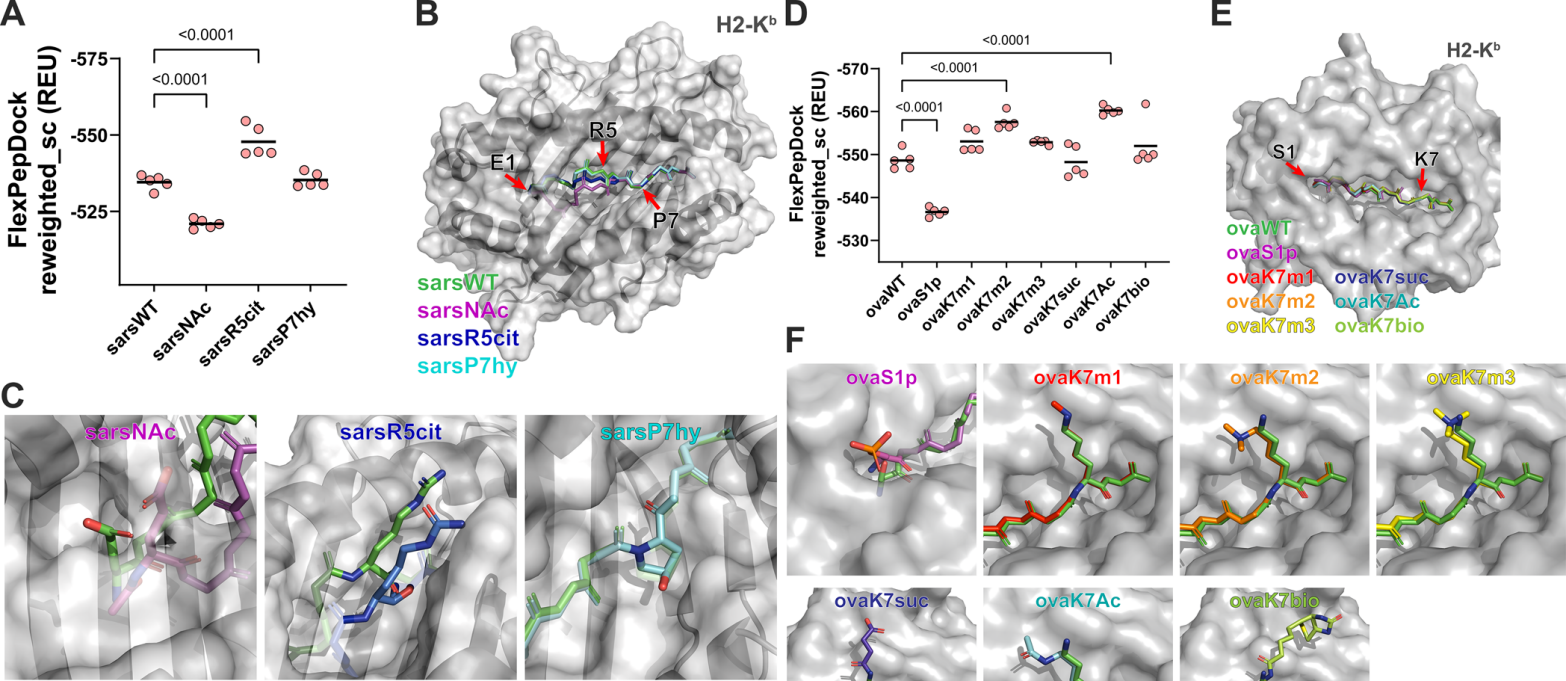
Modeling the sars peptide with and without PTMs using FlexPepDock. (A) N-terminal acetylation results in higher reweighted_sc values (and thus lower likelihood of binding) compared to the wild-type peptide, consistent with experimental results (one-way ANOVA and Holm-Sidak posthoc; individual values correspond to the top 0.5% of models generated, and bars represent mean score term values). (B) Top peptide models generated for each epitope positioned in the H-2Kb binding cleft. (C) Side chain modifications compared to the unmodified epitope for the SARS-CoV-2 peptide. Modeling the ovalbumin-derived peptide with and without PTMs using FlexPepDock. (D) Phosphorylation of the N-terminal serine residue generates models with higher average reweighted_sc terms, similar to N-terminal acetylation of the SARS-CoV-2 peptide (one-way ANOVA and Holm-Sidak posthoc; individual values correspond to the top 0.5% of models generated, and bars represent mean score term values). (E) Superimposed peptide backbones for the top peptide models generated for each PTM. (F) Detailed side-chain configurations for the top-scoring models generated by FlexPepDock.

To gain additional insight into how PTM s might alter peptide binding affinity to H-2K^b^, we simulated peptide binding to H-2K^b^ using ROSETTA and FlexPepDock. FlexPepDock has been previously benchmarked against MHC-I bound peptides and is capable of generating models with subangstrom accuracy.^82,83^ One advantage of FlexPepDock over contemporary machine learning methods is its ability to incorporate noncanonical amino acids and PTM residues by generating custom ROSETTA parameters files; this enables FlexPepDock to generate accurate models of MHC-I bound epitopes containing such residues.^84,85^ Furthermore, the binding energy metrics calculated by this application (the reweighted_sc score term) can serve a surrogate for peptide binding affinity to MHC-I.^86^ We simulated the binding of the SARS-CoV-2-derived peptide to H-2K^b^ and generated results recapitulating experimentally derived binding affinity changes (Figure 4). ROSETTA correctly predicts decreased binding affinity for the top-scoring decoys after N-terminal acetylation (corresponding to an increase in the average reweighted_sc term; ROSETTA energy metrics are inversely proportional to binding affinity). ROSETTA predicts either no change or a modest increase in binding affinity for the other two PTMs assessed (Figure 4A). When the top-scoring model for each peptide is visually inspected, we noted significant alterations in the peptide backbone structure for the acetylated N-terminus peptide variant largely confined to the H-2K^b^ N-terminus binding pocket (Figure 4B), a region that is largely responsible for dictating epitope binding specificity and that cannot easily accommodate large conformational changes.^9^ Conversely, R5 citrullinated and P7 hydroxylated variants are located outside the H-2K^b^ binding pockets and regions tolerant of larger conformational changes (Figure 4C).

We repeated this analysis on the ovalbumin-derived peptide and each PTM (Figure 4D). Phosphorylation of the N-terminus serine generates structures with higher (and therefore less favorable) score terms, again likely due to disruption of the *N*-terminus peptide-binding pocket (Figure 4E). Peptide models with the remaining PTMs scored either equivalently or slightly better than their wild-type counterparts. While largely consistent with experimental data, ROSETTA predicts modest increases in peptide binding favorability that are not observed experimentally. This may be a result of biases inherent in ROSETTA’s scoring weights that have not been fully optimized for unusual or noncanonical amino acids such as those modeled here. Alternatively, the assay used for assessing binding affinity may be insufficiently sensitive to detect small changes above certain affinity thresholds. Modifications at the lysine in position 7 alter T cell activation *in vitro* as illustrated in Figure 2C. *In silico*, these altered residues do not interact appreciably with peptide binding pockets but instead occur in the region displayed by MHC-I to patrolling T cells (Figure 4F). Methylated and acetylated lysine alters residue charge and hydrophobicity; succinylation adds a negative charge (as opposed to lysine’s normally positively charged side chain); and biotinylation results in the addition of a large aliphatic heteropolycyclic side chain very different in size and character from the native lysine residue.

Finally, we performed a similar analysis with the **mbp** peptide with and without R1 citrullination and generated *in silico* results that recapitulated experimental findings (Figure 3). Citrullination at R1 significantly increases the reweighted_sc metrics for the models generated by ROSETTA (Figure S11A). While this modification occurs at the N-terminus binding pocket, it does not appreciably alter peptide backbone configuration (Figure S11A, in contrast to the effects of N-terminal acetylation of the **sars** peptide). It does, however, alter side chain hydrophobicity and charge. Given that the citrullinated variant is charge-neutral (compared to arginine’s 1+ charge) and given H-2Kb’s preference for hydrophobic or charge-neutral residues at binding pockets, it is reasonable to expect this modification may enhance peptide binding affinity.

Increasingly, there are efforts dedicated to developing therapeutic agents that harness a patient’s immune system against cancerous lesions.^87−89^ A prominent example involves the modulation of the programmed death-1 (PD-1) system and its cognate programmed death-ligand 1 (PD-L1). Cancers can often hijack this set of proteins to maintain growth while suppressing the patient immune response against transformed cells. Immune checkpoint inhibitors that disrupt the association of PD-1 and PD-L1 have shown potent anticancer activity by a number of mechanisms but primarily via increased T cell engagement.^90,91^ Neoantigens arising from genetic lesions likely provide a pool of antigens that can be presented by cancer MHCs on the cell surface during checkpoint therapy. Importantly, our results suggest that therapeutic agents that increase the presentation of peptides through an altered balance of PTM marks could potentially complement the targeting antigenic pool.

Considering the observed roles of PTMs in MHC binding and recognition by TCRs, it is conceivable that the balance of PTM addition by “writers” and PTM removal by “erasers” can be central in immunological health. Any imbalance that could be driven by age or manufactured by therapeutic interventions could result in pathological state. For example, proteins could naturally exist in high abundance of nonacetylated states on lysine side chains past the peak period of negative selection of T cells. After cancer development, there is an increased level of acetylation, but those marks need to be removed by “erasers” called Lysine Deacetylases (KDAC) to reduce an anticancer immunological response against the cancerous cells. Upon the administration of KDAC inhibitors, a larger pool of acetylated peptide would be presented. As our data showed, the acetylation of lysine that interacts with TCRs would escape negative selection and provide an anticancer immunological response. Such “cryptic” antigens could potentially be driving the pharmacological effect of some PTM modulators in cancer patients. To this end, there are a high number of ongoing studies evaluating the combination of KDAC inhibitors and PD-1/PDL-1 inhibitors across many types of difficult-to-treat tumors.^92−98^ We are currently working toward using this strategic platform to demonstrate how KDAC inhibitors directly lead to an immunopeptidome that reveal cryptic antigens able to enhance antitumor immunological activity.

## Conclusions

In conclusion, the data presented here demonstrate that naturally occurring PTMs can drastically impact the antigen presentation pathway by altering either their affinity toward MHC molecules or TCR recognition by cognate T cells. In our assays, we showed that some PTMs can disrupt peptide binding to MHC, but primarily on residues that directly engage with MHCs. Conversely, PTMs on residues whose side chains are positioned away from the MHC binding face are more sensitive to TCR recognition due to the altered binding modality with TCRs. To the best of our knowledge, our study is the first systematic analysis of the impact that PTMs have on MHC binding in a whole cell context and how PTMs can also alter T cell activation using live target and effector cells. Provided that negative selection is age-dependent and becomes increasingly diminished past adolescence, it is evident that PTMs could be causal events that result in autoimmune diseases.

## Methods

### Mammalian Cell Culture

RMA-S cells were a kind gift from Dr. John Sampson. RMA-S cells were maintained in RPMI 1640 media supplemented with 10% fetal bovine serum, 50 IU/mL penicillin, 50 μg/mL streptomycin,1X MEM nonessential amino acid solution (ThermoFisher) and cultured in a humidified atmosphere of 5% CO_2_ at 37 °C. B3Z cells were kindly provided by Dr. Aaron Esser-Kahn and maintained in RPMI 1640 media supplemented with 10% fetal bovine serum, 50 IU/mL penicillin, 50 μg/mL streptomycin and cultured in a humidified atmosphere of 5% CO_2_ at 37 °C.

### RMA-S Stabilization Assay

10^5^ RMA-S cells were seeded in a treated 96 well plate at 37 °C overnight. The next day, RMA-S cells were moved to a 26 °C incubator for 24–48 h. Following the incubation period, cells were incubated with peptides in culture media at indicated concentrations for 1 h at 26 °C before being moved to the 37 °C incubator for 6 h. The media was then replaced with a 1:100 dilution of APC-labeled antimouse H-2K^d^/H-2D^d^ antibody in culture media for 1 h at 4 °C. Cells were removed from the well plate by vigorous pipetting, fixed with 2% formaldehyde solution, and analyzed using the Attune NxT Flow Cytometer (Thermo Fischer) equipped with a 637 nm laser with 670/14 nm bandpass filter.

### Mild Acid Elution

10^9^ RMA-S cells were treated with 20 μM peptide at 26 °C for 1 h. Cells were then warmed to 37 °C for 6 h. RMA-S cells were collected by spinning down at 500 x *g* for 5 min and thoroughly washed with 1X PBS Buffer pH 7.4 (Invitrogen). Next, 15 mL of mild acid elution (MAE) buffer (0.131 M citric acid, 0.066 M Na_2_HPO_4_, 150 mM NaCl, 0.3 μM Aprotinin, and 5 mM iodoacetamide at pH 3.3) was added to RMA-S cells for 2 min. RMA-S cells were then spun down at 4000 x *g* for 5 min and the supernatant was collected, lyophilized, and stored at −80 °C until further use.

### Mass Spectrometry Analysis

The emitter tip of the analytical column was laser-pulled to produce an opening of 2–5 μm, and a 2 mm kasil frit was used in place of the irregular reverse phase (RP) resin. The precolumns (100 μm i.d. x 360 μm O.D. fused silica) were packed to 7 cm with 10 μm C18 beads, and the analytical columns (75 μm i.d. x 360 μm o.d. fused silica) were packed to 10 cm with 3 μm C18 beads. Ova samples and 100 fmol each of the internal standards (Angio and Vaso) were loaded onto the precolumn using a pressure vessel for a 15 min desalting rinse at a flow rate of 100 nL/min with 0.1% acetic acid (AcOH) in water before connecting to the analytical column with a 2 cm Teflon tubing. Reverse phase separation was conducted at a flow rate of 100 nL/min by HPLC using 0.1% AcOH for solvent A and 0.1% AcOH in 60% acetonitrile for solvent B, and a gradient as follows: 0% to 60% solvent B in 60 min, 60% to 100% solvent B in 2 min, 100% solvent B for 4 min, 0% in 2 min, followed by a 22 min equilibration with 100% solvent A. The peptides eluted from the analytical column were electrosprayed into an LTQ-Orbitrap mass spectrometer. MS1 spectra were acquired with a resolution of 60,000, an AGC target of 5e5 and scan range of 300 to 2000 *m*/*z* in the Orbitrap analyzer, followed by low resolution data dependent MS2 acquisition in the ion trap with normal scan rate. Only precursor ions with charge +2 and +3 were selected for fragmentation. The top 2 most abundant precursor ions, as well as the targeted +1 and +2 precursor masses for the ovaWT and ovaK7m3 peptides, were selected for collision-activated dissociation (CAD) in the ion trap analyzer with an AGC target of 1e4, a normalized collision energy of 35%, an activation time of 30 ms, and a 2.0 *m*/*z* isolation window. If the same precursor ion was selected three times or was detected twice within a 20-s repeat duration, the ion was dynamically excluded for 15 s. The presence of ovaWT and ovaK7m3 peptides was verified by manually inspecting the targeted MS2 spectra for the expected fragmentations and fragment ion masses.

### B3Z T Cell Activation

10^5^ RMA-S cells were seeded in a treated 96 well plate at 37 °C overnight. The following day the culture media was replaced with media containing indicated concentration of peptide along with 10^5^ B3Z cells in culture media and coincubated overnight. Cells were then spun down at 500xg for 5 min and washed with 1X PBS a total of two times. Lysis buffer containing 0.2% saponin, 500 μM CPRG reagent, 100 mM MgCl_2_, and 100 mM β-mercaptoethanol in 1X PBS was added to each well. After 2–4 h absorbance 570 was recorded using a BioTek Epoch 2 microplate reader.

## Supporting Information Available

Additional figures including RMA-S (workflow scheme, concentration scan with sarsWT, and time scan), DC2.4 T Cell activation, modeling of citrullination, and mass spectrometry results from MHC peptides; materials/methods; and synthetic peptide characterization (mass spectrometry and analytical HPLC traces) (PDF)
