# Supplementary material for: A Chemical Approach to Assess the Impact of Post-translational Modification on MHC Peptide Binding and Effector Cell Engagement
Source: ACS Chem Biol. 2024 Aug 16;19(9):1991–2001. doi: 10.1021/acschembio.4c00312 (PMC11420952; doi:10.1021/acschembio.4c00312)

Supporting Information

A Chemical Approach to Assess Impact of Post-Translational Modification on MHC Peptide Binding and Effector Cell Engagement

Joey J. Kelly[1], Nathaniel Bloodworth[2], Qianqian Shao[1], Jeffrey Shabanowitz[1], Donald Hunt[1], Jens Meiler[2,3,4,5], and Marcos M. Pires[1*]

[1]Department of Chemistry
University of Virginia
Charlottesville, VA, United States 22904

[2]Division of Clinical Pharmacology, Department of Medicine
Vanderbilt University Medical Center, Nashville, TN, United States 37240

[3]Institute of Drug Discovery, Faculty of Medicine
University of Leipzig, Leipzig, Germany SAC 04103

[4]Center for Structural Biology
Vanderbilt University, Nashville, TN, United States 37232

[5]Department of Chemistry
Vanderbilt University, Nashville, TN, United States 37232

Table of Contents

**Supporting Figures**	**S4-S10**
Figure S1: RMA-S Stabilization Assay Workflow	S4
Figure S2: RMA-S Stabilization Assay sarsWT Concentration Scan	S5
Figure S3: RMA-S Stabilization Assay 37 ^{o}C Time Scan	S6
Figure S4: RMA-S Stabilization compared to fitc ova competition assay	S7
Figure S5: LC-MS analysis of ovaWT and spiked internal standard peptides	S8
Figure S6: Collision-activated dissociation (CAD) tandem mass spectrometry (MS/MS) of ovaWT	S8
Figure S7: LC-MS analysis of ovaKm3 and spiked internal standard peptides of ovaK7m3	S9
Figure S8: Collision-activated dissociation (CAD) tandem mass spectrometry (MS/MS) of ovaK7m3	S9
Figure S9: DC2.4 T Cell Activation	S10
Figure S10: Heat Map RMA-S Stabilization of Cancer Antigens	S11
Figure S11: Modeling the peptide mbp with and without citrullination of the N-terminus arginine residue	S12
**Materials and Methods**	**S13**
Materials	S13
Computational Methods	S13
**Synthesis and Characterization**	**S15-S84**
Scheme S1: SIINFEKL	S15
Scheme S2: Monomethyl Lysine SIINFKEL	S17
Scheme S3: Dimethyl Lysine SIINFKEL	S19
Scheme S4: Trimethyl Lysine SIINFKEL	S21
Scheme S5: Succinyl Lysine SIINFKEL	S23
Scheme S6. Acetyl Lysine SIINFKEL	S25
Scheme S7: Biotinylated Lysine SIINFKEL	S27
Scheme S8: Phosphoserine SIINFEKL	S29

Scheme S9: SNFVSAGI	S31
Scheme S10: ESIVRFPNI	S33
Scheme S11: N-Acetyl ESIVRFPNI	S35
Scheme S12: Citrullinated ESIVRFPNI	S37
Scheme S13: Hydroxy Proline ESIVRFPNI	S39
Scheme S14: YNVRKSEM	S41
Scheme S15: YNVcitKSEM	S43
Scheme S16: RIYQYIQSRF	S45
Scheme S17: RIYQ(YPO4)IQSRF	S47
Scheme S18: HPDKFVGI	S49
Scheme S19: HPD(Kac)FVGI	S51
Scheme S20: ANLERTF	S53
Scheme S21: NAc-ANLERTF	S55
Scheme S22: SAIQNHSF	S57
Scheme S23: NAc-SAIQNHSF	S59
Scheme S24: TVFVFKRA	S61
Scheme S25: TVFVFKcitA	S63
Scheme S26: SGIAKTAL	S65
Scheme S27: SGIA(Kac)PLVL	S67
Scheme S28: SNPKPLVL	S69
Scheme S29: SNP(Kac)PLVL	S71
Scheme S30: VVYPWTQRF	S73
Scheme S31: VVYPWTQcitF	S75
Scheme S32: RQYDKFLTHF	S77
Scheme S33: citQYDKFLTHF	S79
Scheme S34: RSPSPKTSL	S81
Scheme S35: RSP(SPO4)PKTSL	S83

SUPPORTING FIGURES

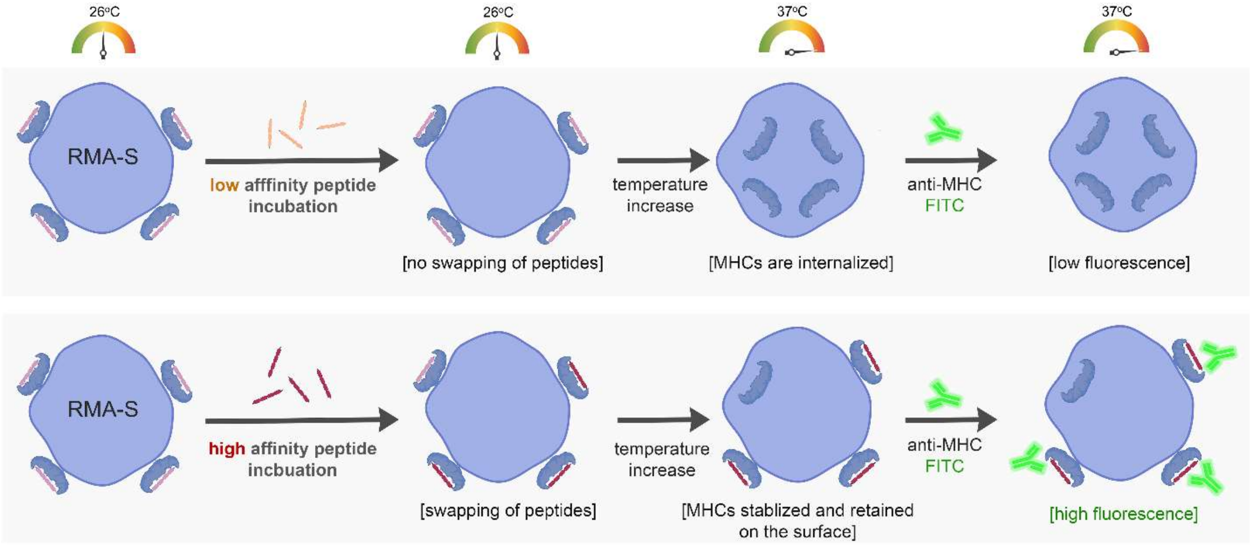

Figure S1. RMA-S stabilization assay workflow. RMA-S cells express low affinity peptides on their surface at 26 °C. When the temperature is raised to 37 °C, in the absence of a high affinity MHC binding peptide (top) the low affinity pMHC complex dissociates and the empty MHC is internalized and degraded. In the presence of a high affinity binder (bottom) the pMHC complex remains stable at 37 °C and can be detected via fluorescently labeled anti-MHC antibodies.

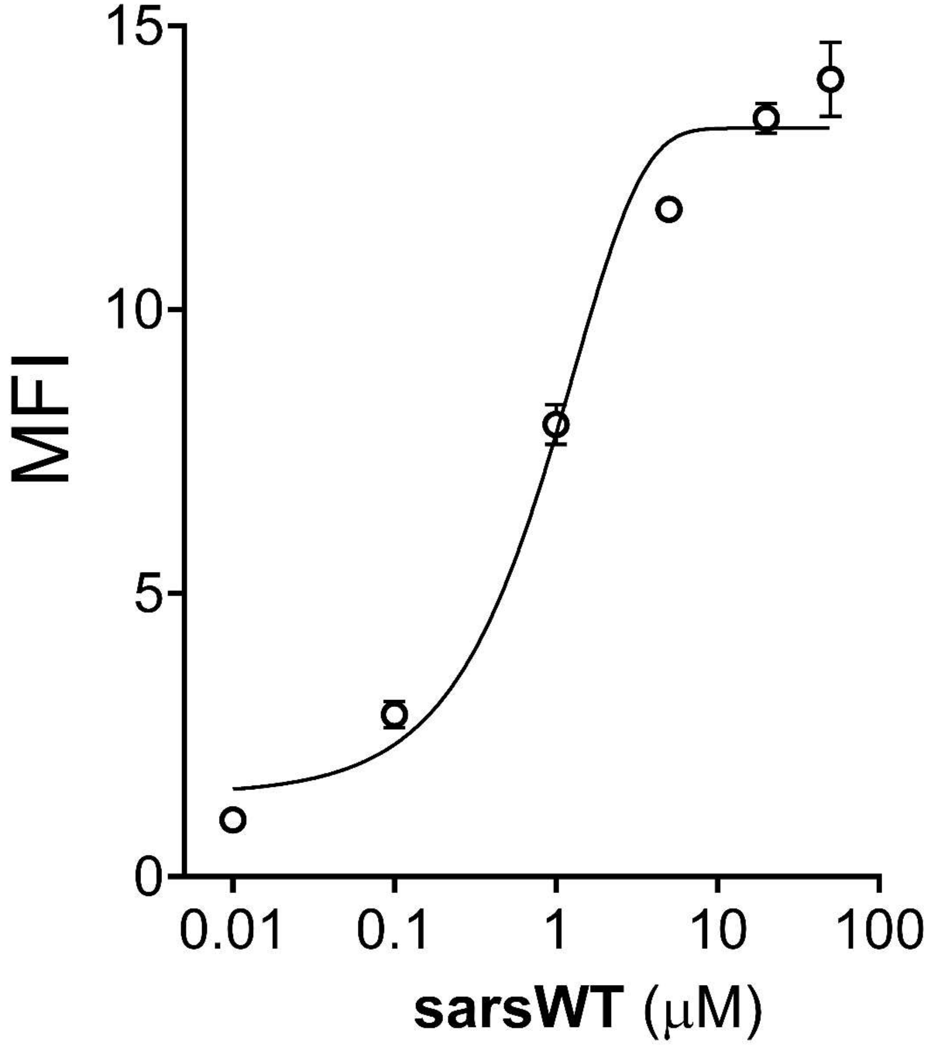

Figure S2. Flow cytometry analysis of RMA-S cells treated with indicated concentration of **sarsWT** detected by APC conjugated anti-mouse H-2K^b antibody. Data are represented as mean ± SD (n= 3). P-values were determined by a two-tailed *t*-test (* $p < 0.05$, ** $p < 0.01$, *** $p < 0.001$, **** $p < 0.0001$, ns = not significant).

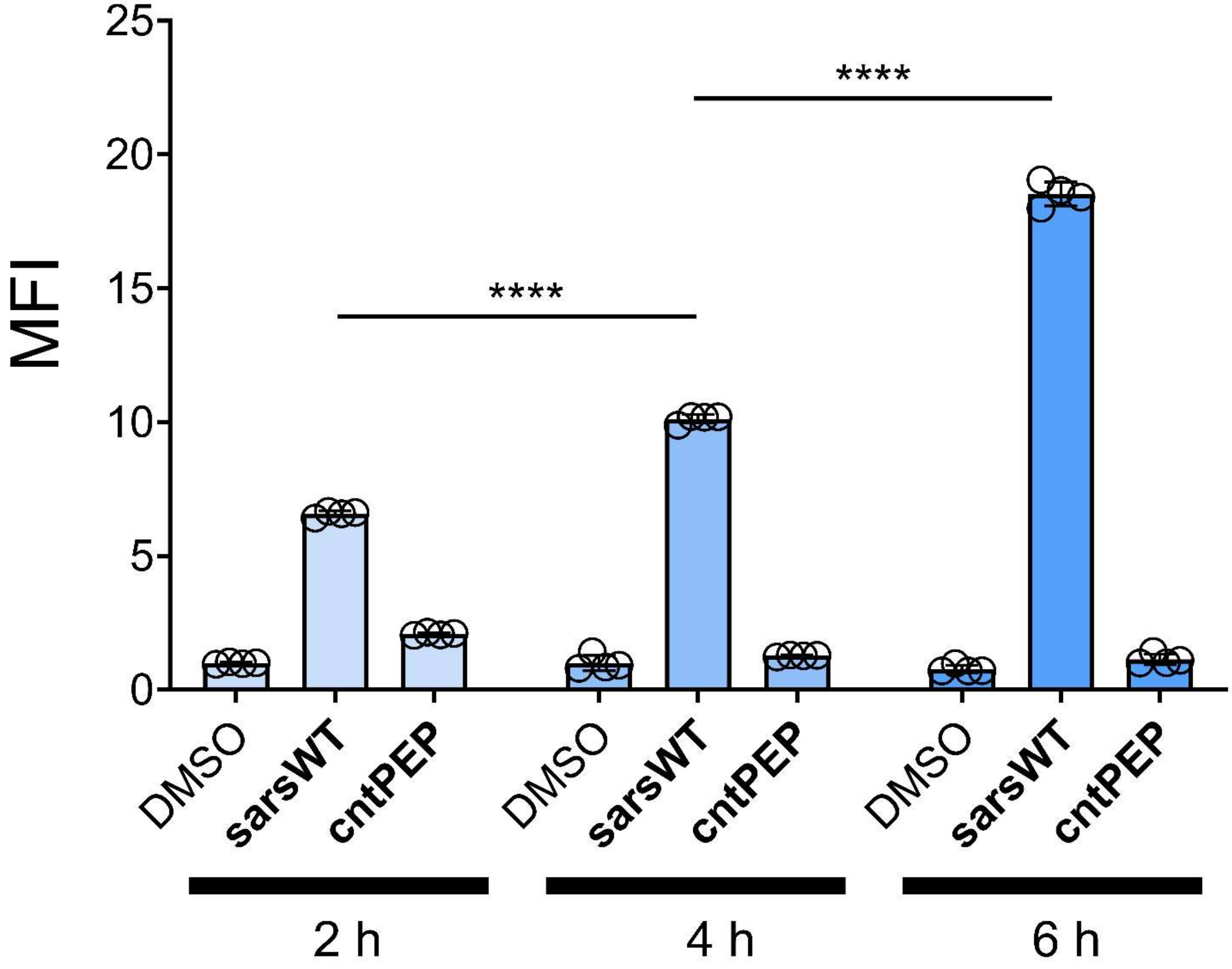

Figure S3. Flow cytometry analysis of RMA-S cells treated with of **sarsWT** (20 μM) for indicated time points detected by APC conjugated anti-mouse H-2K^b antibody. Data are represented as mean ± SD (n= 3). P-values were determined by a two-tailed *t*-test (* p < 0.05, ** p < 0.01, *** p < 0.001, **** p < 0.0001, ns = not significant).

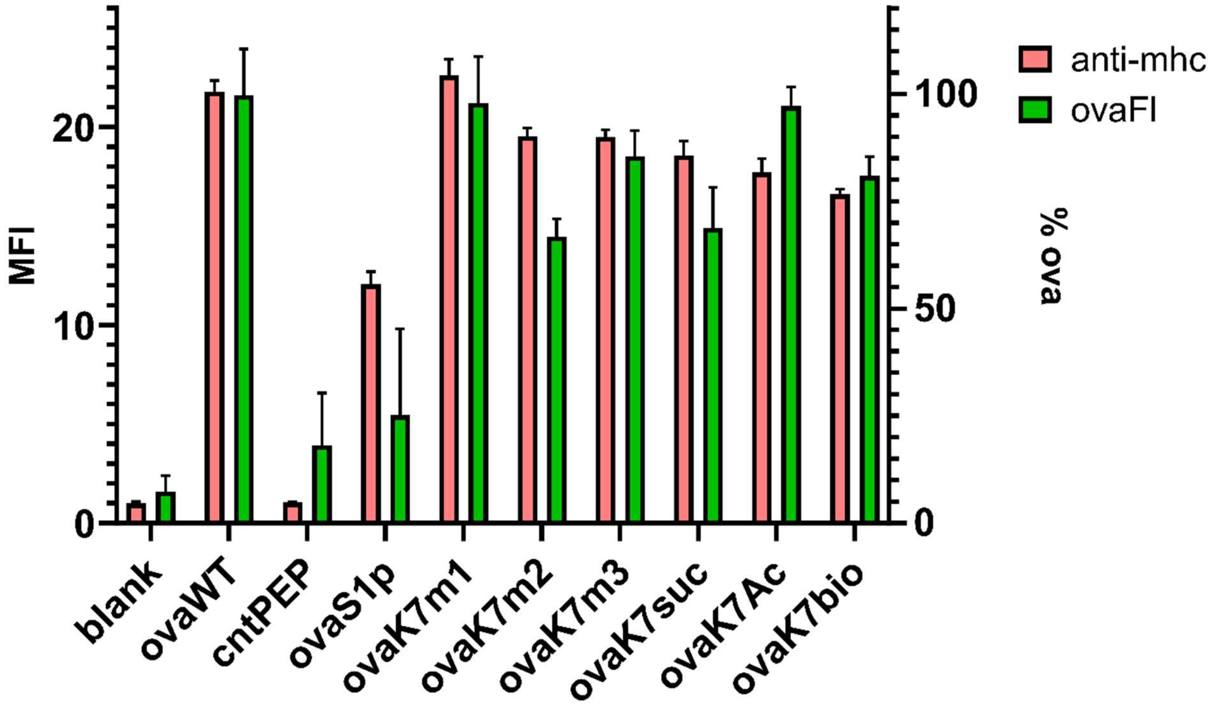

Figure S4. Flow cytometry analysis of RMA-S cells treated with indicated peptide and detected by APC conjugated anti-mouse H-2K^b antibody (red). RMA cells were co-incubated with ovaFI (4 μM) and excess of indicated peptide (32 μM) (green). Data is represented as a percent decrease in fluorescence relative to ovaWT. Data are represented as mean ± SD (n= 3).

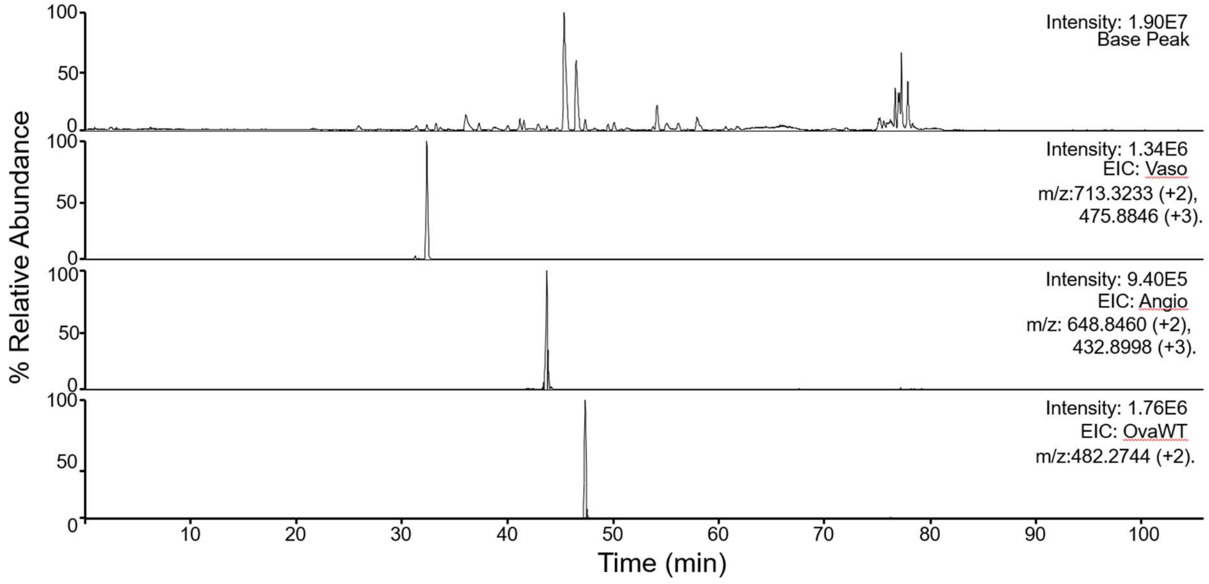

Figure S5: LC-MS analysis of **ovaWT** and spiked internal standard peptides. Extracted ion chromatograms (EIC) are shown for the base peak and selected ion m/z corresponding to the ovaWT, 100 fmol each of Vaso and Angio peptides.

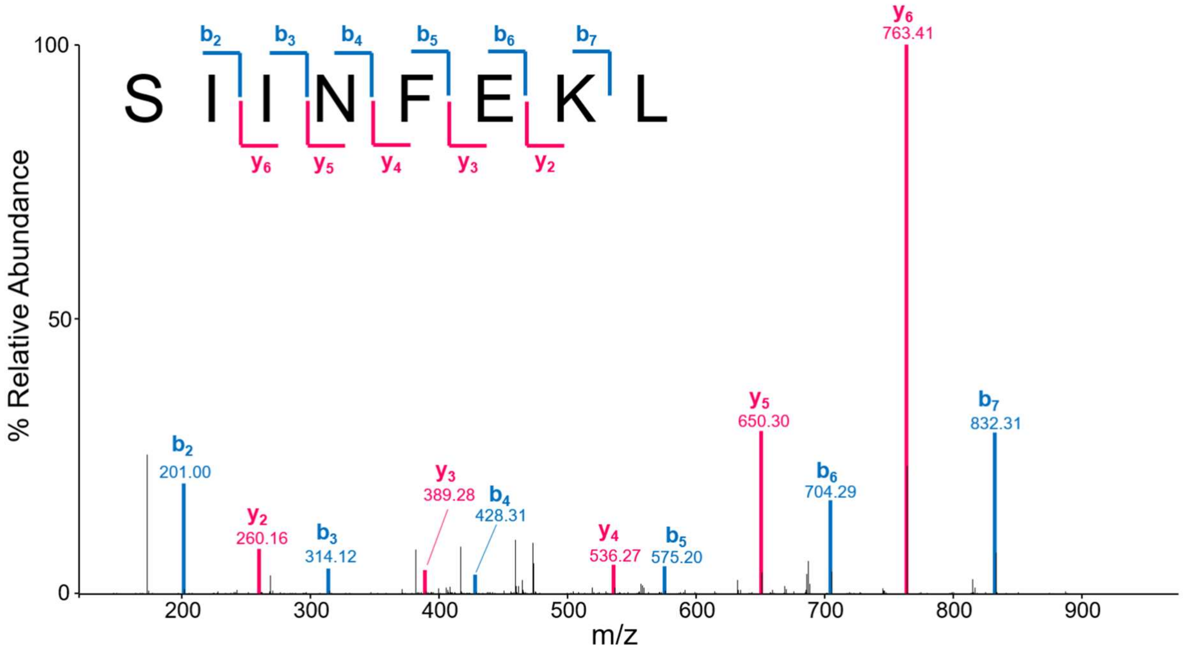

Figure S6. Collision-activated dissociation (CAD) tandem mass spectrometry (MS/MS) spectrum acquired in the ion trap showing fragment masses of **ovaWT** [M+2H]2+ precursor ion with m/z 482.2744. Sequence coverage for b+ ion fragments are labeled in blue, and y+ ion fragments are labeled in pink. The identified ions are sufficient for complete sequence coverage.

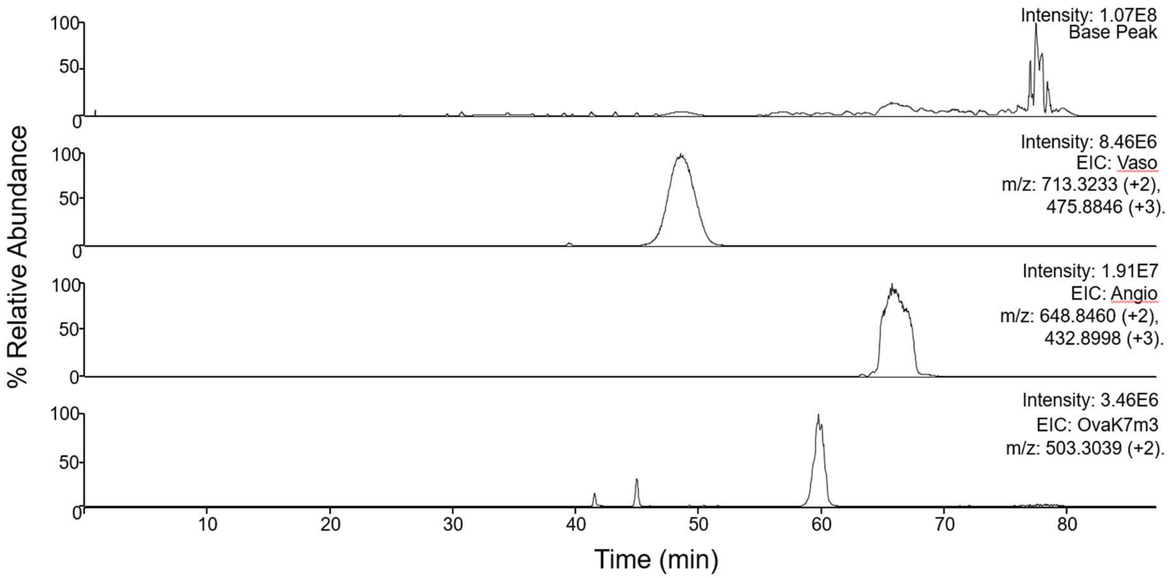

Figure S7: LC-MS analysis of **ovaK7m3** and spiked internal standard peptides. Extracted ion chromatograms (EIC) are shown for the base peak and selected ion m/z corresponding to the ovaK7m3, 100 fmol each of Vaso and Angio peptides.

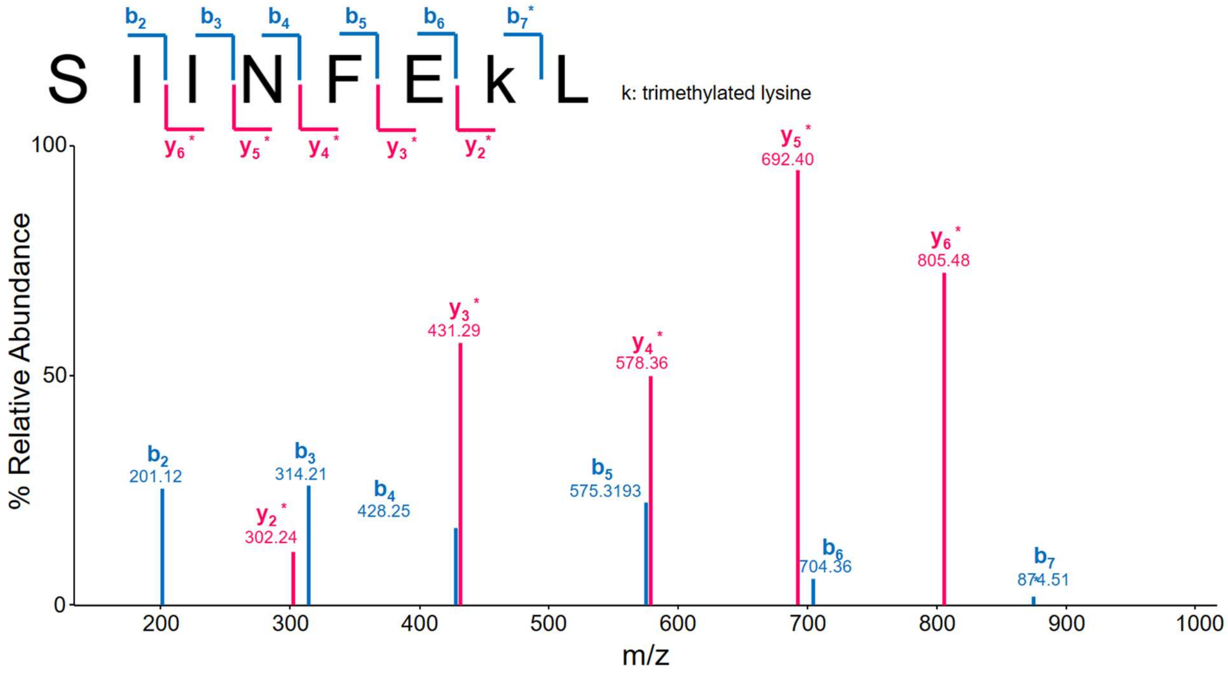

Figure S8. Collision-activated dissociation (CAD) tandem mass spectrometry (MS/MS) spectrum acquired in the ion trap showing fragment masses of **ovaK7m3** [M+2H]2+ precursor ion with m/z 482.2744. Sequence coverage for b+ ion fragments are labeled in blue, and y+ ion fragments are labeled in pink. The identified ions are sufficient for complete sequence coverage.

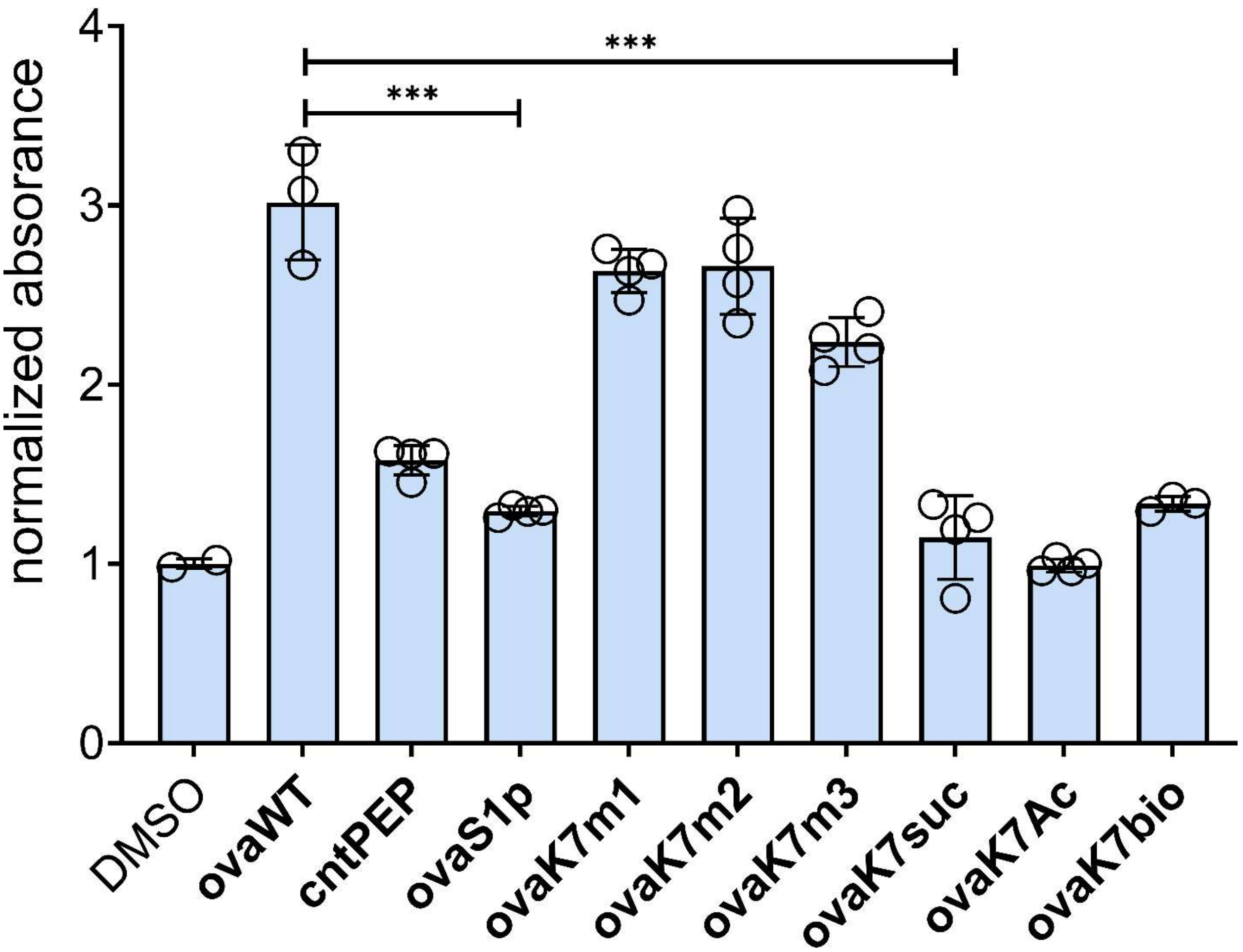

Figures S9. DC2.4 cells were incubated with peptide and B3Z T-cells overnight at an effector to target ratio of 1:1. β-galactosidase expression was then measured via the colorimetric reagent CPRG on a plate reader at 570 nm.

A

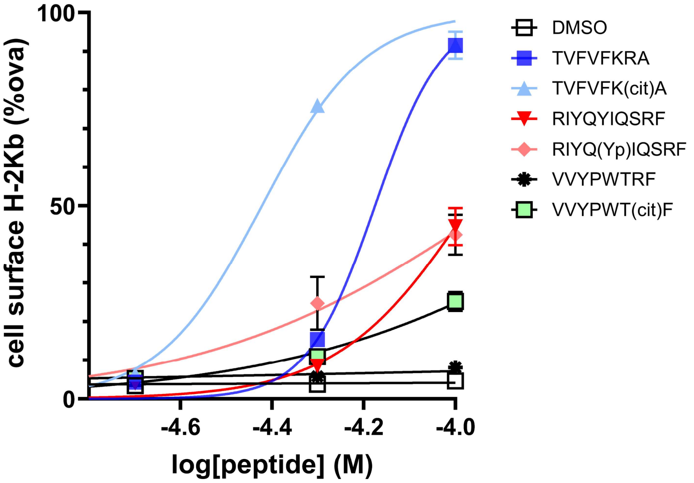

B

Peptide	log(EC50)
TVFVFKRA	-4.175 ± 0.022
TVFVFK(cit)A	-4.422 ± 0.038
RIYQYIQSRF	-3.968 ± 0.034
RIYQ(Yp)IQSRF	-2.393 ± 0.100
VVYPWTRF	2.338 ± 4.363
VVYPWT(CIT)F	-3.616 ± 0.174

Figure S10. (A) Dose response-curve of WT and PTM modified peptides using the RMA-S stabilization assay. RMA-S cells were incubated with peptide at indicated concentration of peptides and detected via flow cytometry with APC conjugated anti-mouse H-$2K^b$ antibodies. (B) Table of log(EC50) values. Abbreviations: Cit (citrullination), Yp (phosphorylated tyrosine).

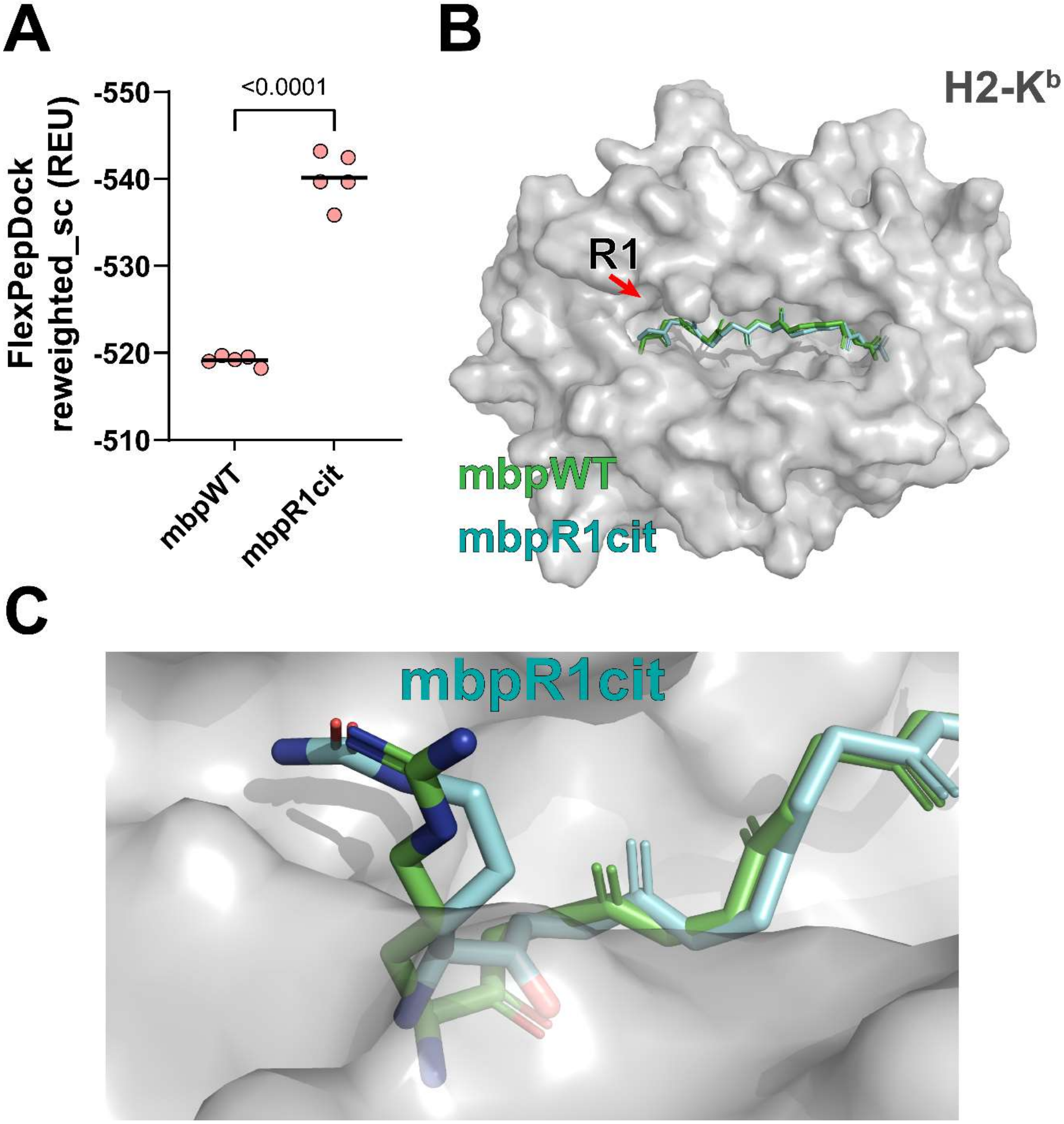

Figure S11. Modeling the peptide mbp with and without citrullination of the N-terminus arginine residue. (A) Arginine citrullination generates models with a higher average reweighted_sc, consistent with experimental results showing citrullination enhances binding affinity (two-tailed student's t test; individual values correspond to the top 0.5% of models generated, and bars represent mean score term values). (B) Superimposed backbones for the top model generated for the mbpWT and mbpR1cit variants show little difference in overall peptide configuration. (C) Detailed side-chain configuration for both the mbpWT and mbpR1cit variant at the N-terminal arginine residue.

Materials.

All peptide related reagents and protected amino acids were purchased from Chem-Impex. APC-labeled anti-mouse H-2K^d/H-2D^d antibody was purchased from Biolegend. Pooled Human Serum and acetic acid (glacial, >99.99% trace metals basis), and penicillin-streptomycin was purchased from Sigma Aldrich. Dulbecco's Modified Eagle's Medium (DMEM) was purchased from VWR. Fetal Bovine Serum (FBS) was purchased from R&D Systems. Angiotensin II phosphate > 95% HPLC and vasoactive intestinal peptide (VIP) 1-12 human, porcine, rat was purchased from AnaSpec. Pierce^{TM} Waters, LC/MS grade was purchased from Thermo Scientific. Methanol, Optima^{TM} LC/MS grade was purchased from Fisher Chemical. Acetonitrile, B&J Brand^{TM} LC-MS, for LC-MS and HPLC, >99.9% was purchased from Honeywell. Kasil 1624 potassium silicate solution was purchased from PQ Corporation. 100 µm i.d. x 360 µm o.d. Polyimide coated fused silica nano-capillary tubing and 75 µm i.d. x 360 µm o.d. Polyimide coated fused silica nano-capillary tubing were purchased from PolyMicro Technologies, Inc. Reprosil Pur 120 C18 AQ 3 μm and Reprosil Pur 120 C18 AQ 10 μm were purchased from Dr Maisch GMBH. Mode 5424 Centrifuge and Protein LoBind microcentrifuge tubes were purchased from Eppendorf. Teflon tubing, 0.012-inch i.d. x 0.060-inch 0.d. was purchased from Zeus Industrial Products, Inc. P-2000 microcapillary laser puller with fused silica adapter was purchased from Sutter Instrument Co. LTQ-Orbitrap mass spectrometer was purchased from Thermo-Fisher Scientific. All other organic chemical reagents were purchased from Fisher Scientific or Sigma Aldrich and used without further purification. ALL COMPOUNDS ARE >95% PURE BY HPLC ANALYSIS.

Experimental Methods.

Computational Methods

We used the FlexPepDock refinement application in combination with ROSETTA scripts to simulate peptide docking to H-2K^b in ROSETTA 3.13.[1,2] FlexPepDock refinement has successfully recapitulated peptide/MHC-I complex structures with sub-angstrom accuracy,[3] and we have used this protocol to generate peptide/MHC-I structures for peptides containing post-translational modifications and other non-canonical amino acids.[4] The refinement protocol requires initial templates that approximate the final peptide configuration. We identified templates by scoring sequence alignments between the peptide to model and H2-K^b bound peptides with structures available in the PDB. The H-2K^b MHC-1 model was generated using AlphFold2.[5] Residues in the template peptide were sequentially mutated to match those of the peptide to model using the ROSETTA mover MutateResidue. For modeling residues with post-translational modifications, we applied either pre-existing patches with the ROSETTA mover ModifyVariantType or created *de-novo* parameter files and rotamer libraries as described (see supplemental

table SX).[6] For each docking simulation 1000 models were created. Models were sorted by the reweighted_sc statistic (a modified ROSETTA energy score term that doubles contributions from interface residues and triples contributions from peptide residues) and the top 0.5% scoring models selected for further analysis. We previously validated this statistic as a metric for peptide binding affinity using a variant of the FlexPepBind protocol as described by Alam et al.[4,7] Computations were carried out with resources provided by the Vanderbilt Advanced Computing Center for Research and Education (ACCRE). Top-scoring models were rendered and visually inspected using the Pymol Molecular Graphics System v2.0 by Schrödinger, LLC.

Scheme S1. Synthesis of SIINFKEL

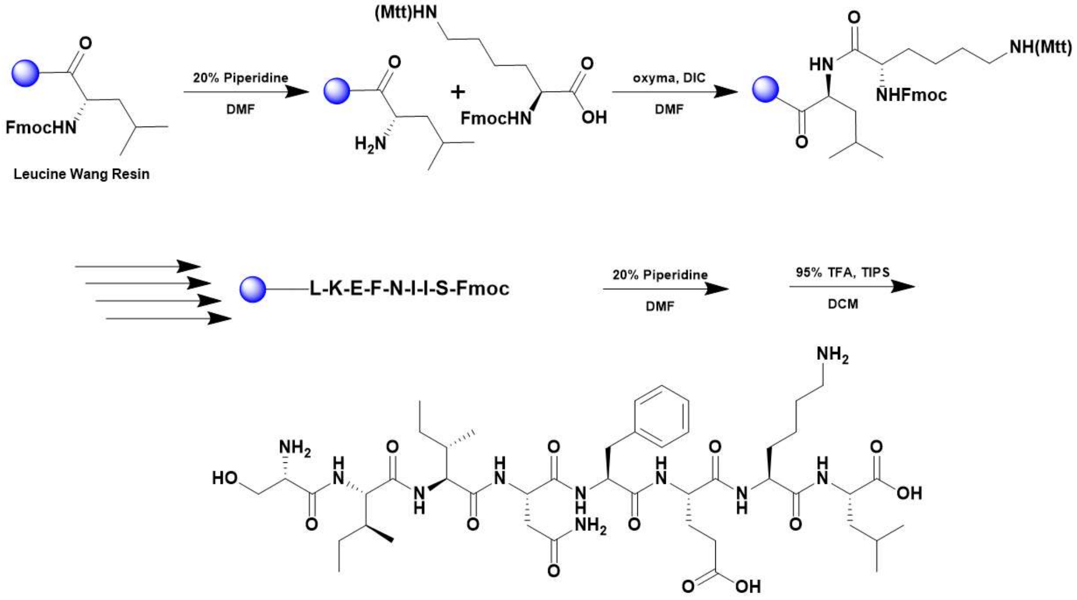

A 25 mL vessel of CEM discover bio manual peptide synthesizer was charged with 0.25 mmol of leucine wang resin. The Fmoc group was removed by using a 20% piperidine solution in DMF (10 mL). Using Synergy software, the deprotection protocol was run. The piperidine solution was drained and the resin was washed with DMF (4 x 10 mL). Fmoc-L-lysine(Mtt)-OH (5 eq, 1.25 mM) along with Oxyma (5 eq, 1.25 mM) and DIC (5 eq, 1.35 mmol) in DMF was added to the reaction vessel and the coupling protocol was run. The amino acid solution was drained, and the resin was washed with DMF (2 x 10 mL). The fmoc removal and coupling procedure was repeated as before using the same equivalencies for the remaining amino acids. To remove the peptide from resin, a TFA cocktail solution (95% TFA, 2.5% TIPS, and 2.5% DCM) was added to the resin and agitated for 2 hours. The resin was filtered, and the resulting solution was concentrated in vacuo. The peptide was triturated with cold diethyl ether and purified using reverse phase HPLC using H_2O/CH_3CN. The sample was analyzed for purity using a Waters 1525 Binary HPLC Pump using a Phenomenex Luna 5u C8(2) 100A (250 x 4.60 mm) column; gradient eluted with H_20/CH_3CN. Molecular weight was confirmed using high resolution electrospray ionization mass spectrometry (HRMS, ESI/MS) analyses obtained on an Agilent 6545B Q-TOF LC/MS equipped with 1260 infinity II LC system with auto sampler. The final peptide product was lyophilized and stored at -20°C until further use.

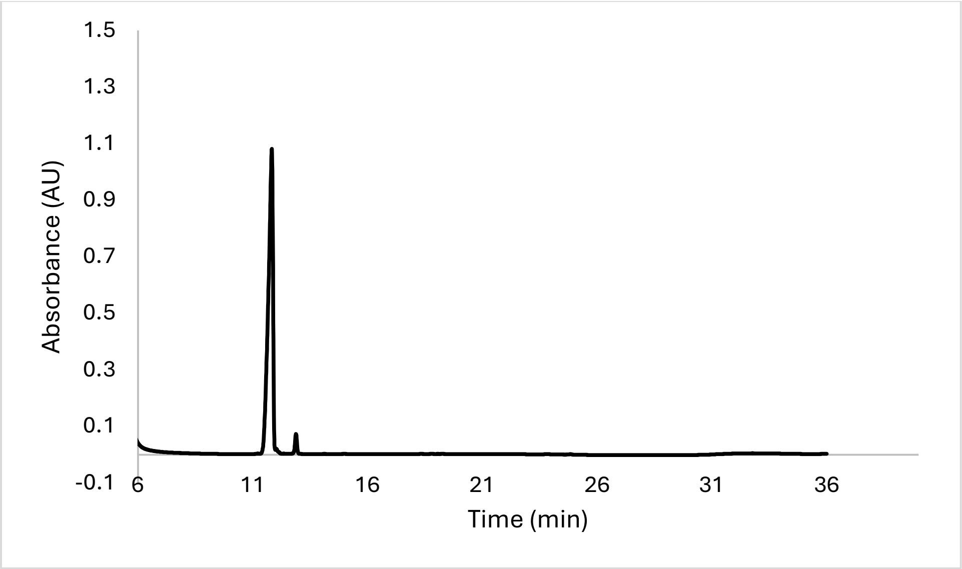

ESI-MS calculated [M+H^{+}]: 963.5515, found 963.5503.

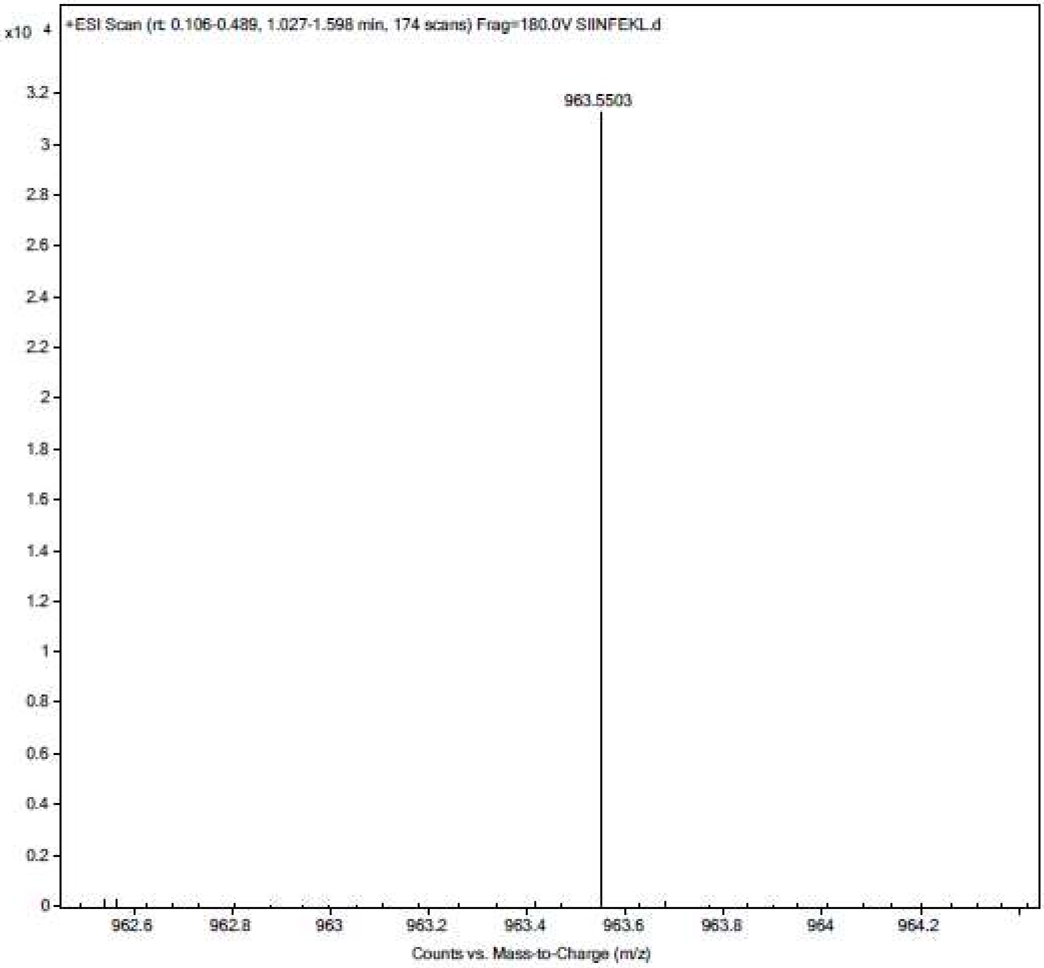

Scheme S2. Synthesis of Monomethyl Lysine SIINFKEL

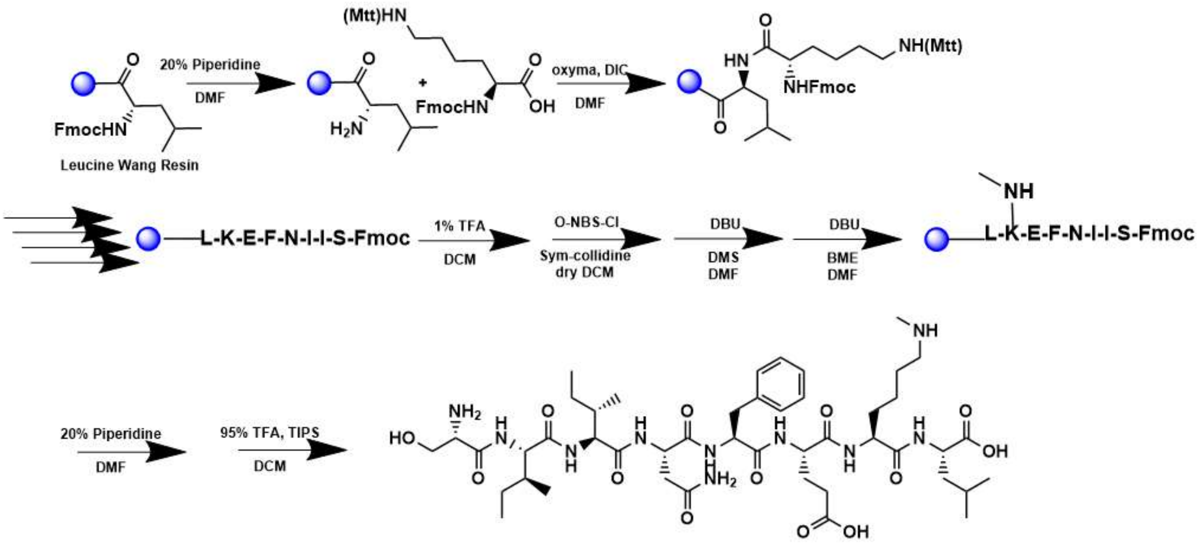

A 25 mL vessel of CEM discover bio manual peptide synthesizer was charged with 0.25 mmol of leucine wang resin. The Fmoc group was removed by using a 20% piperidine solution in DMF (10 mL). Using Synergy software, the deprotection protocol was run. The piperidine solution was drained and the resin was washed with DMF (4 x 10 mL). Fmoc-L-lysine(Mtt)-OH (5 eq, 1.25 mM) along with Oxyma (5 eq, 1.25 mM) and DIC (5 eq, 1.35 mmol) in DMF was added to the reaction vessel and the coupling protocol was run. The amino acid solution was drained, and the resin was washed with DMF (2 x 10 mL). The fmoc removal and coupling procedure was repeated as before using the same equivalencies for the remaining amino acids. The MTT protecting group was removed by the addition of 1% TFA, 2.5% TIPS, in 10 mL DCM for 15 min, washed and repeated 5 more times. O-NBS-Cl (4 eq, 1 mM) was added along with sym-collidine (10 eq, 2.5 mM) in dry DCM and treated with resin for 15 mins while agitated for a total of 2x. The solution was drained and DBU (3 eq, 0.75 mM) and DMS (10 eq, 2.5 mM) in DMF was added and agitated for 5 mins. The solution was drained and BME (10 eq, 2.5 mM) and DBU (5 eq, 1.25 mM) we added in DMF, and the solution was agitated for 30 mins.To remove the peptide from resin, a TFA cocktail solution (95% TFA, 2.5% TIPS, and 2.5% DCM) was added to the resin and agitated for 2 hours. The resin was filtered, and the resulting solution was concentrated in vacuo. The peptide was trituated with cold diethyl ether and purified using reverse phase HPLC using H_2O/CH_3CN. The sample was analyzed for purity using a Waters 1525 Binary HPLC Pump using a Phenomenex Luna 5u C8(2) 100A (250 x 4.60 mm) column; gradient eluted with H_20/CH_3CN. Molecular weight was confirmed using high resolution electrospray ionization mass spectrometry (HRMS, ESI/MS) analyses obtained on an Agilent 6545B Q-TOF LC/MS equipped with 1260 infinity II LC system with auto sampler. The final peptide product was lyophilized and stored at -20°C until further use.

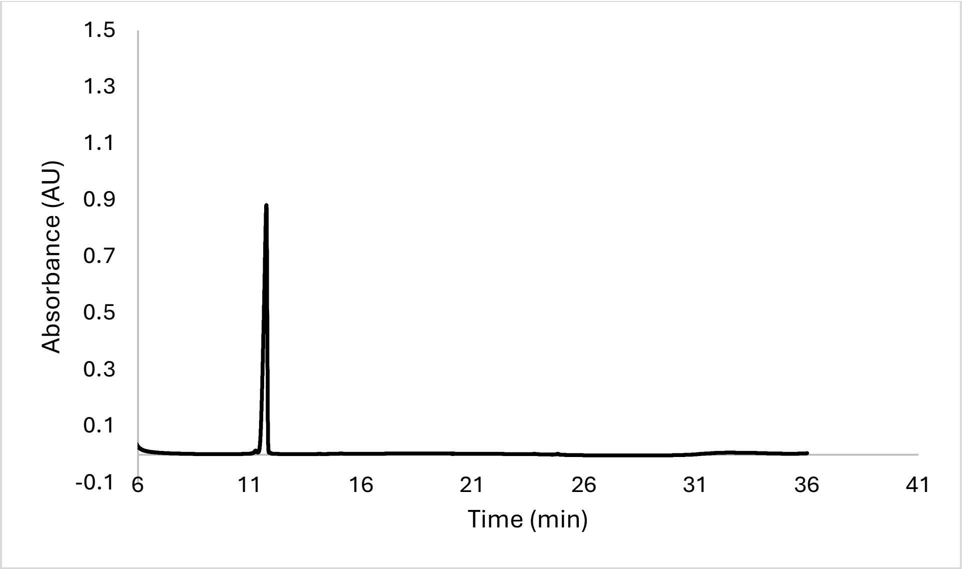

ESI-MS calculated [M+H$^+$]: 977.5671, found 977.5666.

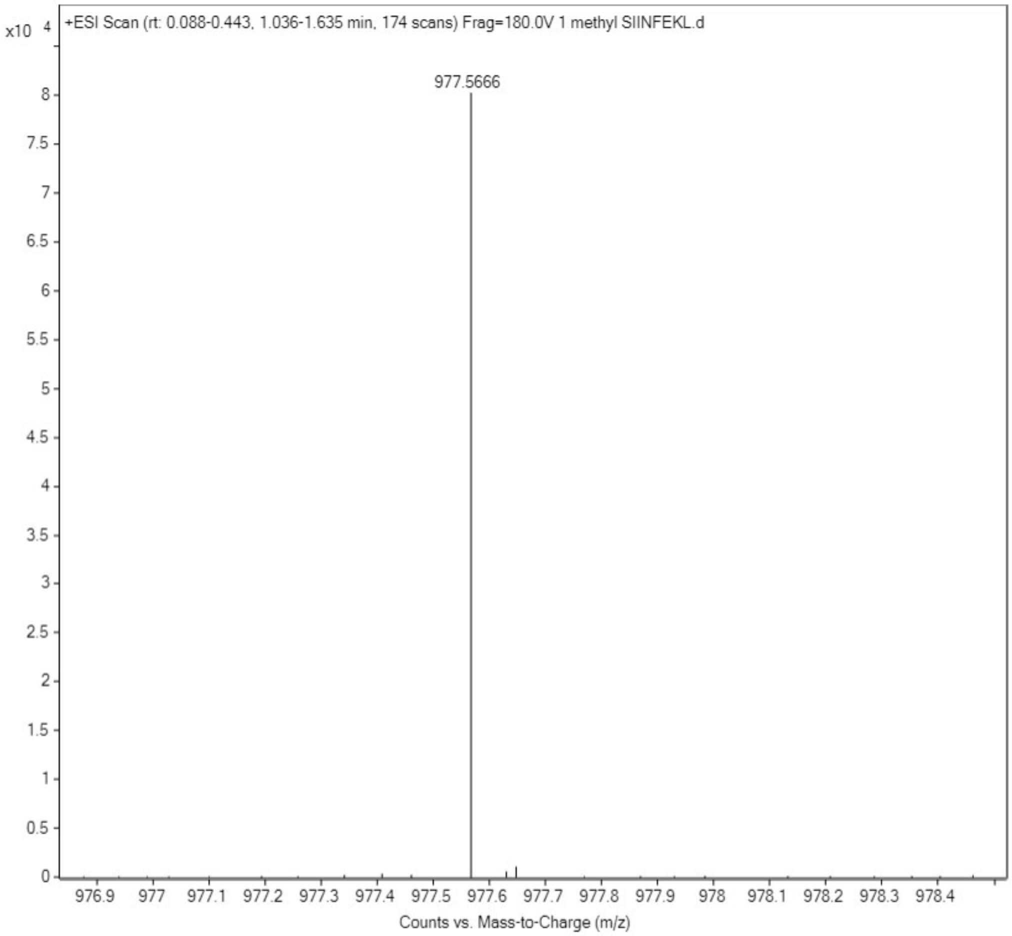

Scheme S3. Synthesis of Dimethyl Lysine SIINFKEL

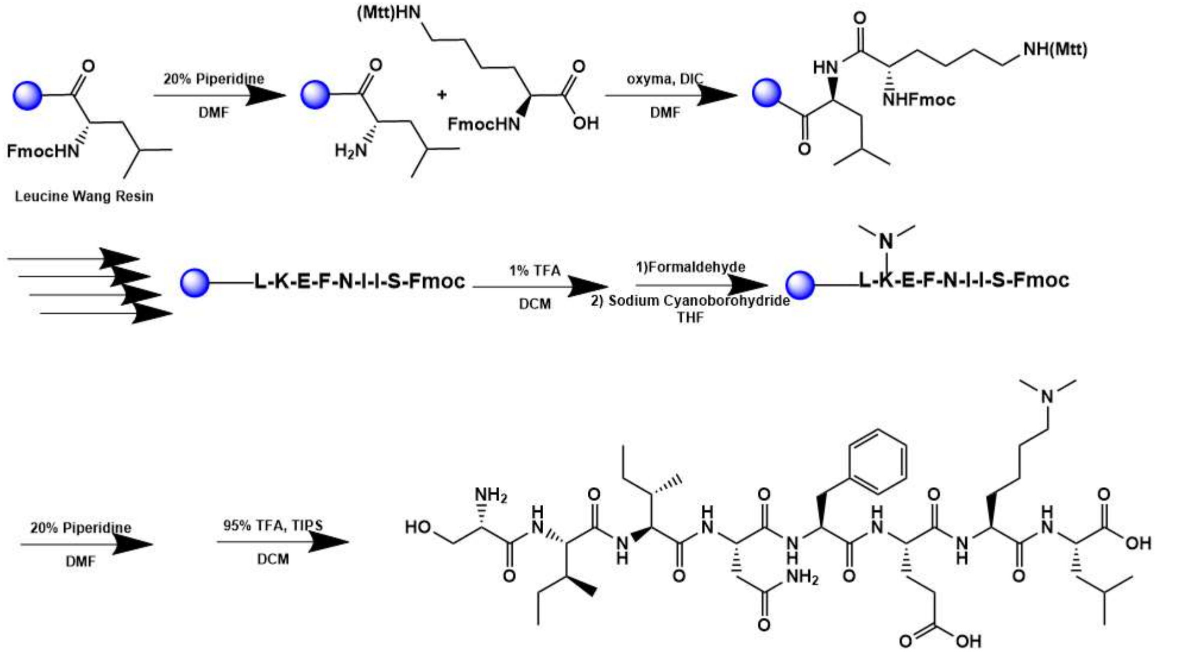

A 25 mL vessel of CEM discover bio manual peptide synthesizer was charged with 0.25 mmol of leucine wang resin. The Fmoc group was removed by using a 20% piperidine solution in DMF (10 mL). Using Synergy software, the deprotection protocol was run. The piperidine solution was drained and the resin was washed with DMF (4 x 10 mL). Fmoc-L-lysine(Mtt)-OH (5 eq, 1.25 mM) along with Oxyma (5 eq, 1.25 mM) and DIC (5 eq, 1.35 mmol) in DMF was added to the reaction vessel and the coupling protocol was run. The amino acid solution was drained, and the resin was washed with DMF (2 x 10 mL). The fmoc removal and coupling procedure was repeated as before using the same equivalencies for the remaining amino acids. The MTT protecting group was removed by the addition of 1% TFA, 2.5% TIPS, in 10 mL DCM for 15 min, washed and repeated 5 more times. Peptide solution was reacted with formaldehyde (10 eq, 12.5 mM) in THF at pH 3 for 15 mins. Sodium cyanoborohydride (20 eq, 25 mM) was added to reaction vessel and reacted for 3 hours and washed 3x with DCM and methanol. To remove the peptide from resin, a TFA cocktail solution (95% TFA, 2.5% TIPS, and 2.5% DCM) was added to the resin and agitated for 2 hours. The resin was filtered, and the resulting solution was concentrated in vacuo. The peptide was triturated with cold diethyl ether and purified using reverse phase HPLC using H_2O/CH_3CN. The sample was analyzed for purity using a Waters 1525 Binary HPLC Pump using a Phenomenex Luna 5u C8(2) 100A (250 x 4.60 mm) column; gradient eluted with H_20/CH_3CN. Molecular weight was confirmed using high resolution electrospray ionization mass spectrometry (HRMS, ESI/MS) analyses obtained on an Agilent 6545B Q-TOF LC/MS equipped with 1260 infinity II LC system with auto sampler. The final peptide product was lyophilized and stored at -20°C until further use.

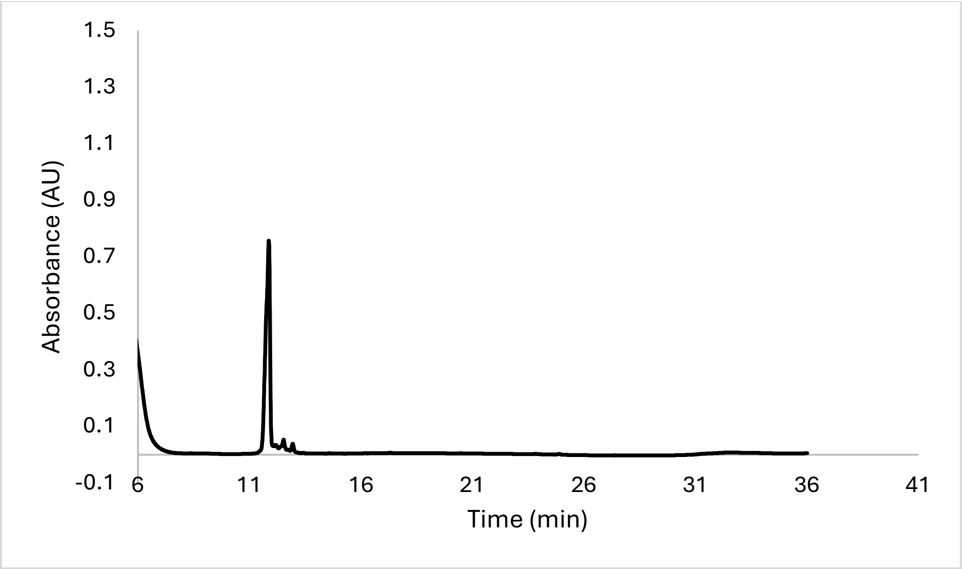

ESI-MS calculated [M+H$^+$]: 991.5828, found 991.5800.

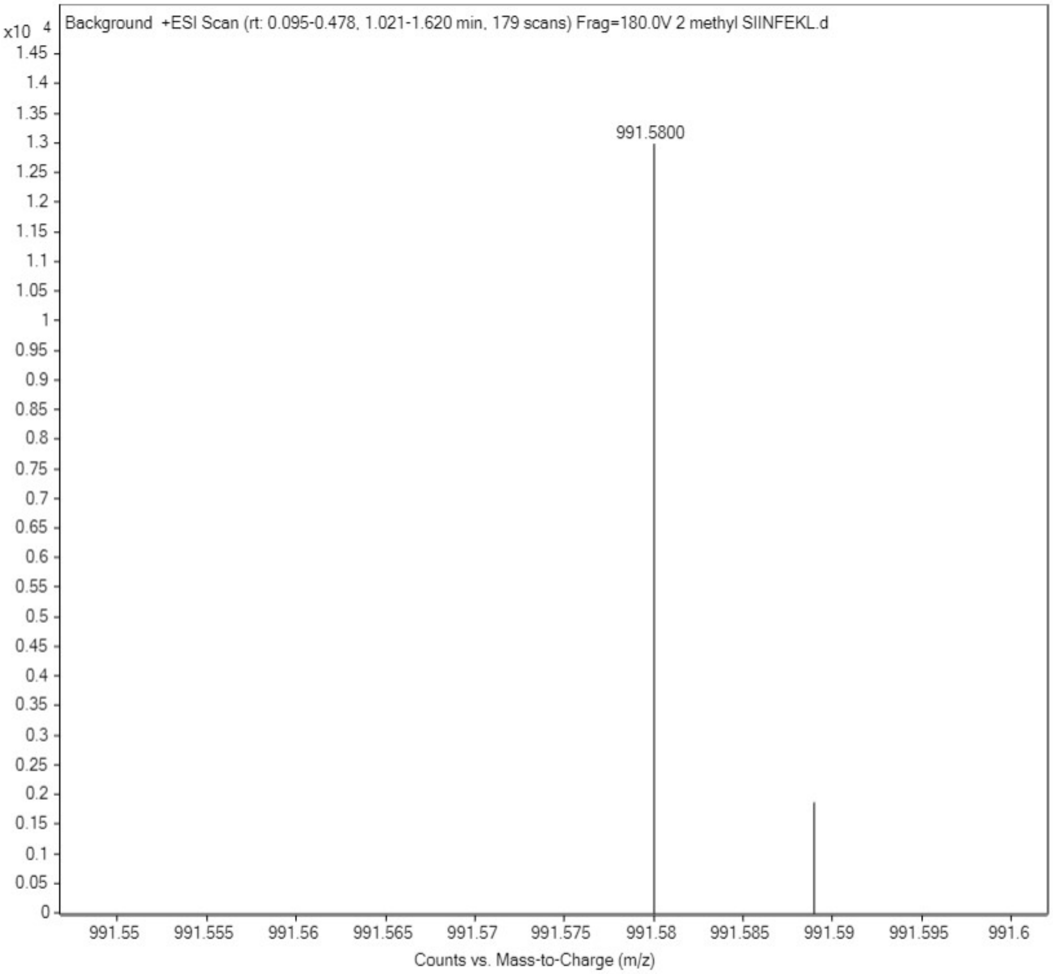

Scheme S4. Synthesis of Trimethyl Lysine SIINFKEL

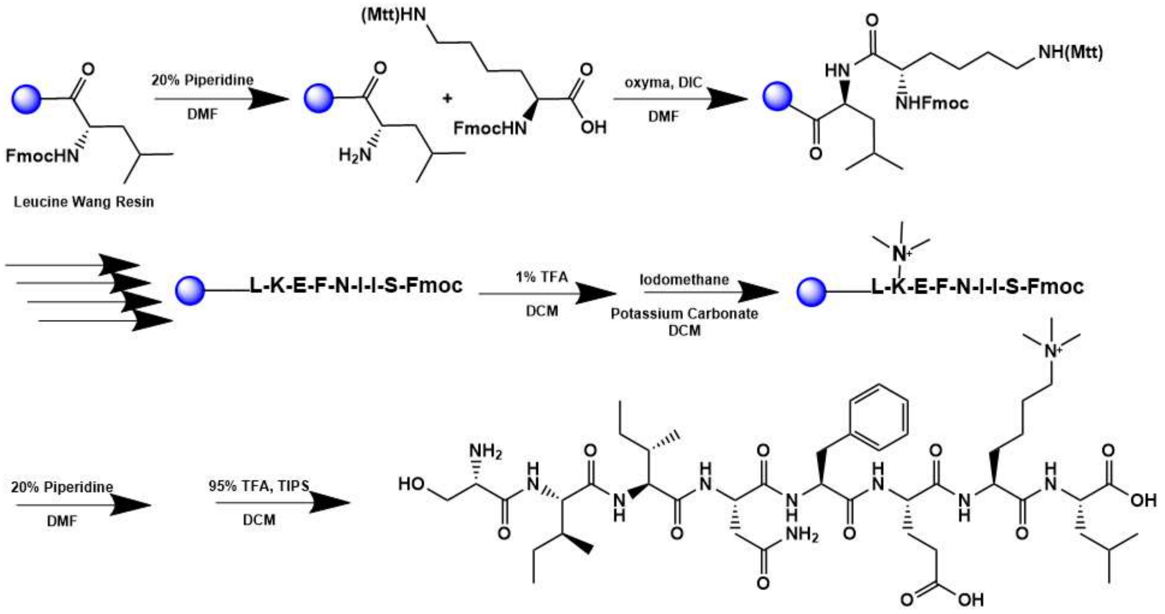

A 25 mL vessel of CEM discover bio manual peptide synthesizer was charged with 0.25 mmol of leucine wang resin. The Fmoc group was removed by using a 20% piperidine solution in DMF (10 mL). Using Synergy software, the deprotection protocol was run. The piperidine solution was drained and the resin was washed with DMF (4 x 10 mL). Fmoc-L-lysine(Mtt)-OH (5 eq, 1.25 mM) along with Oxyma (5 eq, 1.25 mM) and DIC (5 eq, 1.35 mmol) in DMF was added to the reaction vessel and the coupling protocol was run. The amino acid solution was drained, and the resin was washed with DMF (2 x 10 mL). The fmoc removal and coupling procedure was repeated as before using the same equivalencies for the remaining amino acids. The MTT protecting group was removed by the addition of 1% TFA, 2.5% TIPS, in 10 mL DCM for 15 min, washed and repeated 5 more times. Resin was transferred to a round bottom flask and stired in a solution of iodomethane (50 eq, 12.5 mM) and potassium carbonate (10 eq, 2.5 mM) in DCM and heated to 90°C overnight and repeated three times with fresh reagent. To remove the peptide from resin, a TFA cocktail solution (95% TFA, 2.5% TIPS, and 2.5% DCM) was added to the resin and agitated for 2 hours. The resin was filtered, and the resulting solution was concentrated in vacuo. The peptide was trituated with cold diethyl ether and purified using reverse phase HPLC using H_2O/CH_3CN. The sample was analyzed for purity using a Waters 1525 Binary HPLC Pump using a Phenomenex Luna 5u C8(2) 100A (250 x 4.60 mm) column; gradient eluted with H_20/CH_3CN. Molecular weight was confirmed using high resolution electrospray ionization mass spectrometry (HRMS, ESI/MS) analyses obtained on an Agilent 6545B Q-TOF LC/MS equipped with 1260 infinity II LC system with auto sampler. The final peptide product was lyophilized and stored at -20°C until further use.

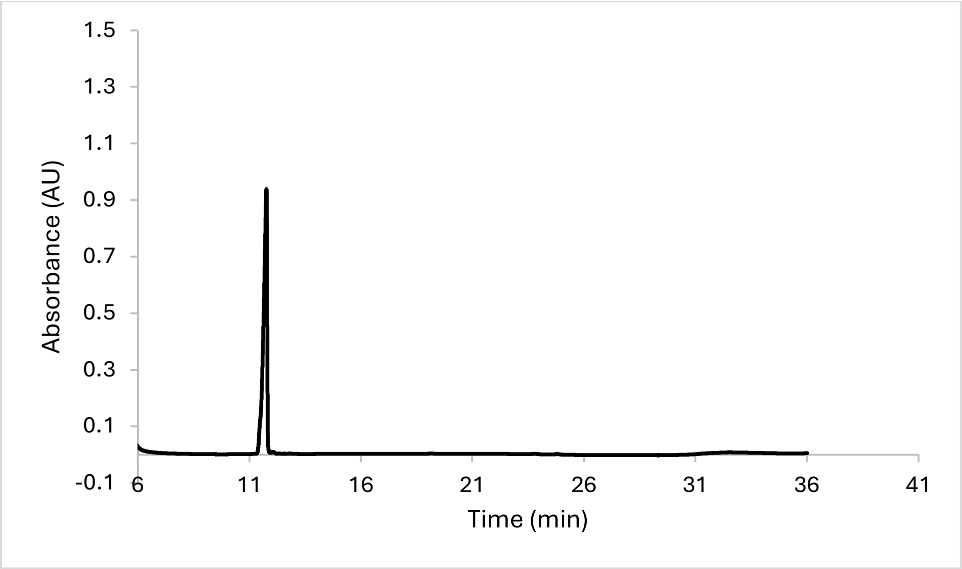

ESI-MS calculated [M+H$^+$]: 1006.6063, found 1006.6009.

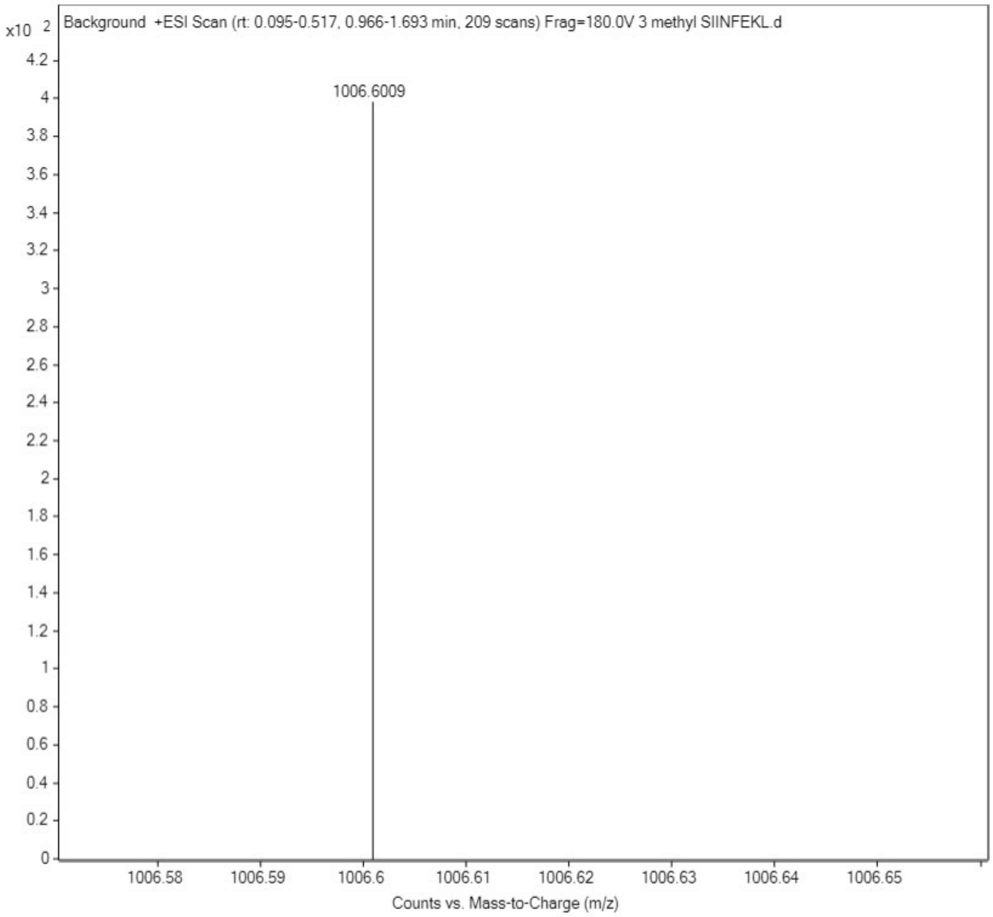

Scheme S5. Synthesis of Succinyl Lysine SIINFKEL

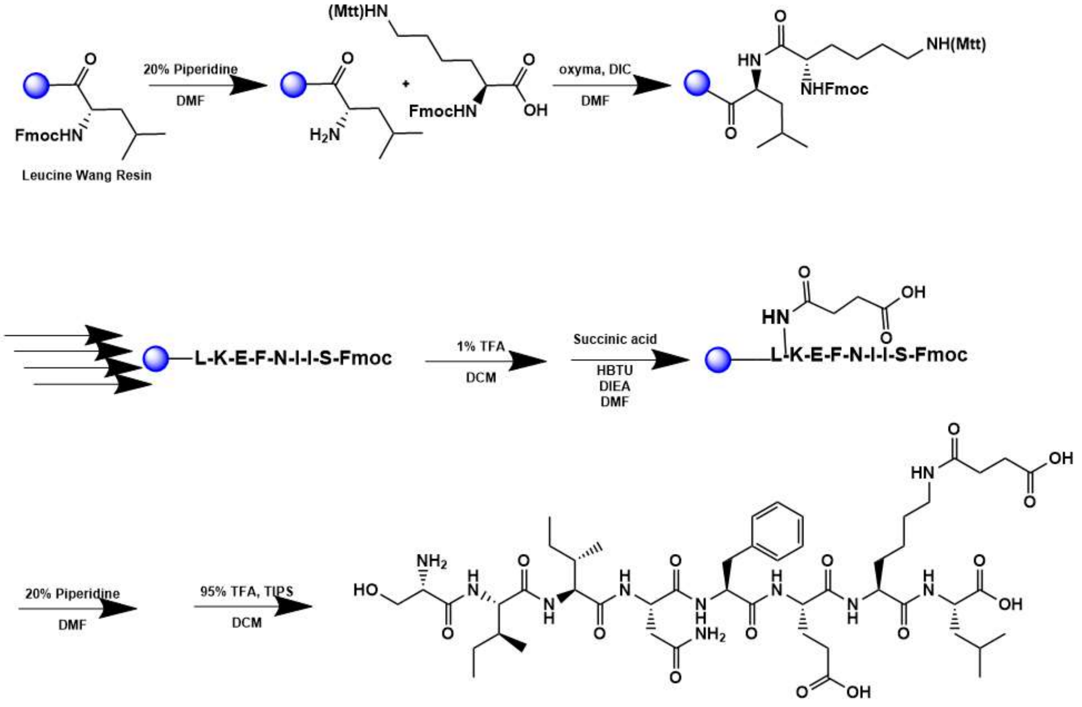

A 25 mL vessel of CEM discover bio manual peptide synthesizer was charged with 0.25 mmol of leucine wang resin. The Fmoc group was removed by using a 20% piperidine solution in DMF (10 mL). Using Synergy software, the deprotection protocol was run. The piperidine solution was drained and the resin was washed with DMF (4 x 10 mL). Fmoc-L-lysine(Mtt)-OH (5 eq, 1.25 mM) along with Oxyma (5 eq, 1.25 mM) and DIC (5 eq, 1.35 mmol) in DMF was added to the reaction vessel and the coupling protocol was run. The amino acid solution was drained, and the resin was washed with DMF (2 x 10 mL). The fmoc removal and coupling procedure was repeated as before using the same equivalencies for the remaining amino acids. The Mtt protecting group of L-Lysine(Mtt)-OH was removed by adding 10 mL of a TFA cocktail solution (1% TFA, 2% TIPS in DCM) to the resin and agitating for 10 minutes protected from light. The solution was drained, and this procedure was repeated five additional times. Succinic acid (8 eq, 2 mM) along with Oxyma (5 eq, 1.25 mM) and DIC (5 eq, 1.25 mmol) in DMF was added to the reaction vessel and agitated at room temperature for 2 hours. To remove the peptide from resin, a TFA cocktail solution (95% TFA, 2.5% TIPS, and 2.5% DCM) was added to the resin and agitated for 2 hours. The resin was filtered, and the resulting solution was concentrated in vacuo. The peptide was trituated with cold diethyl ether and purified using reverse phase HPLC using H_2O/CH_3CN. The sample was analyzed for purity using a Waters 1525 Binary HPLC Pump using a Phenomenex Luna 5u C8(2) 100A (250 x 4.60 mm) column; gradient eluted with H_20/CH_3CN. Molecular weight was confirmed using high resolution electrospray ionization mass spectrometry (HRMS, ESI/MS) analyses obtained on an Agilent 6545B Q-TOF LC/MS equipped with 1260 infinity II LC system with auto sampler. The final peptide product was lyophilized and stored at -20°C until further use.

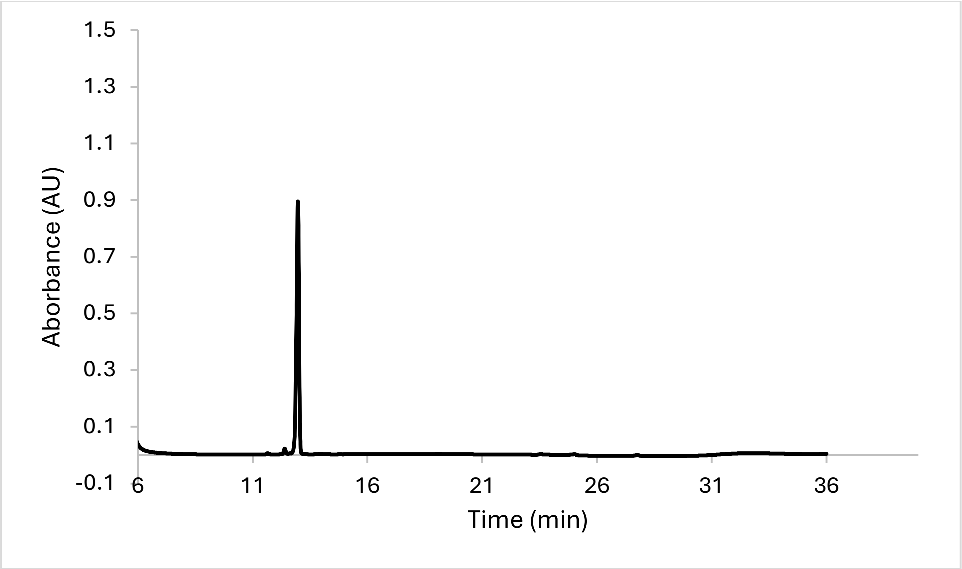

ESI-MS calculated [M+H$^+$]: 1063.5675, found 1063.5656.

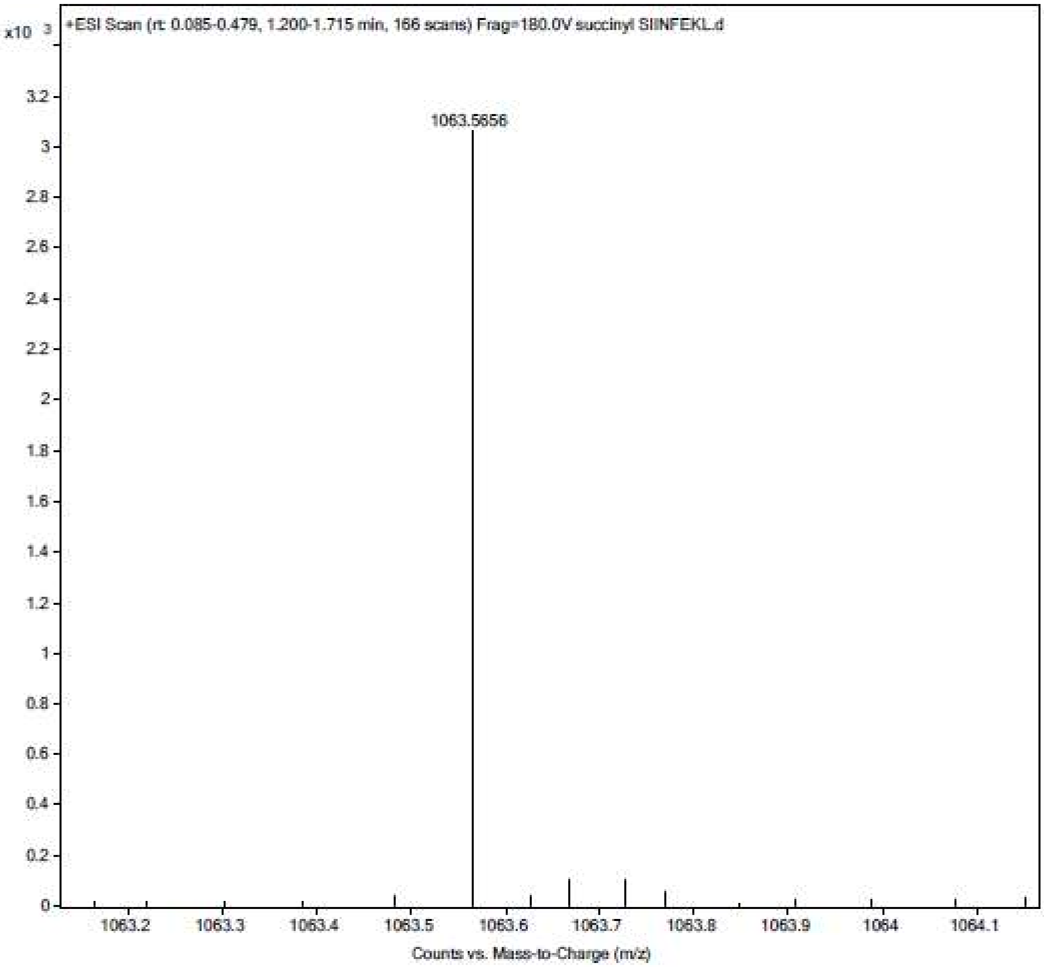

Scheme S6. Synthesis of Acetyl Lysine SIINFKEL

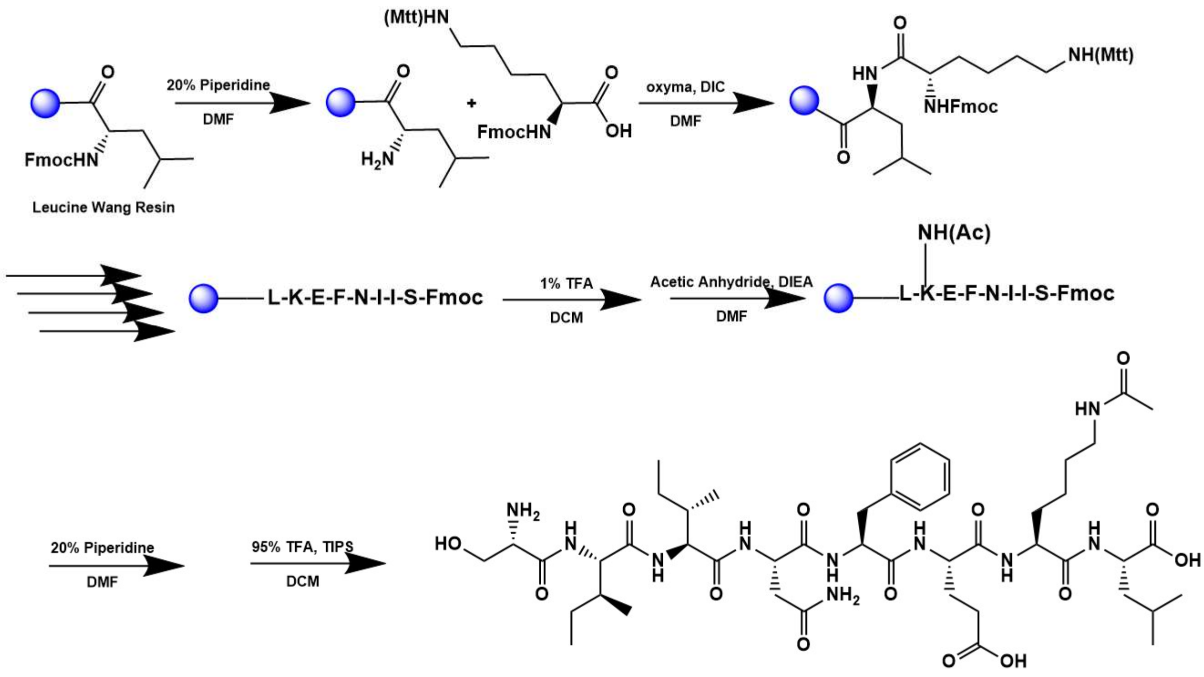

A 25 mL vessel of CEM discover bio manual peptide synthesizer was charged with 0.25 mmol of leucine wang resin. The Fmoc group was removed by using a 20% piperidine solution in DMF (10 mL). Using Synergy software, the deprotection protocol was run. The piperidine solution was drained and the resin was washed with DMF (4 x 10 mL). Fmoc-L-lysine(Mtt)-OH (5 eq, 1.25 mM) along with Oxyma (5 eq, 1.25 mM) and DIC (5 eq, 1.35 mmol) in DMF was added to the reaction vessel and the coupling protocol was run. The amino acid solution was drained, and the resin was washed with DMF (2 x 10 mL). The fmoc removal and coupling procedure was repeated as before using the same equivalencies for the remaining amino acids. The Mtt protecting group of lysine was removed by adding 10 mL of a TFA cocktail solution (1% TFA, 2% TIPS in DCM) to the resin and agitating for 10 minutes. The solution was drained, and this procedure was repeated five additional times. The resin was transferred to a 25 mL synthetic vessel and the lysine side chain of the peptide was acetylated agitating the resin for 1 hour in a solution of 5% acetic anhydride (0.5 mL), 8.5% DIEA (0.85 mL), and 86.5% DMF (8.65 mL). To remove the peptide from resin, a TFA cocktail solution (95% TFA, 2.5% TIPS, and 2.5% DCM) was added to the resin and agitated for 2 hours. The resin was filtered, and the resulting solution was concentrated in vacuo. The peptide was trituated with cold diethyl ether and purified using reverse phase HPLC using H_2O/CH_3CN. The sample was analyzed for purity using a Waters 1525 Binary HPLC Pump using a Phenomenex Luna 5u C8(2) 100A (250 x 4.60 mm) column; gradient eluted with H_20/CH_3CN. Molecular weight was confirmed using high resolution electrospray ionization mass spectrometry (HRMS, ESI/MS) analyses obtained on an Agilent 6545B Q-TOF LC/MS equipped with 1260 infinity II LC system with auto sampler. The final peptide product was lyophilized and stored at -20°C until further use.

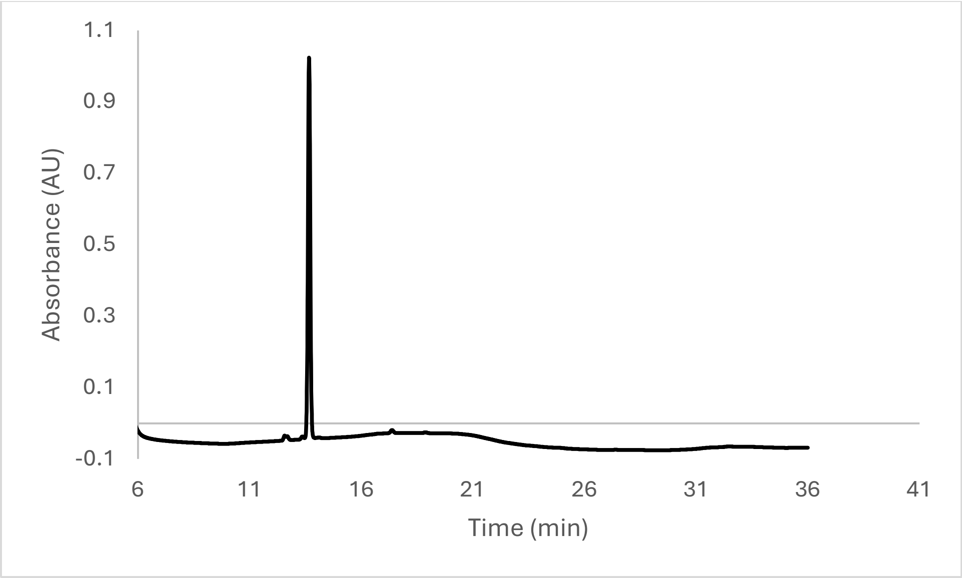

ESI-MS calculated [M+H+]: 1005.5620, found 1005.5586.

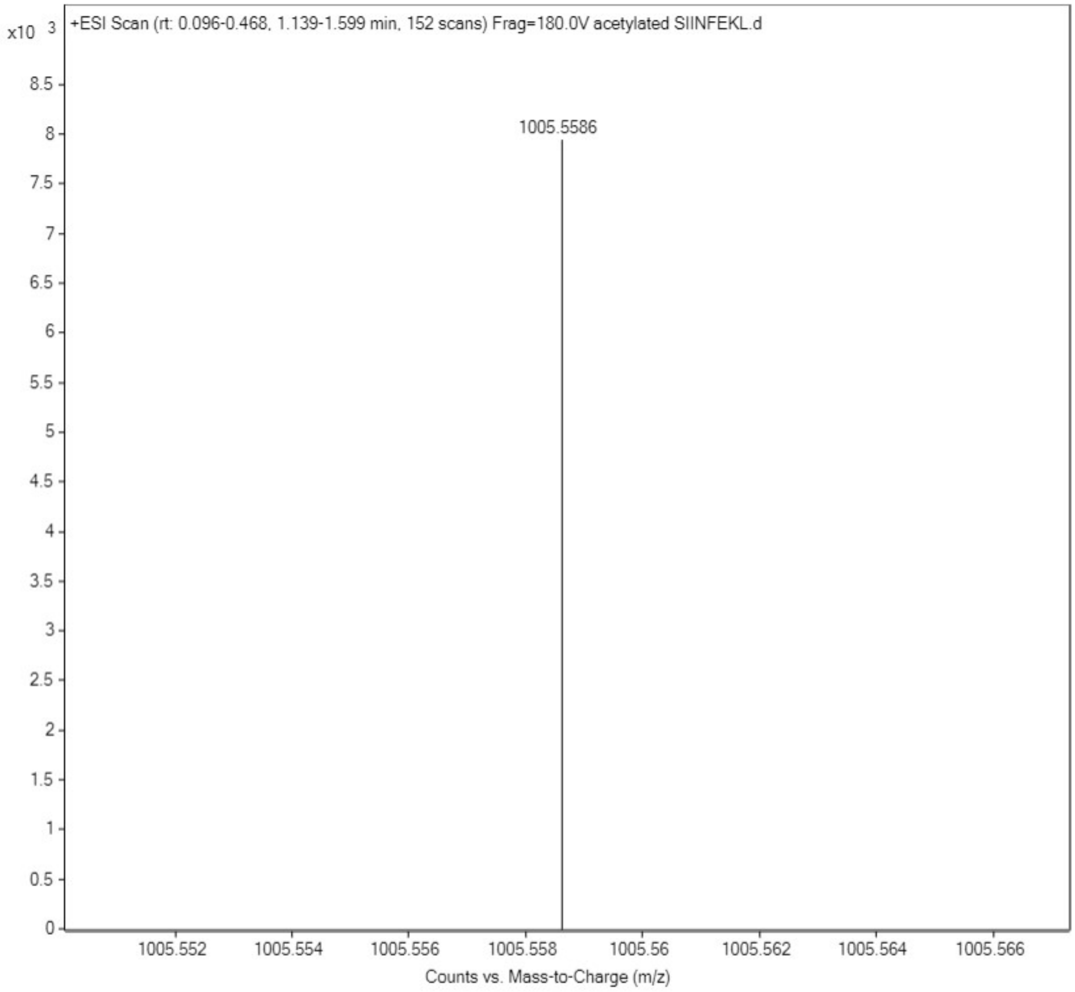

Scheme S7. Synthesis of Biotinylated Lysine SIINFKEL

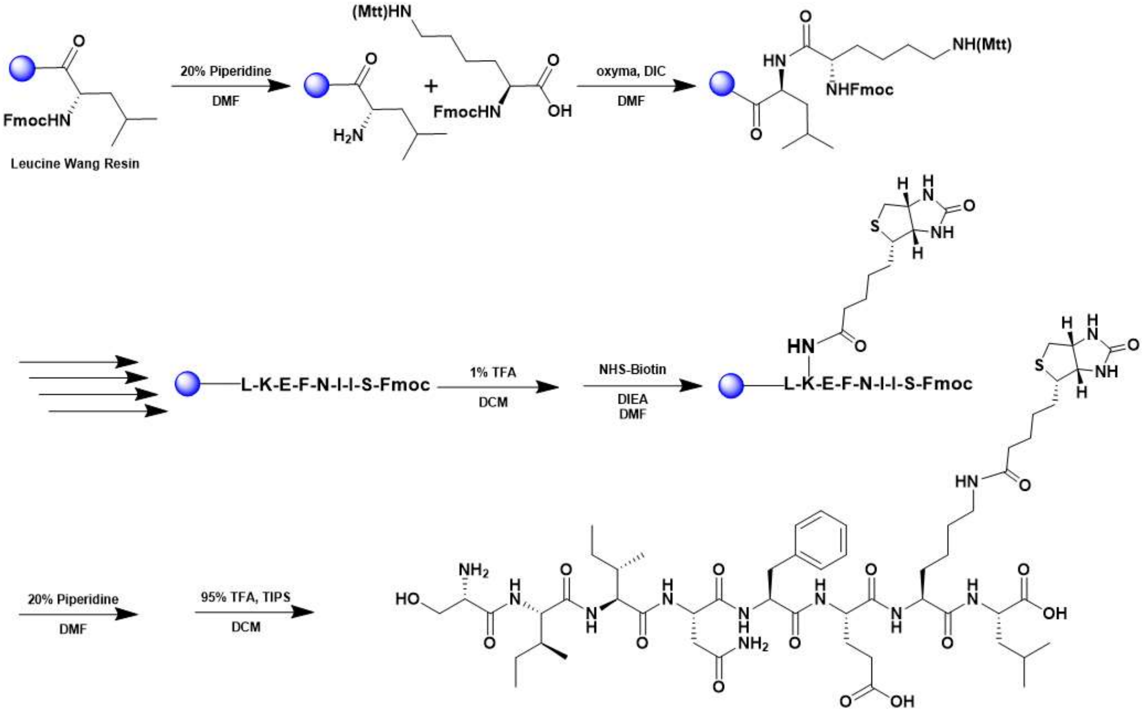

A 25 mL vessel of CEM discover bio manual peptide synthesizer was charged with 0.25 mmol of leucine wang resin. The Fmoc group was removed by using a 20% piperidine solution in DMF (10 mL). Using Synergy software, the deprotection protocol was run. The piperidine solution was drained and the resin was washed with DMF (4 x 10 mL). Fmoc-L-lysine(Mtt)-OH (5 eq, 1.25 mM) along with Oxyma (5 eq, 1.25 mM) and DIC (5 eq, 1.35 mmol) in DMF was added to the reaction vessel and the coupling protocol was run. The amino acid solution was drained, and the resin was washed with DMF (2 x 10 mL). The fmoc removal and coupling procedure was repeated as before using the same equivalencies for the remaining amino acids. The Mtt protecting group of lysine was removed by adding 10 mL of a TFA cocktail solution (1% TFA, 2% TIPS in DCM) to the resin and agitating for 10 minutes. The solution was drained, and this procedure was repeated five additional times. Biotin N-hydroxy succinimide ester (10 eq, 2.5 mM) and DIEA (4 eq, 1 mM) in DMF were reacted with peptide for 2 h while agitated. To remove the peptide from resin, a TFA cocktail solution (95% TFA, 2.5% TIPS, and 2.5% DCM) was added to the resin and agitated for 2 hours. The resin was filtered, and the resulting solution was concentrated in vacuo. The peptide was trituated with cold diethyl ether and purified using reverse phase HPLC using H_2O/CH_3CN. The sample was analyzed for purity using a Waters 1525 Binary HPLC Pump using a Phenomenex Luna 5u C8(2) 100A (250 x 4.60 mm) column; gradient eluted with H_20/CH_3CN. Molecular weight was confirmed using high resolution electrospray ionization mass spectrometry (HRMS, ESI/MS) analyses obtained on an Agilent 6545B Q-TOF LC/MS equipped with 1260 infinity II LC system with auto sampler. The final peptide product was lyophilized and stored at -20°C until further use.

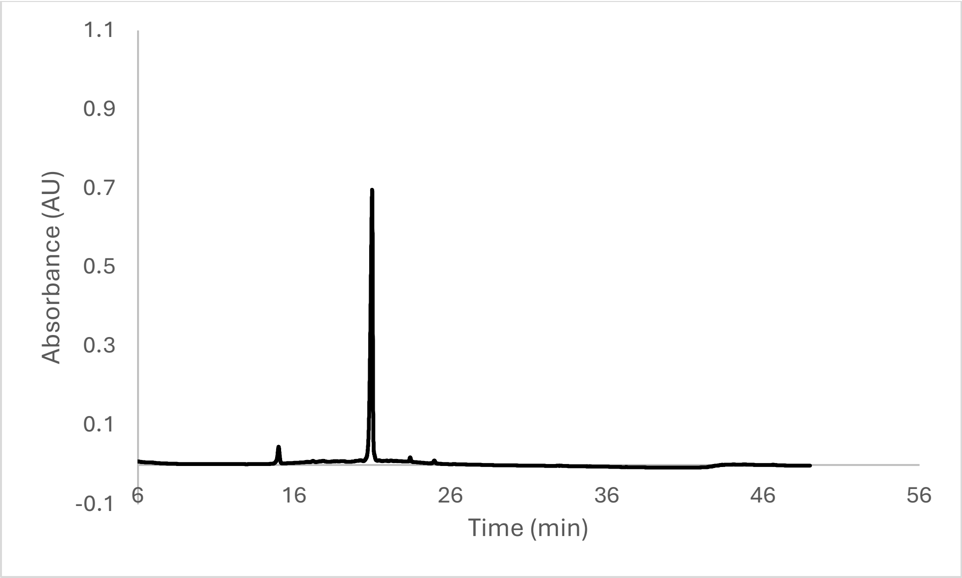

ESI-MS calculated [M+H$^+$]: 1189.6291, found 1189.6272.

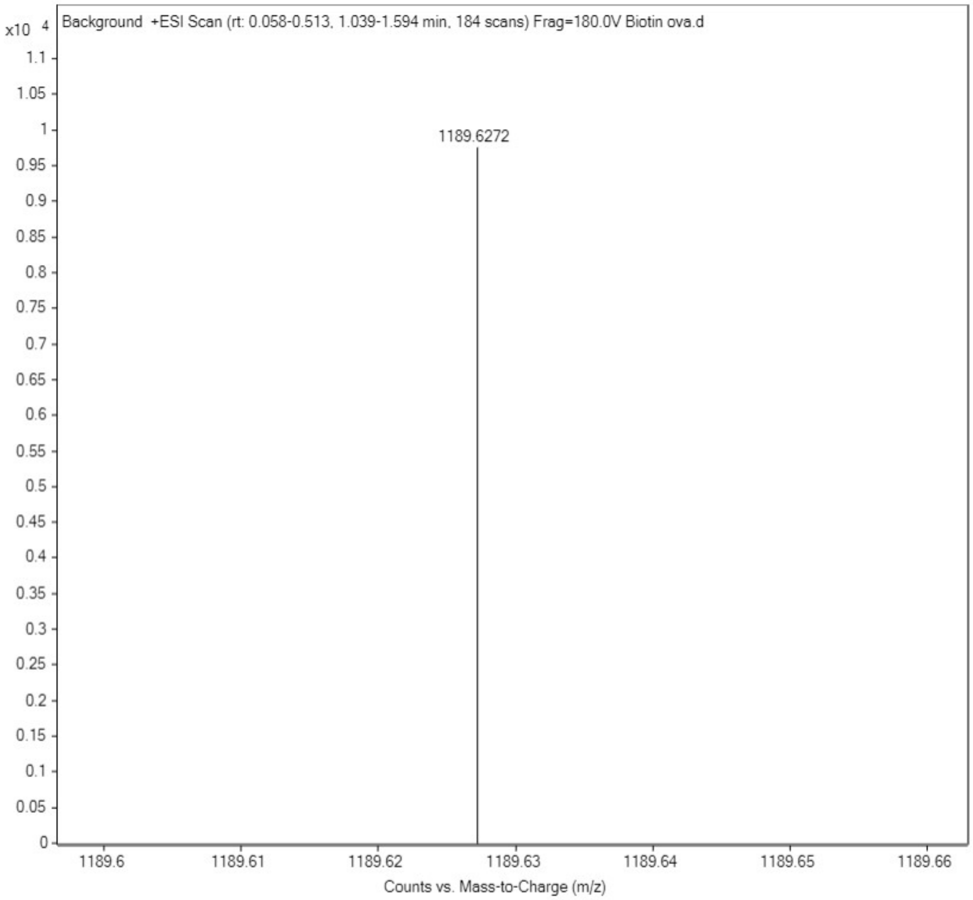

Scheme S8. Synthesis of Phosphoserine SIINFKEL

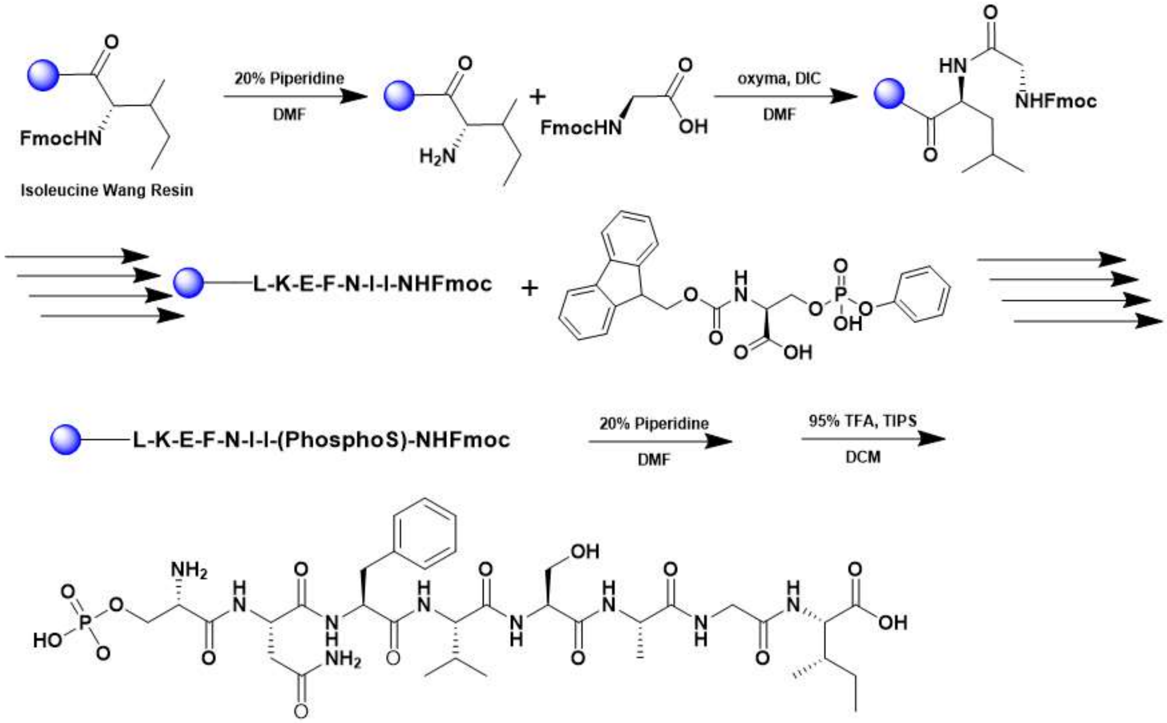

A 25 mL vessel of CEM discover bio manual peptide synthesizer was charged with 0.25 mmol of leucine wang resin. The Fmoc group was removed by using a 20% piperidine solution in DMF (10 mL). Using Synergy software, the deprotection protocol was run. The piperidine solution was drained and the resin was washed with DMF (4 x 10 mL). Fmoc-L-lysine(Mtt)-OH (5 eq, 1.25 mM) along with Oxyma (5 eq, 1.25 mM) and DIC (5 eq, 1.35 mmol) in DMF was added to the reaction vessel and the coupling protocol was run. The amino acid solution was drained, and the resin was washed with DMF (2 x 10 mL). The fmoc removal and coupling procedure was repeated as before using the same equivalencies for the remaining amino acids. To remove the peptide from resin, a TFA cocktail solution (95% TFA, 2.5% TIPS, and 2.5% DCM) was added to the resin and agitated for 2 hours. The resin was filtered, and the resulting solution was concentrated in vacuo. The peptide was trituated with cold diethyl ether and purified using reverse phase HPLC using H_2O/CH_3CN. The sample was analyzed for purity using a Waters 1525 Binary HPLC Pump using a Phenomenex Luna 5u C8(2) 100A (250 x 4.60 mm) column; gradient eluted with H_20/CH_3CN. Molecular weight was confirmed using high resolution electrospray ionization mass spectrometry (HRMS, ESI/MS) analyses obtained on an Agilent 6545B Q-TOF LC/MS equipped with 1260 infinity II LC system with auto sampler. The final peptide product was lyophilized and stored at -20°C until further use.

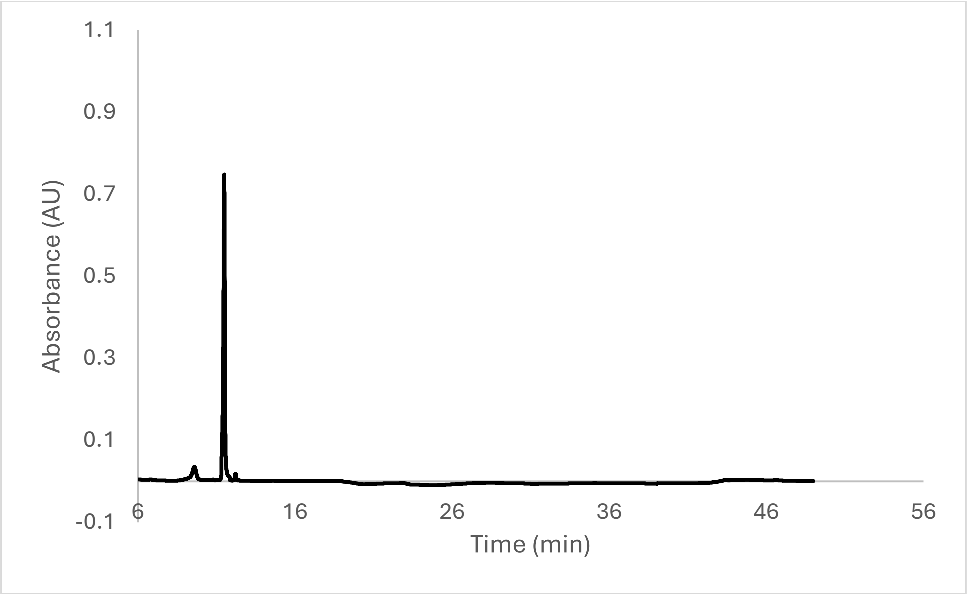

ESI-MS calculated [M+H$^+$]: 1042.5100, found 1042.5072.

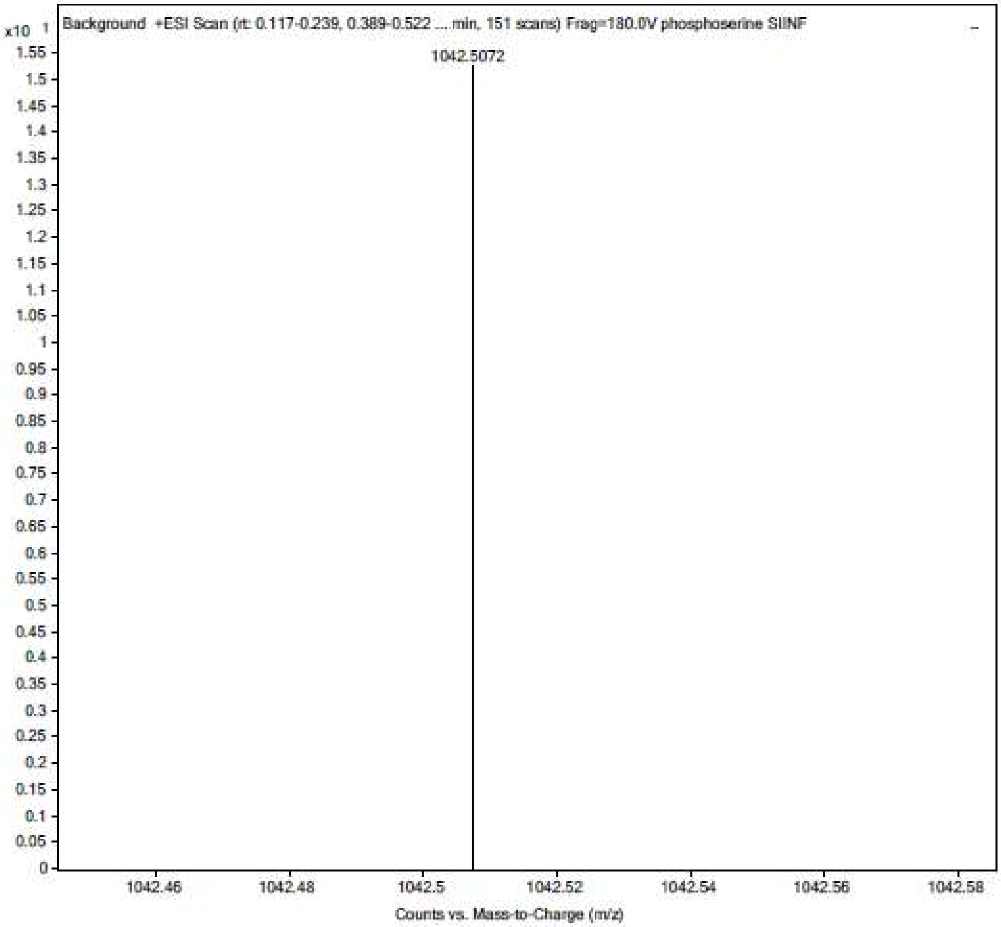

Scheme S9. Synthesis of SNFVSAGI

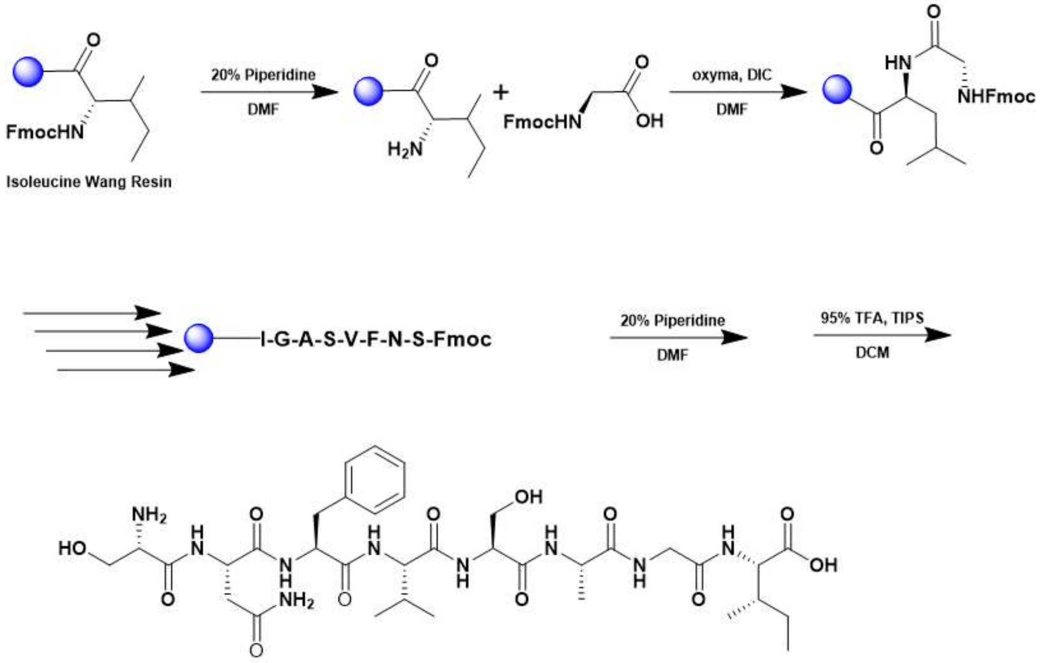

A 25 mL vessel of CEM discover bio manual peptide synthesizer was charged with 0.25 mmol of isoleucine wang resin. The Fmoc group was removed by using a 20% piperidine solution in DMF (10 mL). Using Synergy software, the deprotection protocol was run. The piperidine solution was drained and the resin was washed with DMF (4 x 10 mL). Fmoc-L-glycine (5 eq, 1.25 mM) along with Oxyma (5 eq, 1.25 mM) and DIC (5 eq, 1.35 mmol) in DMF was added to the reaction vessel and the coupling protocol was run. The amino acid solution was drained, and the resin was washed with DMF (2 x 10 mL). The fmoc removal and coupling procedure was repeated as before using the same equivalencies for the remaining amino acids. To remove the peptide from resin, a TFA cocktail solution (95% TFA, 2.5% TIPS, and 2.5% DCM) was added to the resin and agitated for 2 hours. The resin was filtered, and the resulting solution was concentrated in vacuo. The peptide was trituated with cold diethyl ether and purified using reverse phase HPLC using H_2O/CH_3CN. The sample was analyzed for purity using a Waters 1525 Binary HPLC Pump using a Phenomenex Luna 5u C8(2) 100A (250 x 4.60 mm) column; gradient eluted with H_20/CH_3CN. Molecular weight was confirmed using high resolution electrospray ionization mass spectrometry (HRMS, ESI/MS) analyses obtained on an Agilent 6545B Q-TOF LC/MS equipped with 1260 infinity II LC system with auto sampler. The final peptide product was lyophilized and stored at -20°C until further use.

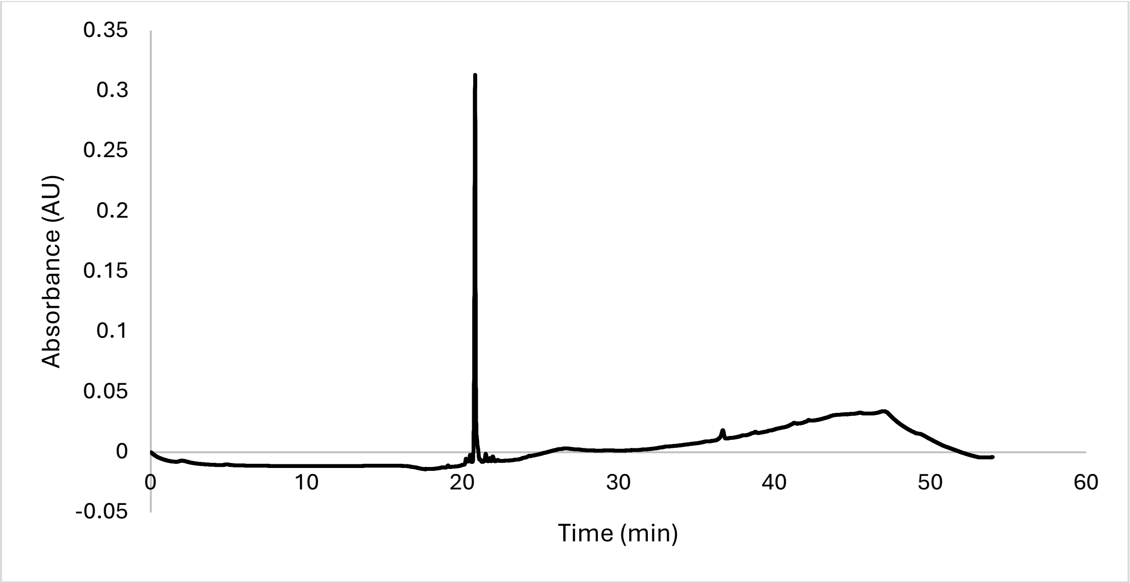

ESI-MS calculated [M+H$^+$]: 794.4048, found 794.4038

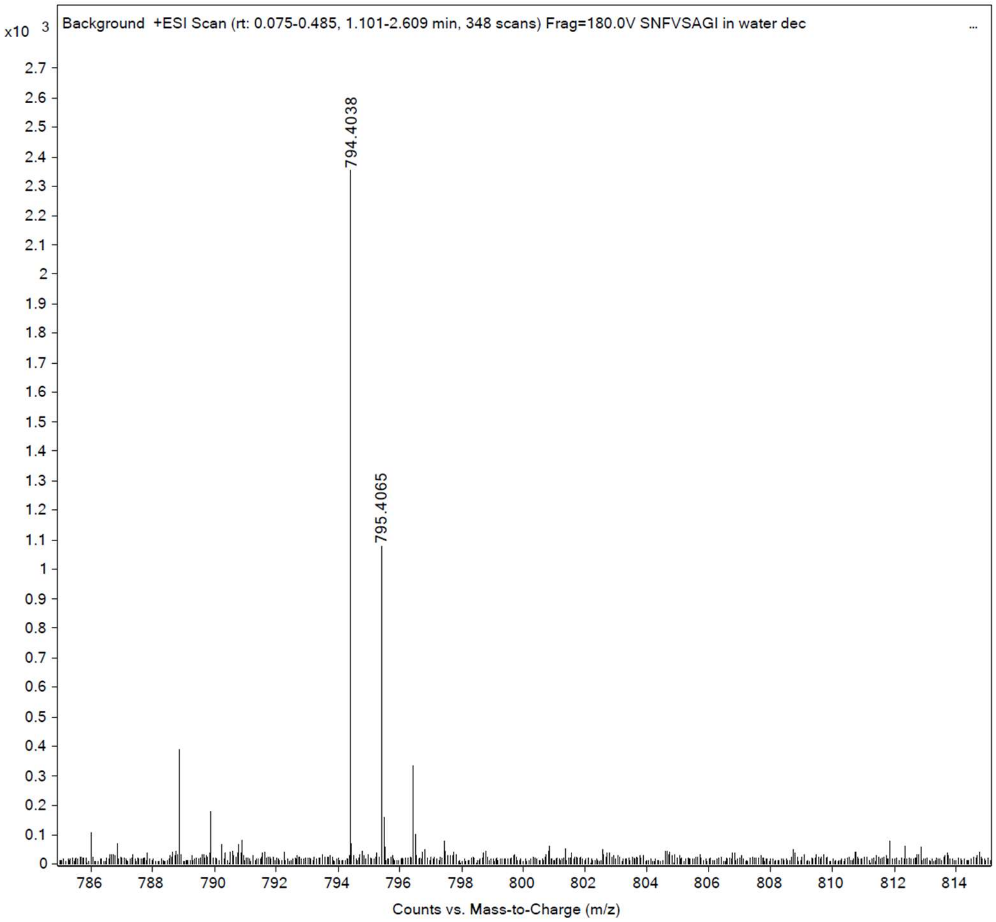

Scheme S10. Synthesis of ESIVRFPNI

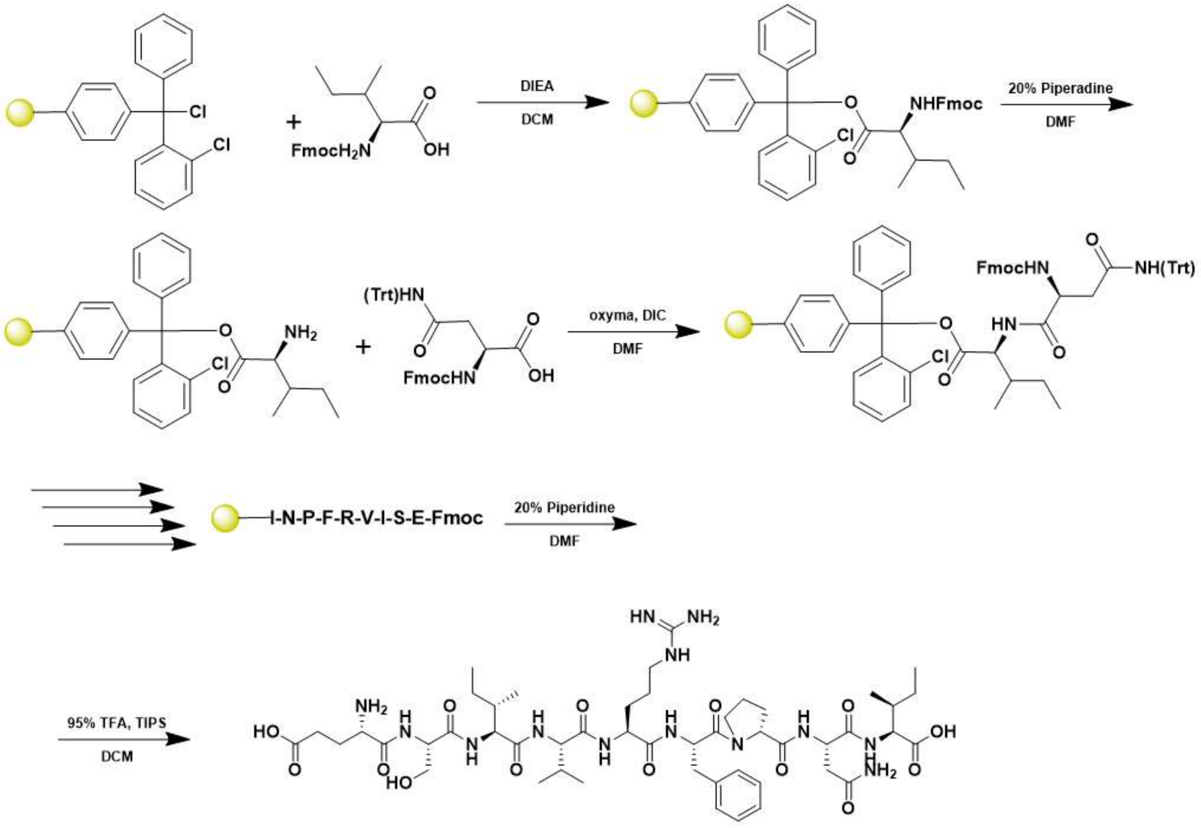

A 25 mL peptide synthesis vessel charged with 2-hlorotrityl chloride resin (0.25mmol) was added Fmoc-L-isoleucine-OH (1.1 eq, 0.275 mmol) and DIEA (3 eq, 0.75 mmol) in dry DCM. The resin was agitated for 1 h at ambient temperature and washed with MeOH and DCM (3 x each). The Fmoc group was removed by using a 20% piperidine solution in DMF for 30 min at ambient temperature, then washed as before. Fmoc-L-asparagine(Trt)-OH (5 eq, 1.25 mM) along with Oxyma (5 eq, 1.25 mM) and DIC (5 eq, 1.35 mmol) in DMF was added to the reaction vessel and agitated for 2 h at ambient temperature. The Fmoc removal and coupling procedure was repeated as before using the same equivalencies for the remaining amino acids. To remove the peptide from resin, a TFA cocktail solution (95% TFA, 2.5% TIPS, and 2.5% DCM) was added to the resin and agitated for 2 hours. The resin was filtered, and the resulting solution was concentrated in vacuo. The peptide was triturated with cold diethyl ether and purified using reverse phase HPLC using H_2O/CH_3CN. The sample was analyzed for purity using a Waters 1525 Binary HPLC Pump using a Phenomenex Luna 5u C8(2) 100A (250 x 4.60 mm) column; gradient eluted with H_20/CH_3CN. Molecular weight was confirmed using high resolution electrospray ionization mass spectrometry (HRMS, ESI/MS) analyses obtained on an Agilent 6545B Q-TOF LC/MS equipped with 1260 infinity II LC system with auto sampler. The final peptide product was lyophilized and stored at -20°C until further use.

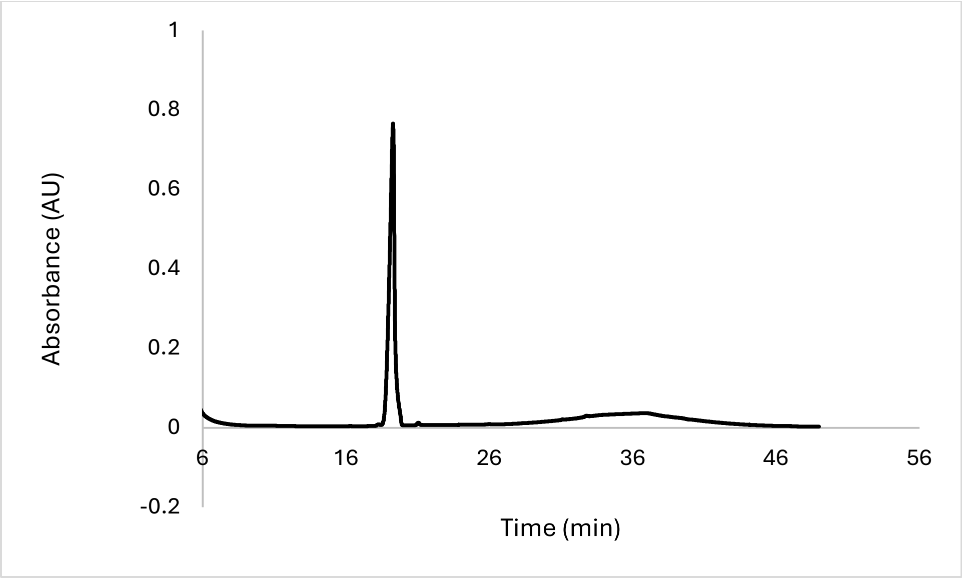

ESI-MS calculated [M]: 1073.5869, found 1073.5870

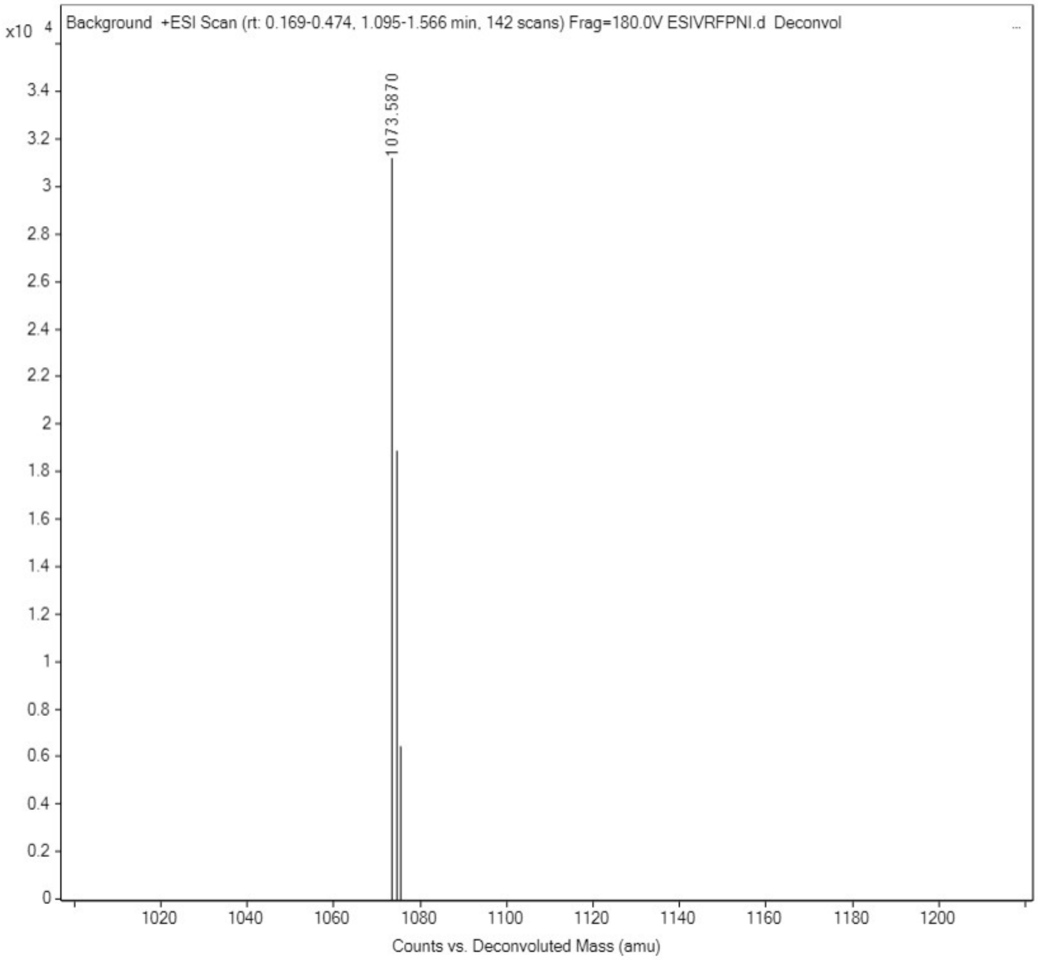

Scheme S11. Synthesis of N-Acetyl ESIVRFPNI

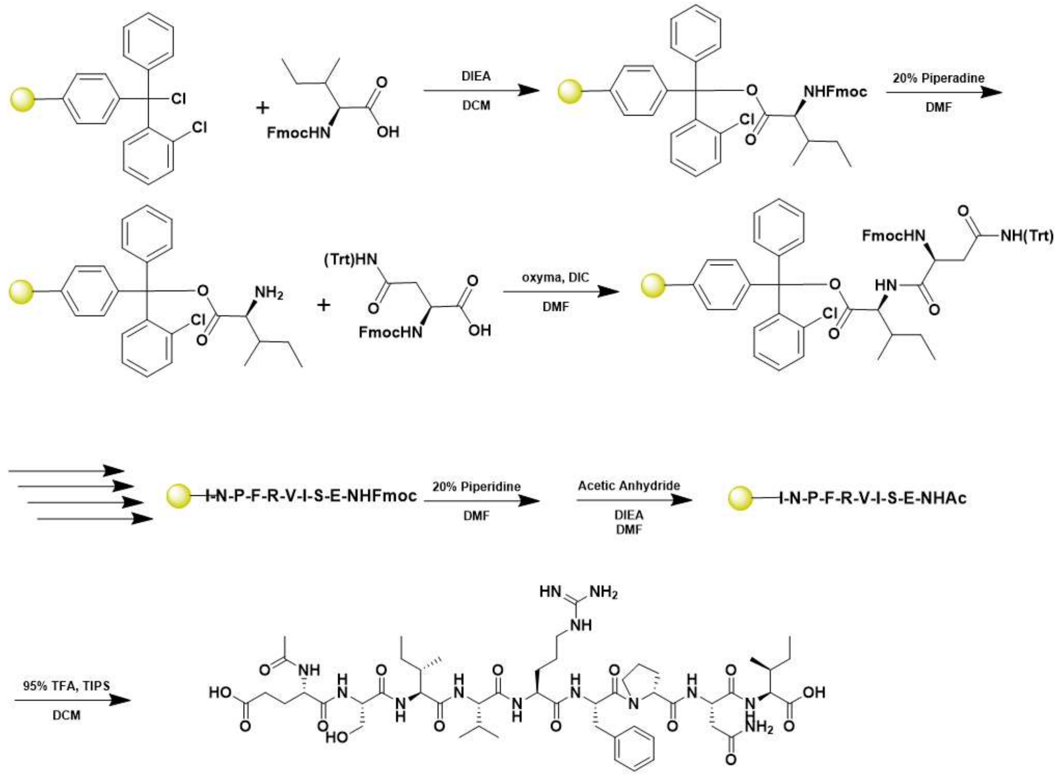

A 25 mL peptide synthesis vessel charged with 2-hlorotrityl chloride resin (0.25mmol) was added Fmoc-L-isoleucine-OH (1.1 eq, 0.275 mmol) and DIEA (3 eq, 0.75 mmol) in dry DCM. The resin was agitated for 1 h at ambient temperature and washed with MeOH and DCM (3 x each). The Fmoc group was removed by using a 20% piperidine solution in DMF for 30 min at ambient temperature, then washed as before. Fmoc-L-asparagine(Trt)-OH (5 eq, 1.25 mM) along with Oxyma (5 eq, 1.25 mM) and DIC (5 eq, 1.35 mmol) in DMF was added to the reaction vessel and agitated for 2 h at ambient temperature. The Fmoc removal and coupling procedure was repeated as before using the same equivalencies for the remaining amino acids. The final amino acid was Fmoc deprotected as described before and was acetylated by agitating the resin for 1 hour in a solution of 5% acetic anhydride (0.5 mL), 8.5% DIEA (0.85 mL), and 86.5% DMF (8.65 mL). To remove the peptide from resin, a TFA cocktail solution (95% TFA, 2.5% TIPS, and 2.5% DCM) was added to the resin and agitated for 2 hours. The resin was filtered, and the resulting solution was concentrated in vacuo. The peptide was triturated with cold diethyl ether and purified using reverse phase HPLC using H_2O/CH_3CN. The sample was analyzed for purity using a Waters 1525 Binary HPLC Pump using a Phenomenex Luna 5u C8(2) 100A (250 x 4.60 mm) column; gradient eluted with H_20/CH_3CN. Molecular weight was confirmed using high resolution electrospray ionization mass spectrometry (HRMS, ESI/MS) analyses obtained on an Agilent 6545B Q-TOF LC/MS equipped with 1260 infinity II LC system with auto sampler. The final peptide product was lyophilized and stored at -20°C until further use.

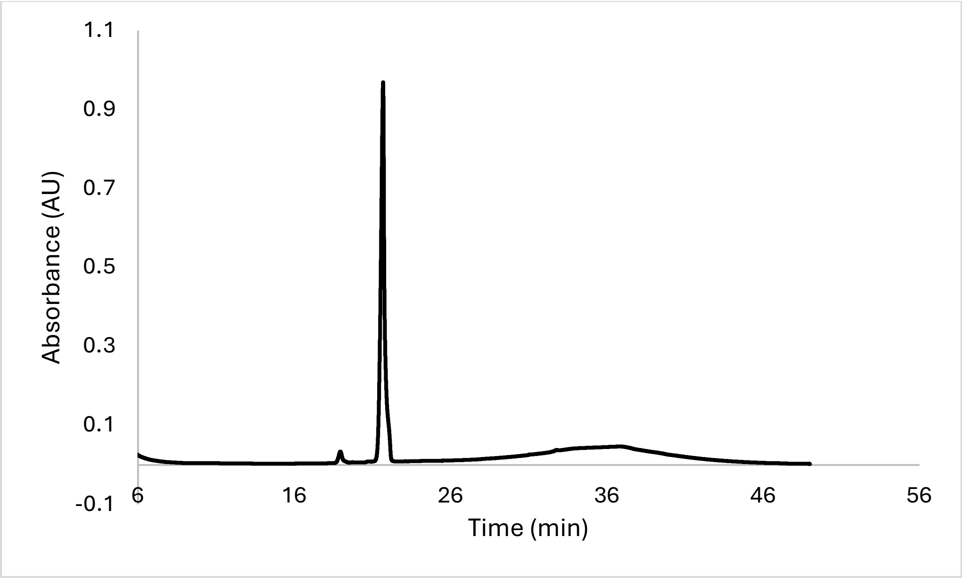

ESI-MS calculated [M]: 1116.6048, found 1116.6039

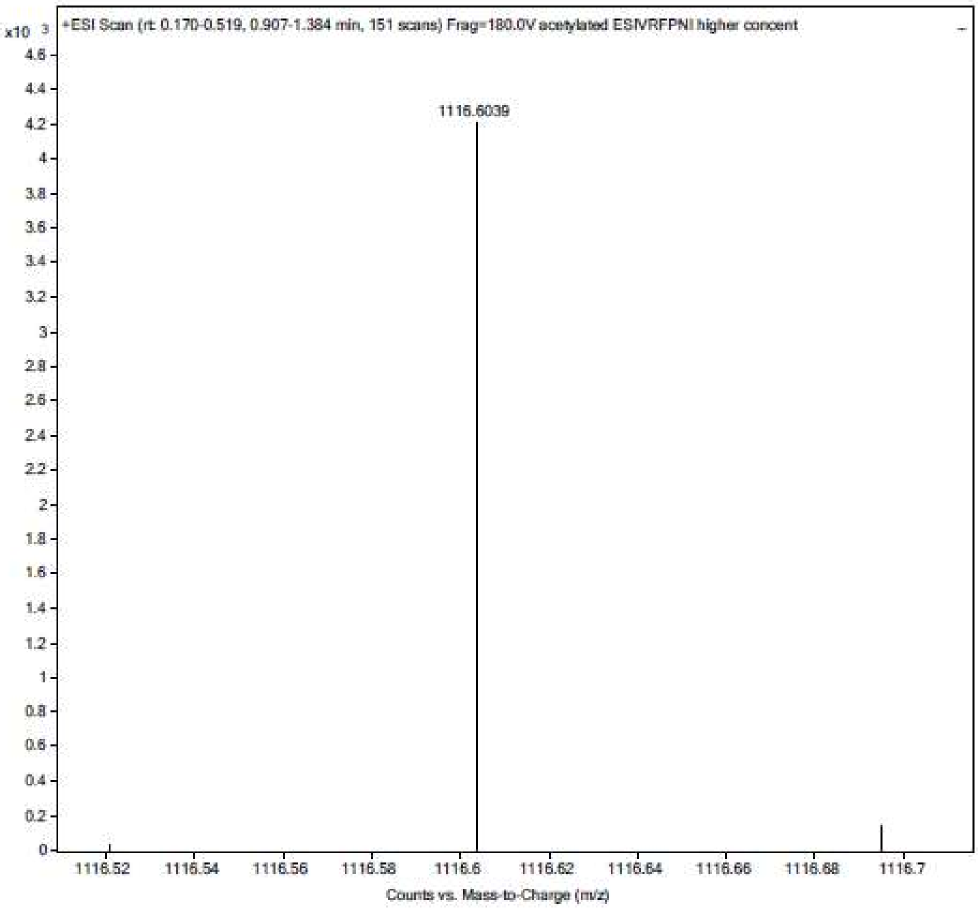

Scheme S12. Synthesis of Citrullinated ESIVRFPNI

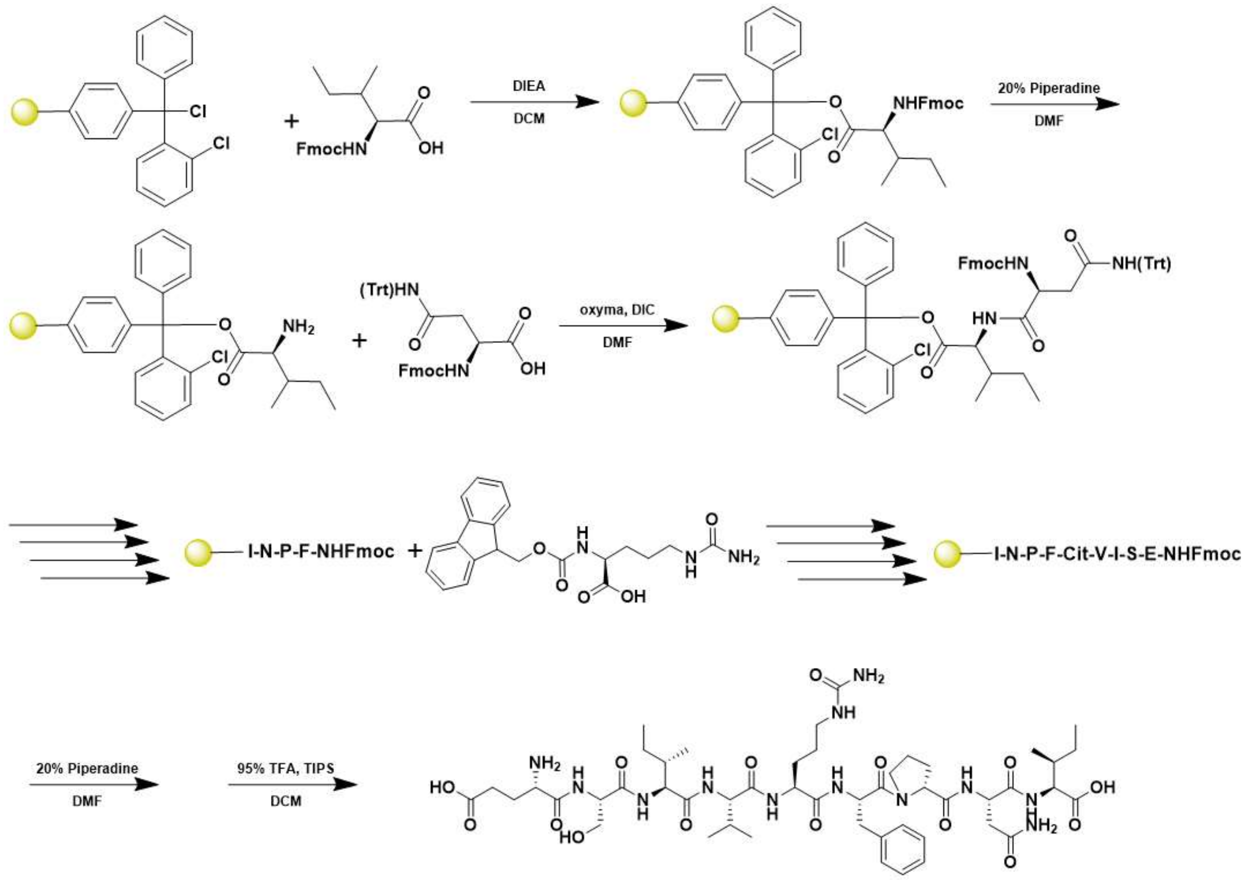

A 25 mL peptide synthesis vessel charged with 2-hlorotrityl chloride resin (0.25mmol) was added Fmoc-L-isoleucine-OH (1.1 eq, 0.275 mmol) and DIEA (3 eq, 0.75 mmol) in dry DCM. The resin was agitated for 1 h at ambient temperature and washed with MeOH and DCM (3 x each). The Fmoc group was removed by using a 20% piperidine solution in DMF for 30 min at ambient temperature, then washed as before. Fmoc-L-asparagine(Trt)-OH (5 eq, 1.25 mM) along with Oxyma (5 eq, 1.25 mM) and DIC (5 eq, 1.35 mmol) in DMF was added to the reaction vessel and agitated for 2 h at ambient temperature. The Fmoc removal and coupling procedure was repeated as before using the same equivalencies for the remaining amino acids. For the 5th position of the amino acid, Fmoc was deprotected as described above and Fmoc-L-citrulline (5 eq, 1.25 mM) along with Oxyma (5 eq, 1.25 mM) and DIC (5 eq, 1.35 mmol) in DMF was added to the reaction vessel. To remove the peptide from resin, a TFA cocktail solution (95% TFA, 2.5% TIPS, and 2.5% DCM) was added to the resin and agitated for 2 hours. The resin was filtered, and the resulting solution was concentrated in vacuo. The peptide was trituated with cold diethyl ether and purified using reverse phase HPLC using H_2O/CH_3CN. The sample was analyzed for purity using a Waters 1525 Binary HPLC Pump using a Phenomenex Luna 5u C8(2) 100A (250 x 4.60 mm) column; gradient eluted with H_20/CH_3CN. Molecular weight was confirmed using high resolution electrospray ionization mass spectrometry (HRMS, ESI/MS) analyses obtained on an Agilent 6545B Q-TOF LC/MS equipped with 1260 infinity II LC system with auto sampler. The final peptide product was lyophilized and stored at -20°C until further use.

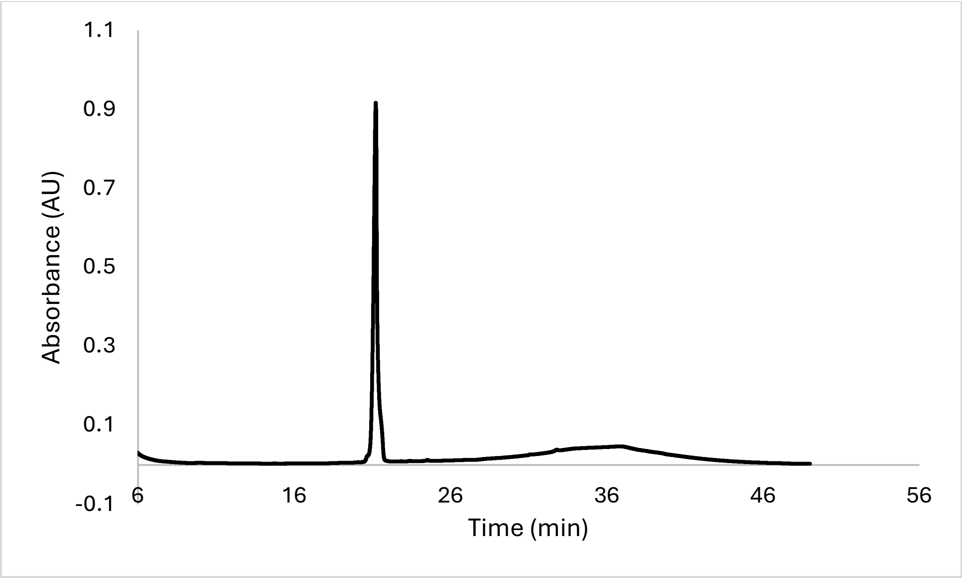

ESI-MS calculated [M]: 1074.5710, found 1074.5708

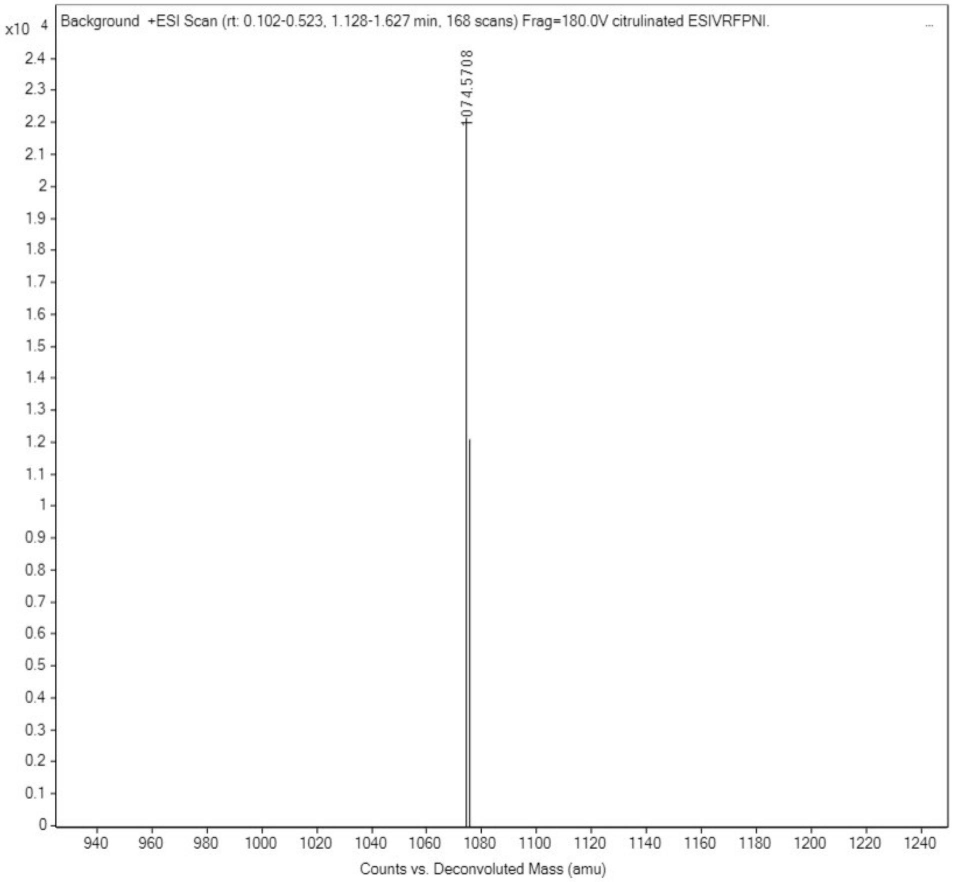

Scheme S13. Synthesis of Hydroxy Proline ESIVRFPNI

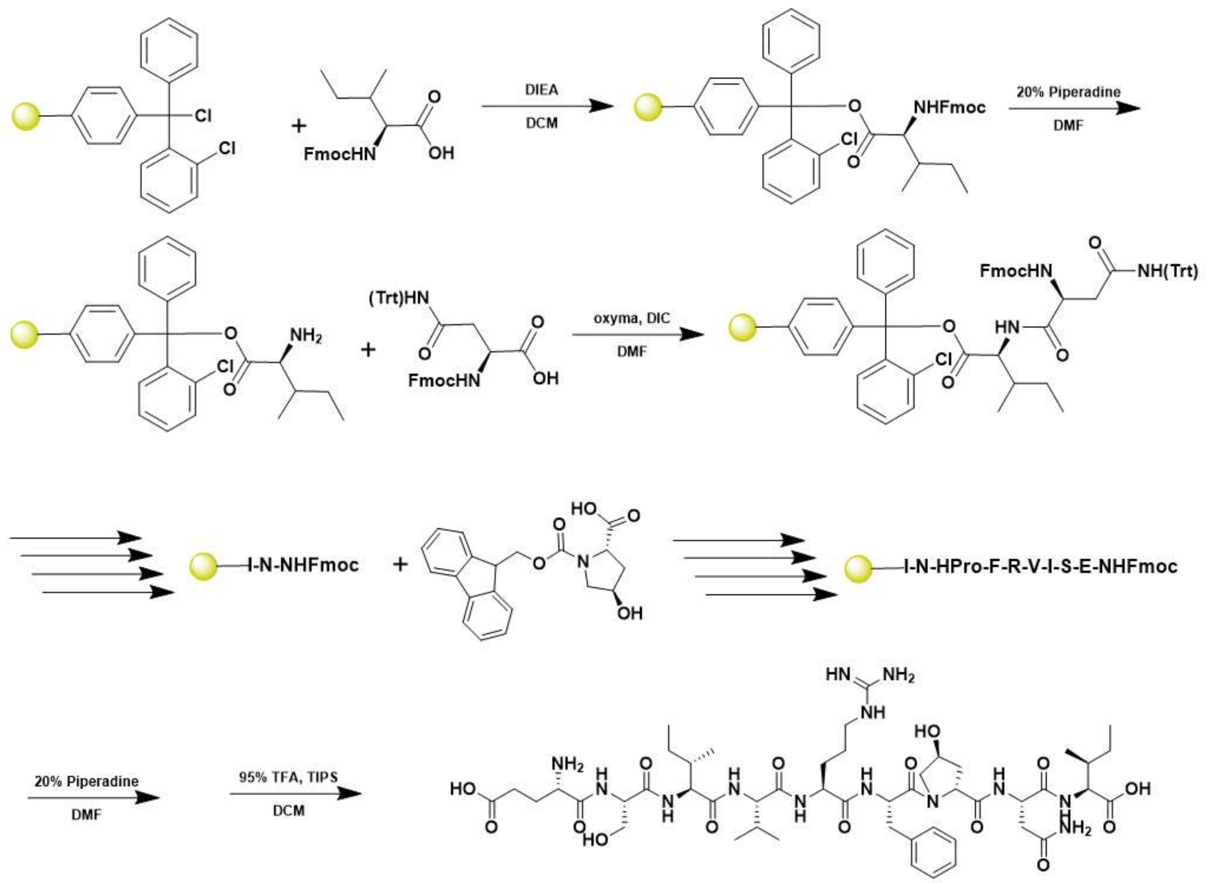

A 25 mL peptide synthesis vessel charged with 2-hlorotrityl chloride resin (0.25mmol) was added Fmoc-L-isoleucine-OH (1.1 eq, 0.275 mmol) and DIEA (3 eq, 0.75 mmol) in dry DCM. The resin was agitated for 1 h at ambient temperature and washed with MeOH and DCM (3 x each). The Fmoc group was removed by using a 20% piperidine solution in DMF for 30 min at ambient temperature, then washed as before. Fmoc-L-asparagine(Trt)-OH (5 eq, 1.25 mM) along with Oxyma (5 eq, 1.25 mM) and DIC (5 eq, 1.35 mmol) in DMF was added to the reaction vessel and agitated for 2 h at ambient temperature. The Fmoc removal and coupling procedure was repeated as before using the same equivalencies for the remaining amino acids. For the 6th position of the amino acid, Fmoc was deprotected as described above and Fmoc-L-trans-4-hydroxyproline (5 eq, 1.25 mM) was added along with Oxyma (5 eq, 1.25 mM) and DIC (5 eq, 1.35 mmol) in DMF the reaction vessel. To remove the peptide from resin, a TFA cocktail solution (95% TFA, 2.5% TIPS, and 2.5% DCM) was added to the resin and agitated for 2 hours. The resin was filtered, and the resulting solution was concentrated in vacuo. The peptide was trituated with cold diethyl ether and purified using reverse phase HPLC using H_2O/CH_3CN. The sample was analyzed for purity using a Waters 1525 Binary HPLC Pump using a Phenomenex Luna 5u C8(2) 100A (250 x 4.60 mm) column; gradient eluted with H_20/CH_3CN. Molecular weight was confirmed using high resolution electrospray ionization mass spectrometry (HRMS, ESI/MS) analyses obtained on an Agilent 6545B Q-TOF LC/MS equipped with 1260 infinity II LC system with auto sampler. The final peptide product was lyophilized and stored at -20°C until further use.

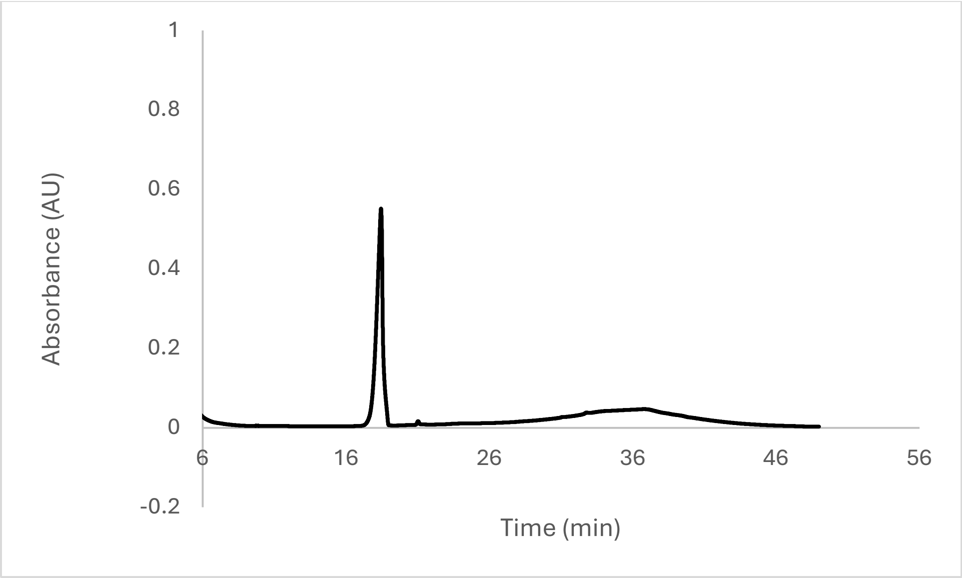

ESI-MS calculated [M]: 1089.5819, found 1089.5838

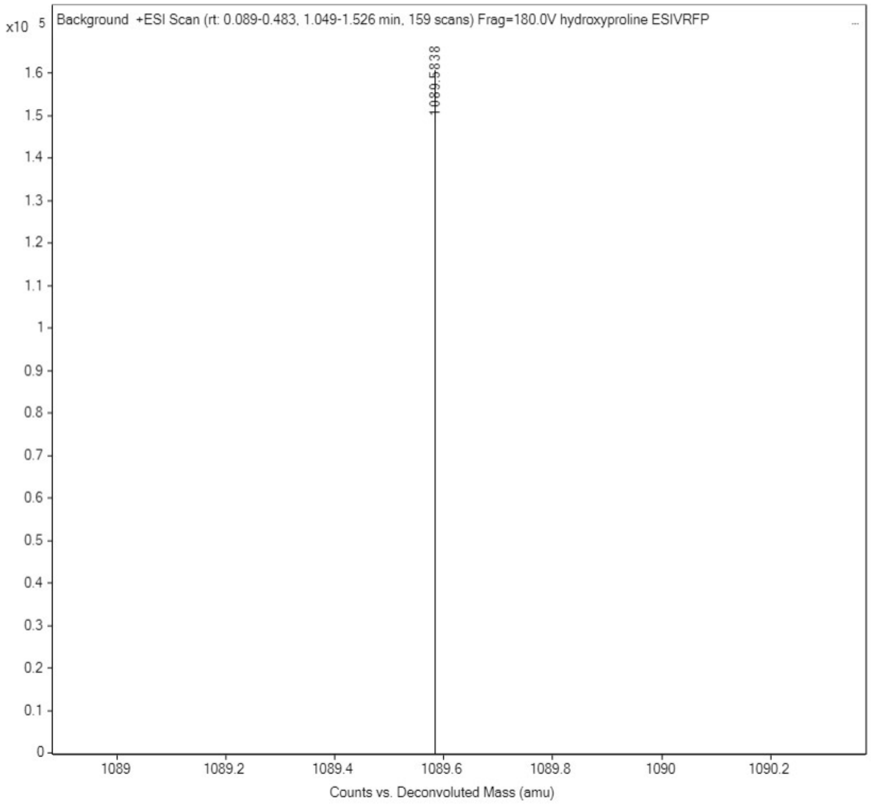

Scheme S14. Synthesis of YNVRKSEM

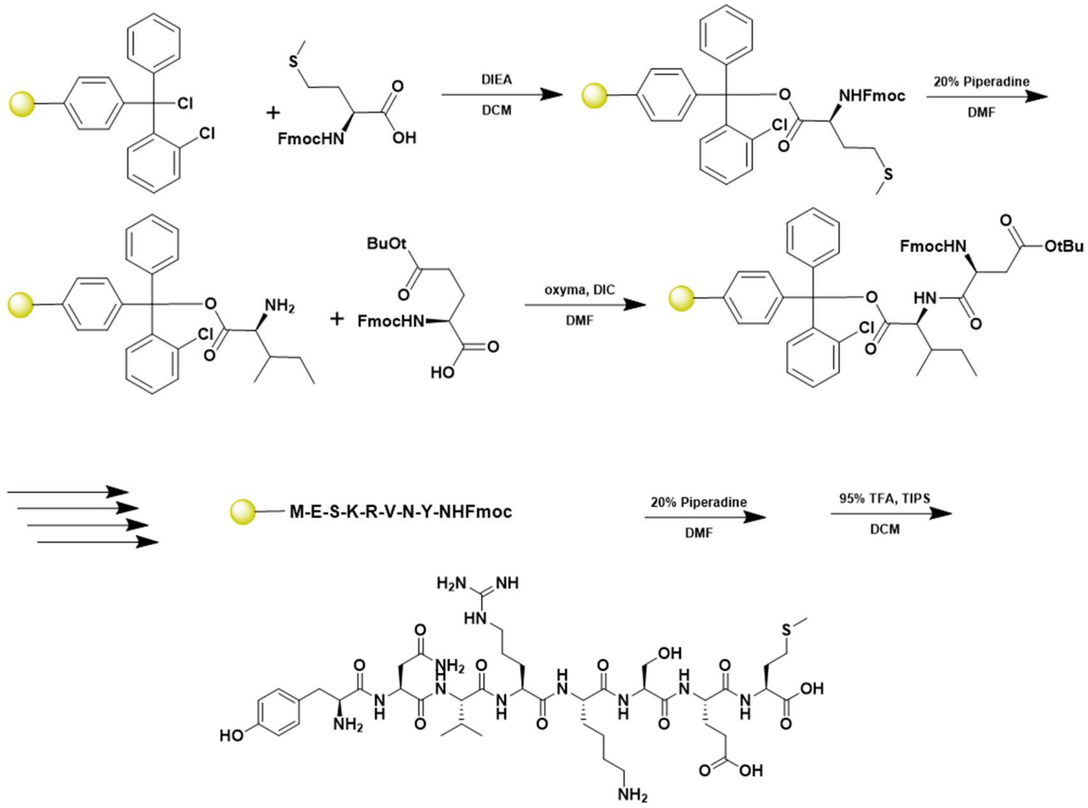

A 25 mL peptide synthesis vessel charged with 2-hlorotrityl chloride resin (0.25mmol) was added Fmoc-L-methionine-OH (1.1 eq, 0.275 mmol) and DIEA (3 eq, 0.75 mmol) in dry DCM. The resin was agitated for 1 h at ambient temperature and washed with MeOH and DCM (3 x each). The Fmoc group was removed by using a 20% piperidine solution in DMF for 30 min at ambient temperature, then washed as before. Fmoc-L-glutamate(OtBu)-OH (5 eq, 1.25 mM) along with Oxyma (5 eq, 1.25 mM) and DIC (5 eq, 1.35 mmol) in DMF was added to the reaction vessel and agitated for 2 h at ambient temperature. The Fmoc removal and coupling procedure was repeated as before using the same equivalencies for the remaining amino acids. To remove the peptide from resin, a TFA cocktail solution (95% TFA, 2.5% TIPS, and 2.5% DCM) was added to the resin and agitated for 2 hours. The resin was filtered, and the resulting solution was concentrated in vacuo. The peptide was triturated with cold diethyl ether and purified using reverse phase HPLC using H_2O/CH_3CN. The sample was analyzed for purity using a Waters 1525 Binary HPLC Pump using a Phenomenex Luna 5u C8(2) 100A (250 x 4.60 mm) column; gradient eluted with H_20/CH_3CN. Molecular weight was confirmed using high resolution electrospray ionization mass spectrometry (HRMS, ESI/MS) analyses obtained on an Agilent 6545B Q-TOF LC/MS equipped with 1260 infinity II LC system with auto sampler. The final peptide product was lyophilized and stored at -20°C until further use.

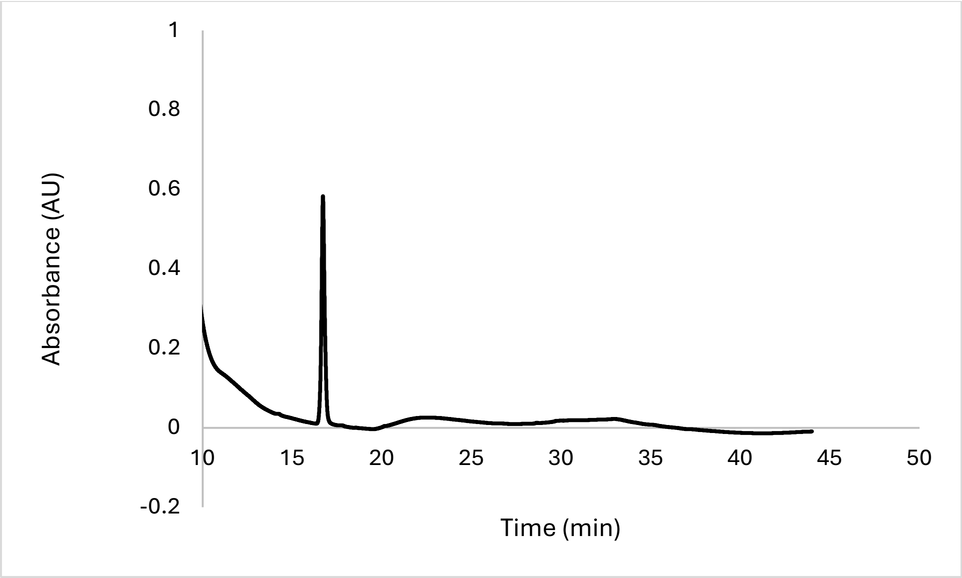

ESI-MS calculated [M]: 967.5715, found 967.5711

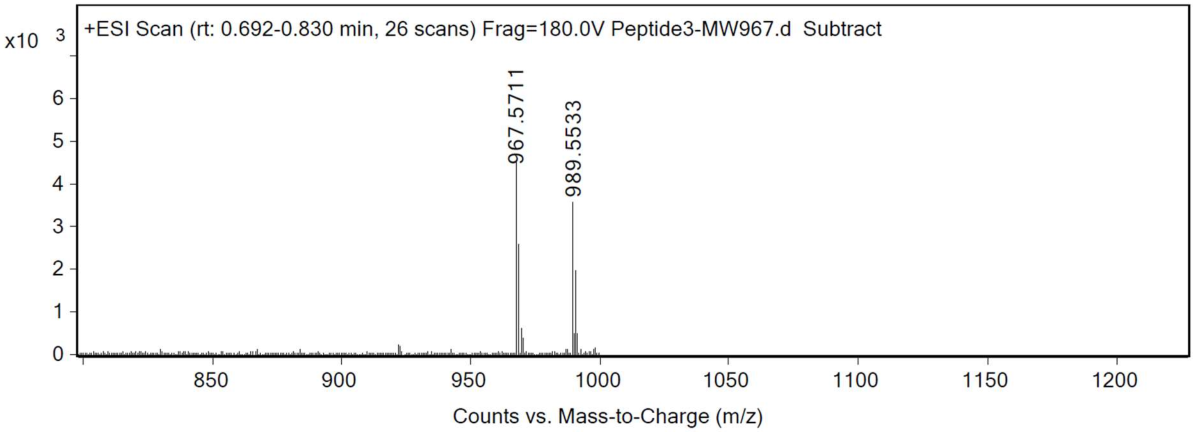

Scheme S15. Synthesis of YNV(cit)KSEM

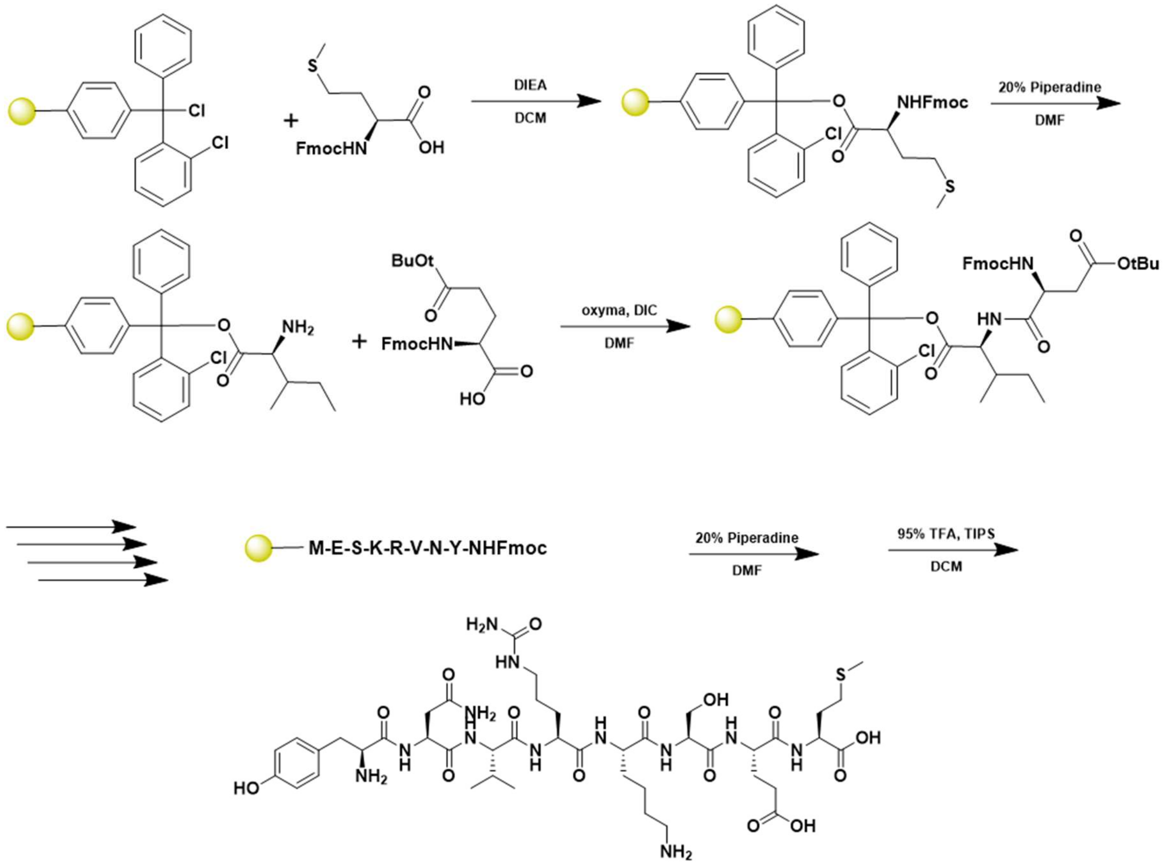

A 25 mL peptide synthesis vessel charged with 2-hlorotrityl chloride resin (0.25mmol) was added Fmoc-L-methionine-OH (1.1 eq, 0.275 mmol) and DIEA (3 eq, 0.75 mmol) in dry DCM. The resin was agitated for 1 h at ambient temperature and washed with MeOH and DCM (3 x each). The Fmoc group was removed by using a 20% piperidine solution in DMF for 30 min at ambient temperature, then washed as before. Fmoc-L-glutamate(OtBu)-OH (5 eq, 1.25 mM) along with Oxyma (5 eq, 1.25 mM) and DIC (5 eq, 1.35 mmol) in DMF was added to the reaction vessel and agitated for 2 h at ambient temperature. The Fmoc removal and coupling procedure was repeated as before using the same equivalencies for the remaining amino acids. To remove the peptide from resin, a TFA cocktail solution (95% TFA, 2.5% TIPS, and 2.5% DCM) was added to the resin and agitated for 2 hours. The resin was filtered, and the resulting solution was concentrated in vacuo. The peptide was trituated with cold diethyl ether and purified using reverse phase HPLC using H_2O/CH_3CN. The sample was analyzed for purity using a Waters 1525 Binary HPLC Pump using a Phenomenex Luna 5u C8(2) 100A (250 x 4.60 mm) column; gradient eluted with H_20/CH_3CN. Molecular weight was confirmed using high resolution electrospray ionization mass spectrometry (HRMS, ESI/MS) analyses obtained on an Agilent 6545B Q-TOF LC/MS equipped with 1260 infinity II LC system with auto sampler. The final peptide product was lyophilized and stored at -20°C until further use.

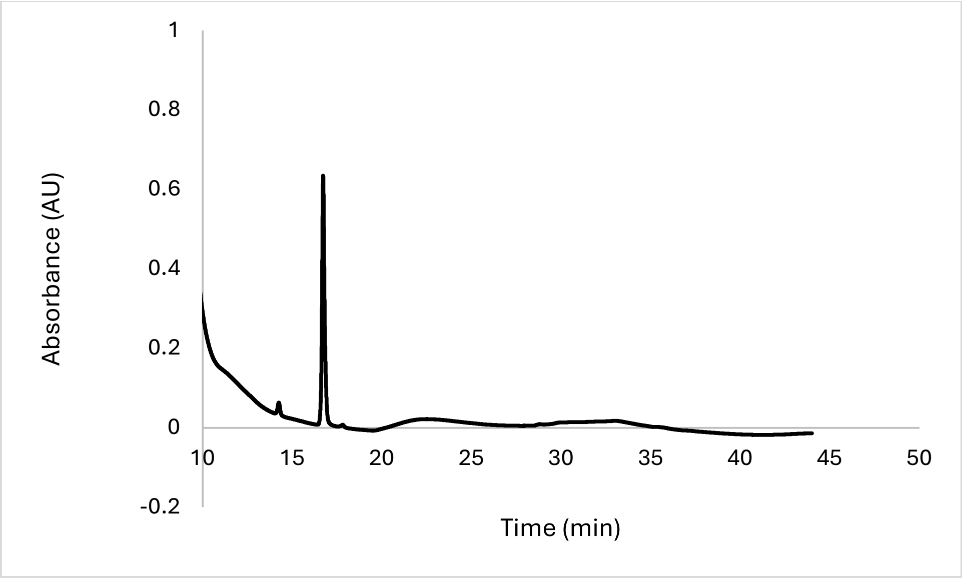

ESI-MS calculated [M]: 967.5555, found 967.5567

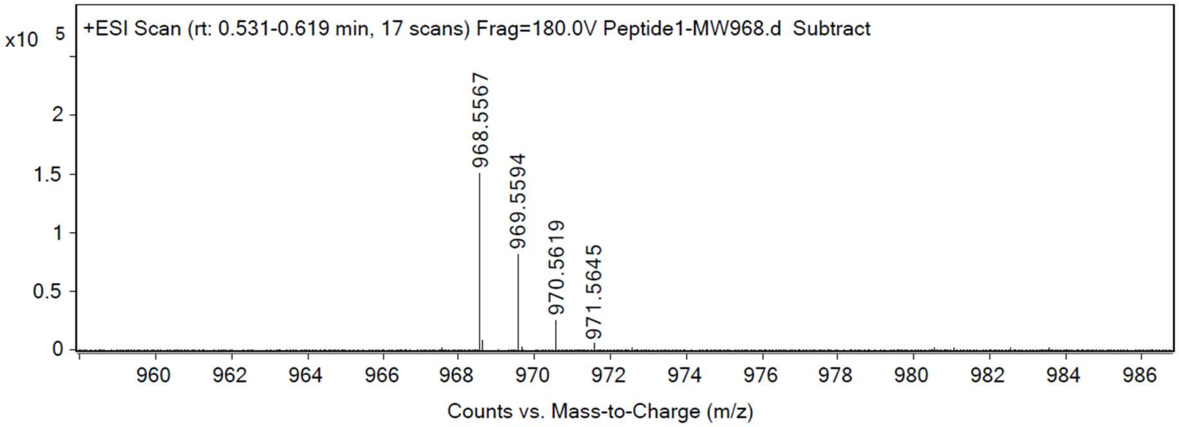

Scheme S16. Synthesis of RIYQYIQSRF

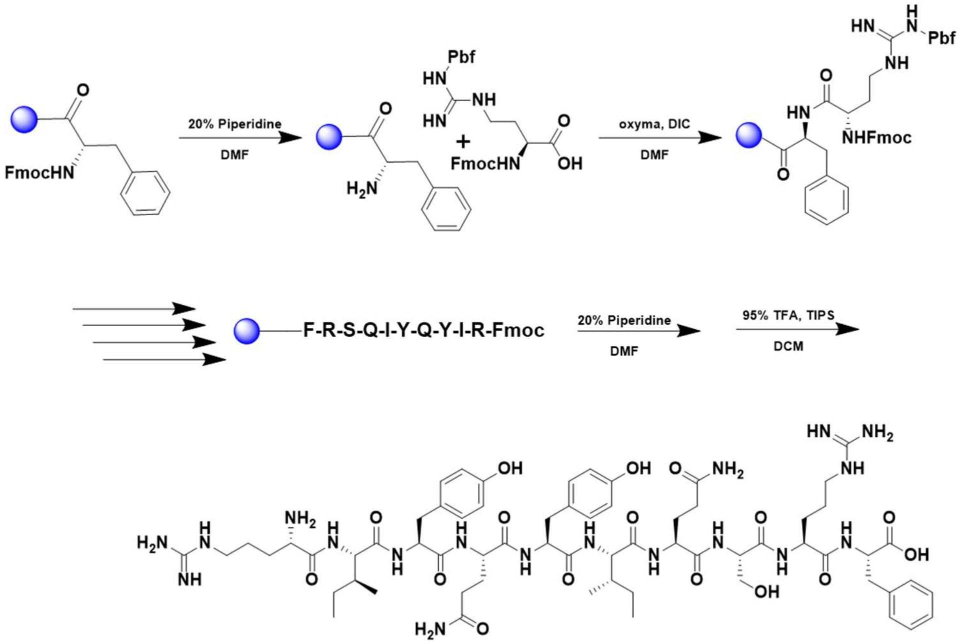

A 25 mL vessel of CEM discover bio manual peptide synthesizer was charged with 0.25 mmol of phenylalanine wang resin. The Fmoc group was removed by using a 20% piperidine solution in DMF (10 mL). Using Synergy software, the deprotection protocol was run. The piperidine solution was drained and the resin was washed with DMF (4 x 10 mL). Fmoc-L-arginine(Pbf)-OH (5 eq, 1.25 mM) along with Oxyma (5 eq, 1.25 mM) and DIC (5 eq, 1.35 mmol) in DMF was added to the reaction vessel and the coupling protocol was run. The amino acid solution was drained, and the resin was washed with DMF (2 x 10 mL). The fmoc removal and coupling procedure was repeated as before using the same equivalencies for the remaining amino acids. To remove the peptide from resin, a TFA cocktail solution (95% TFA, 2.5% TIPS, and 2.5% DCM) was added to the resin and agitated for 2 hours. The resin was filtered, and the resulting solution was concentrated in vacuo. The peptide was trituated with cold diethyl ether and purified using reverse phase HPLC using H_2O/CH_3CN. The sample was analyzed for purity using a Waters 1525 Binary HPLC Pump using a Phenomenex Luna 5u C8(2) 100A (250 x 4.60 mm) column; gradient eluted with H_20/CH_3CN. Molecular weight was confirmed using high resolution electrospray ionization mass spectrometry (HRMS, ESI/MS) analyses obtained on an Agilent 6545B Q-TOF LC/MS equipped with 1260 infinity II LC system with auto sampler. The final peptide product was lyophilized and stored at -20°C until further use.

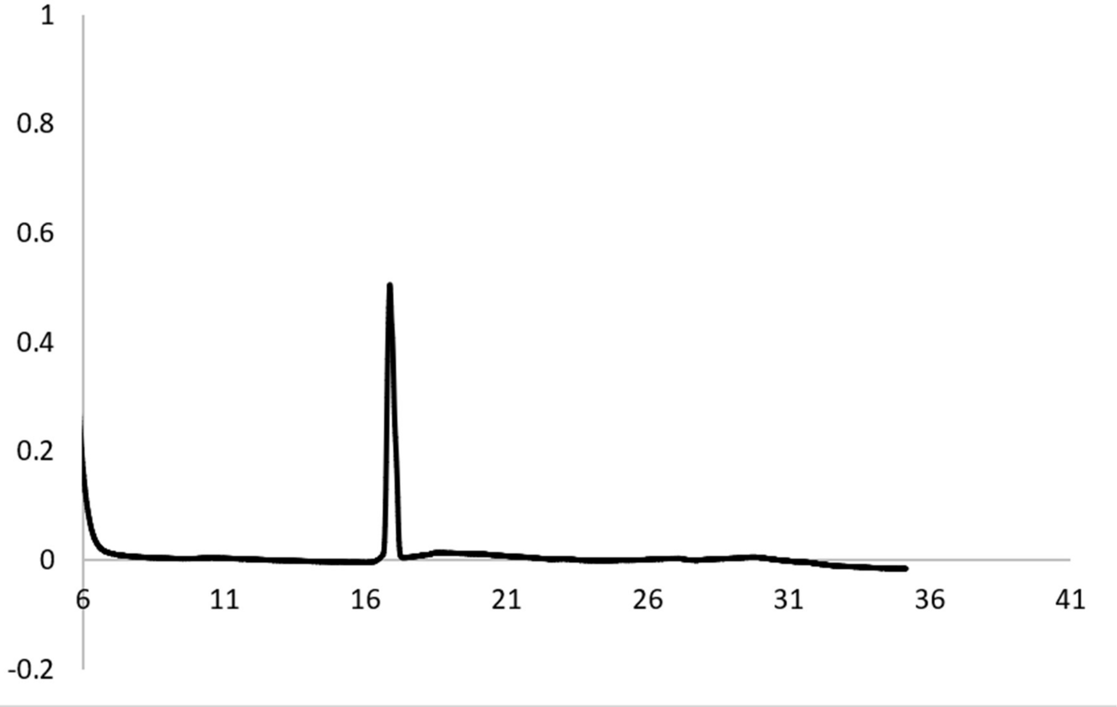

MALDI-TOF MS calculated [M]: 1373.7325, found 1373.898

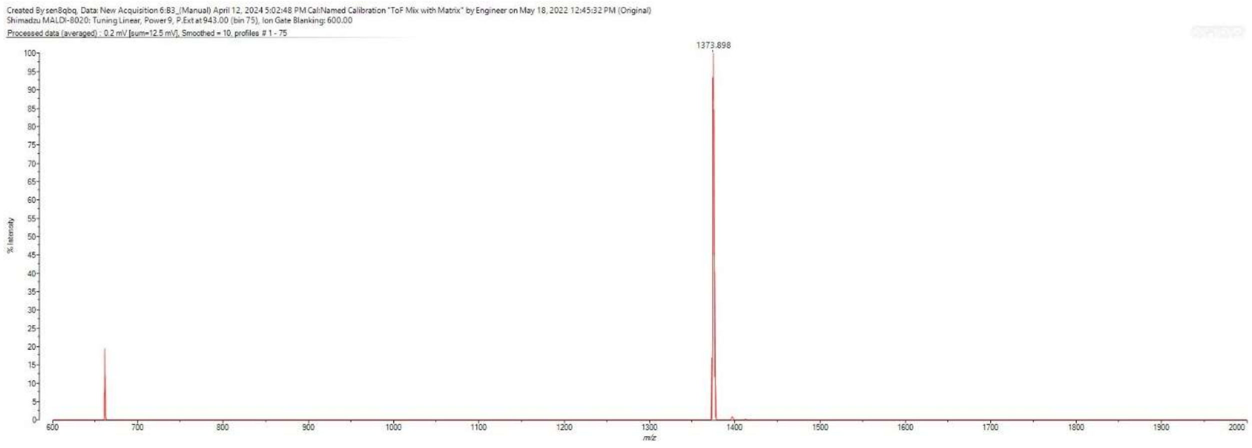

Scheme S17. Synthesis of RIYQ(YPO4)IQSRF

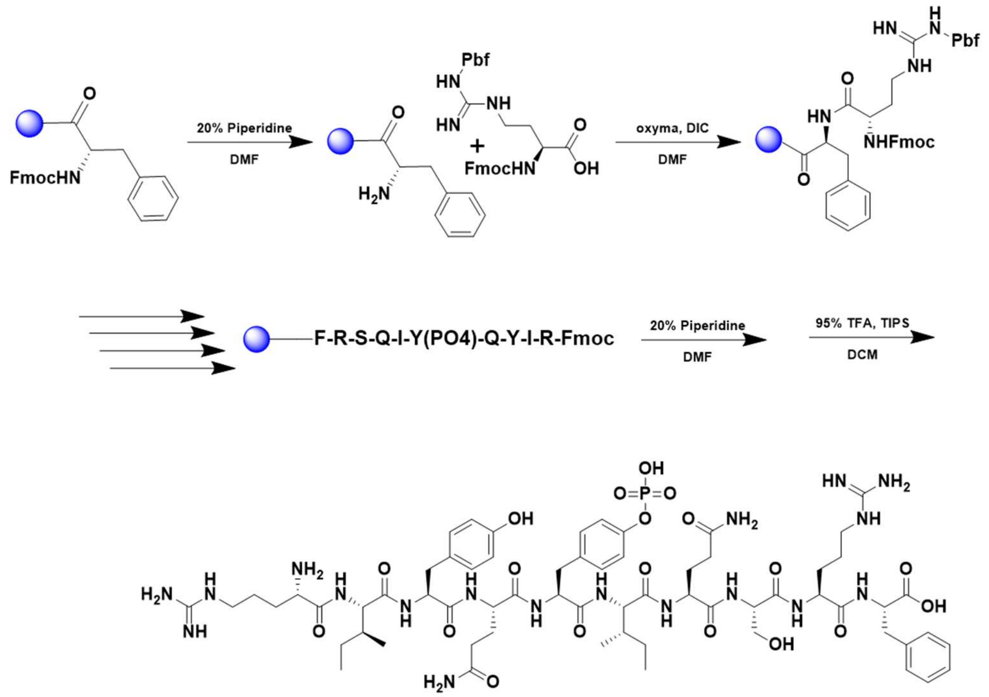

A 25 mL vessel of CEM discover bio manual peptide synthesizer was charged with 0.25 mmol of phenylalanine wang resin. The Fmoc group was removed by using a 20% piperidine solution in DMF (10 mL). Using Synergy software, the deprotection protocol was run. The piperidine solution was drained and the resin was washed with DMF (4 x 10 mL). Fmoc-L-arginine(Pbf)-OH (5 eq, 1.25 mM) along with Oxyma (5 eq, 1.25 mM) and DIC (5 eq, 1.35 mmol) in DMF was added to the reaction vessel and the coupling protocol was run. The amino acid solution was drained, and the resin was washed with DMF (2 x 10 mL). The fmoc removal and coupling procedure was repeated as before using the same equivalencies for the remaining amino acids. To remove the peptide from resin, a TFA cocktail solution (95% TFA, 2.5% TIPS, and 2.5% DCM) was added to the resin and agitated for 2 hours. The resin was filtered, and the resulting solution was concentrated in vacuo. The peptide was triturated with cold diethyl ether and purified using reverse phase HPLC using H_2O/CH_3CN. The sample was analyzed for purity using a Waters 1525 Binary HPLC Pump using a Phenomenex Luna 5u C8(2) 100A (250 x 4.60 mm) column; gradient eluted with H_20/CH_3CN. Molecular weight was confirmed using high resolution electrospray ionization mass spectrometry (HRMS, ESI/MS) analyses obtained on an Agilent 6545B Q-TOF LC/MS equipped with 1260 infinity II LC system with auto sampler. The final peptide product was lyophilized and stored at -20°C until further use.

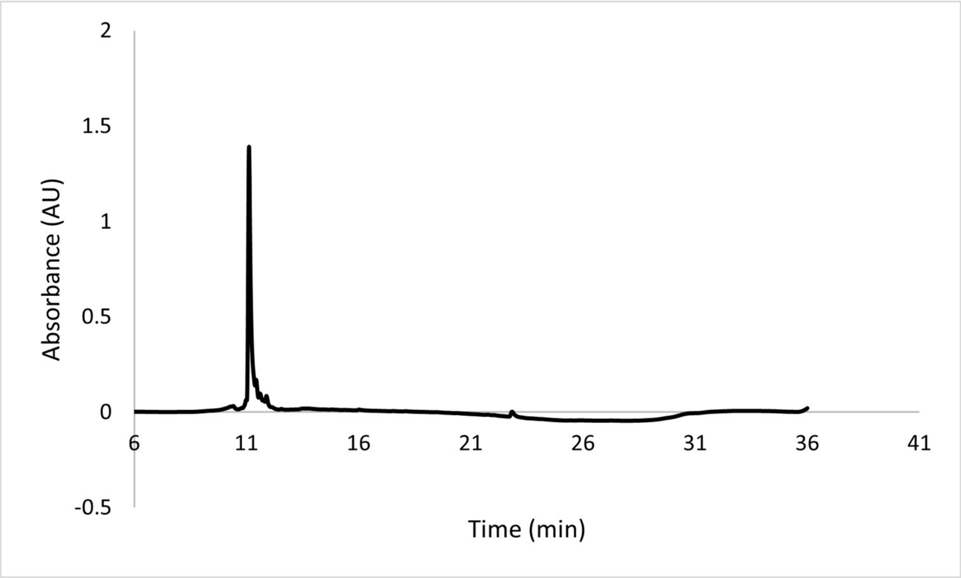

MALDI-TOF MS calculated [M]: 1452.6910, found 1455.103

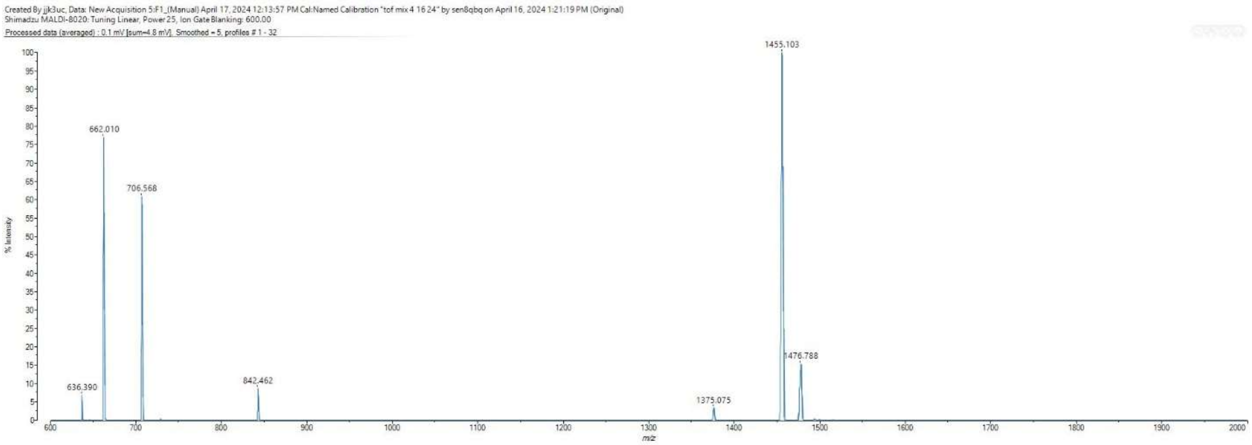

Scheme S18. Synthesis of HPDKFVGI

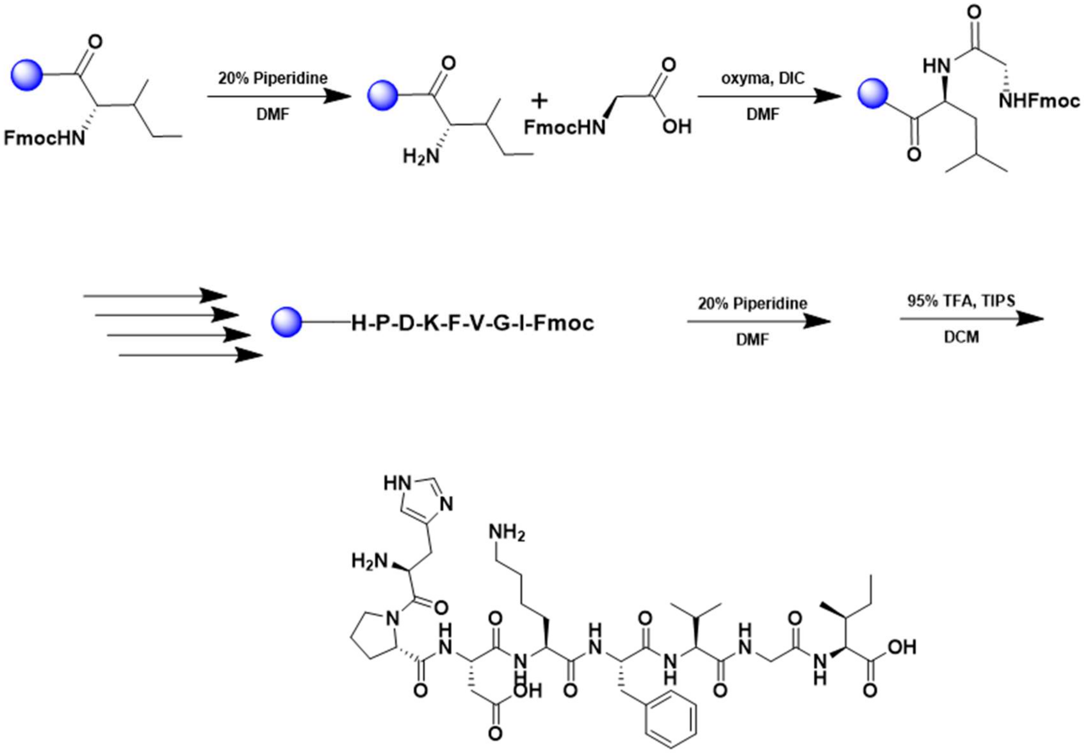

A 25 mL vessel of CEM discover bio manual peptide synthesizer was charged with 0.25 mmol of isoleucine wang resin. The Fmoc group was removed by using a 20% piperidine solution in DMF (10 mL). Using Synergy software, the deprotection protocol was run. The piperidine solution was drained and the resin was washed with DMF (4 x 10 mL). Fmoc-L-glycine (5 eq, 1.25 mM) along with Oxyma (5 eq, 1.25 mM) and DIC (5 eq, 1.35 mmol) in DMF was added to the reaction vessel and the coupling protocol was run. The amino acid solution was drained, and the resin was washed with DMF (2 x 10 mL). The fmoc removal and coupling procedure was repeated as before using the same equivalencies for the remaining amino acids. To remove the peptide from resin, a TFA cocktail solution (95% TFA, 2.5% TIPS, and 2.5% DCM) was added to the resin and agitated for 2 hours. The resin was filtered, and the resulting solution was concentrated in vacuo. The peptide was triturated with cold diethyl ether and purified using reverse phase HPLC using H_2O/CH_3CN. The sample was analyzed for purity using a Waters 1525 Binary HPLC Pump using a Phenomenex Luna 5u C8(2) 100A (250 x 4.60 mm) column; gradient eluted with H_20/CH_3CN. Molecular weight was confirmed using high resolution electrospray ionization mass spectrometry (HRMS, ESI/MS) analyses obtained on an Agilent 6545B Q-TOF LC/MS equipped with 1260 infinity II LC system with auto sampler. The final peptide product was lyophilized and stored at -20°C until further use.

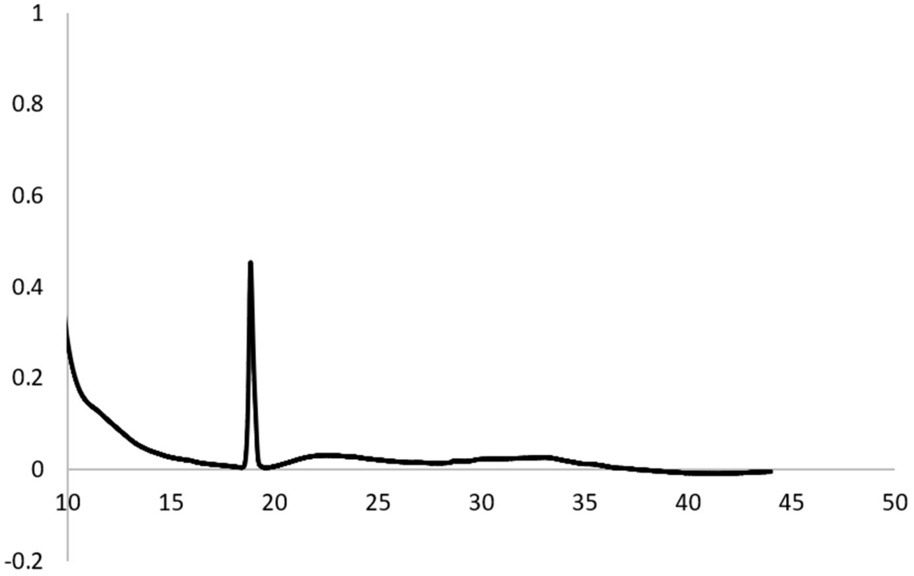

MALDI-TOF MS calculated [M]: 912.4938, found 912.758

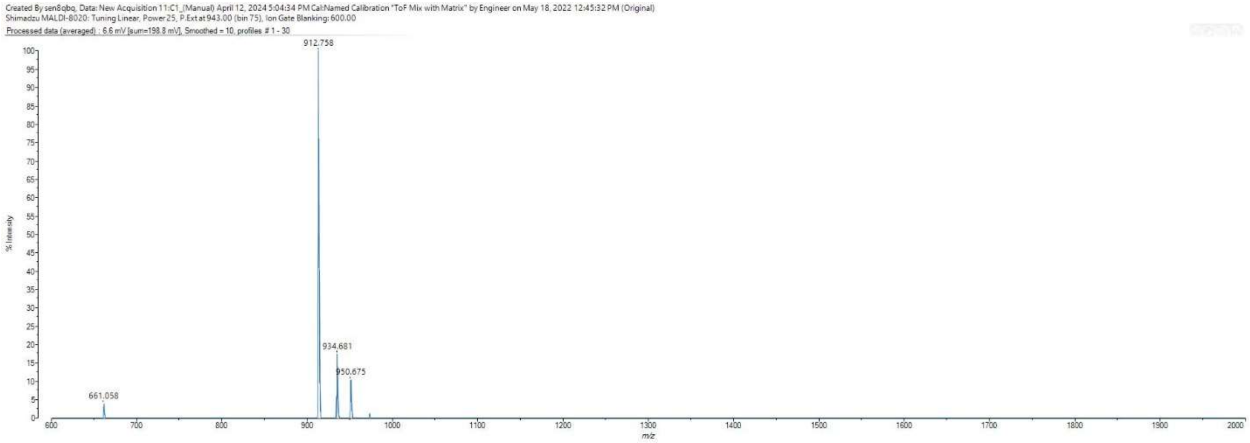

Scheme S19. Synthesis of HPD(Kac)FVGI

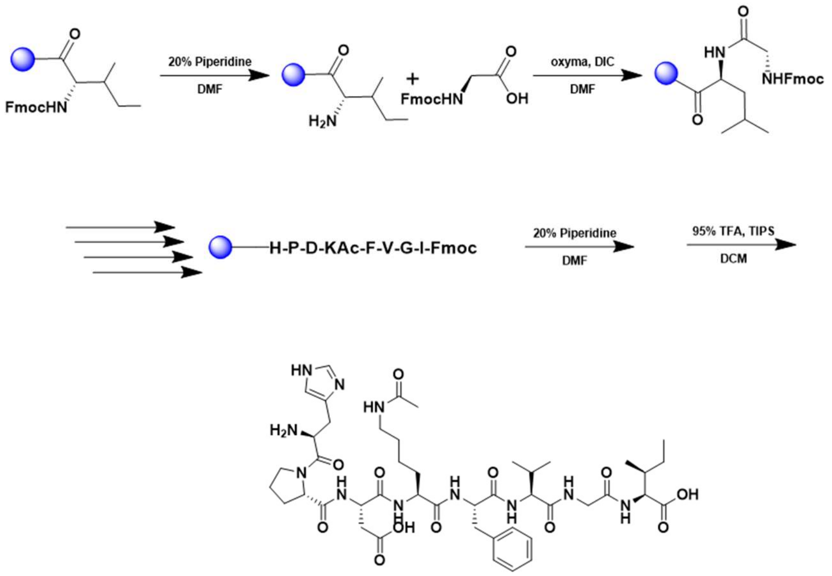

A 25 mL vessel of CEM discover bio manual peptide synthesizer was charged with 0.25 mmol of isoleucine wang resin. The Fmoc group was removed by using a 20% piperidine solution in DMF (10 mL). Using Synergy software, the deprotection protocol was run. The piperidine solution was drained and the resin was washed with DMF (4 x 10 mL). Fmoc-L-glycine (5 eq, 1.25 mM) along with Oxyma (5 eq, 1.25 mM) and DIC (5 eq, 1.35 mmol) in DMF was added to the reaction vessel and the coupling protocol was run. The amino acid solution was drained, and the resin was washed with DMF (2 x 10 mL). The fmoc removal and coupling procedure was repeated as before using the same equivalencies for the remaining amino acids. To remove the peptide from resin, a TFA cocktail solution (95% TFA, 2.5% TIPS, and 2.5% DCM) was added to the resin and agitated for 2 hours. The resin was filtered, and the resulting solution was concentrated in vacuo. The peptide was trituated with cold diethyl ether and purified using reverse phase HPLC using H_2O/CH_3CN. The sample was analyzed for purity using a Waters 1525 Binary HPLC Pump using a Phenomenex Luna 5u C8(2) 100A (250 x 4.60 mm) column; gradient eluted with H_20/CH_3CN. Molecular weight was confirmed using high resolution electrospray ionization mass spectrometry (HRMS, ESI/MS) analyses obtained on an Agilent 6545B Q-TOF LC/MS equipped with 1260 infinity II LC system with auto sampler. The final peptide product was lyophilized and stored at -20°C until further use.

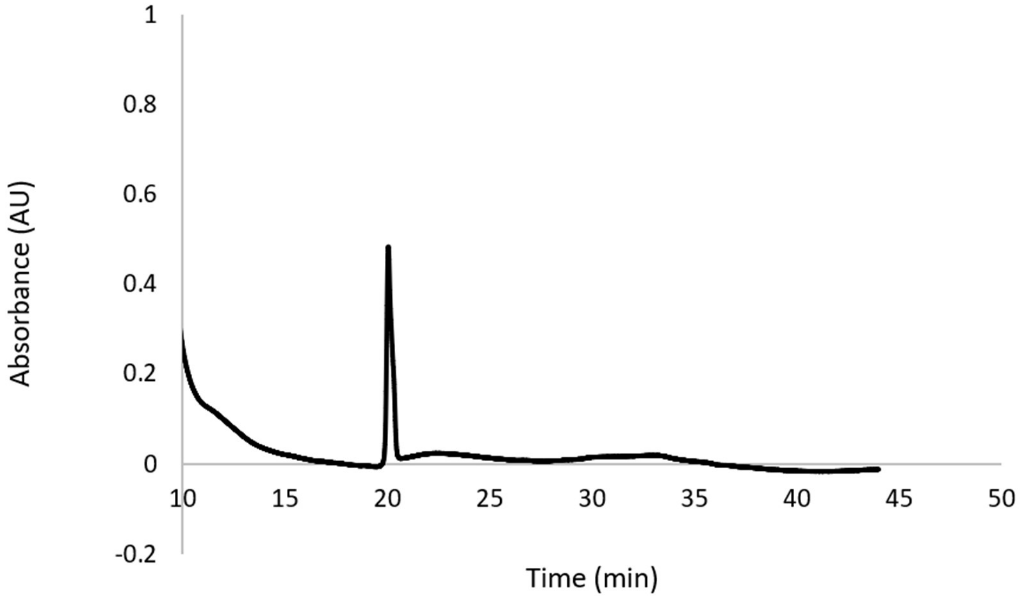

MALDI-TOF MS calculated [M]: 954.5044, found 954.917

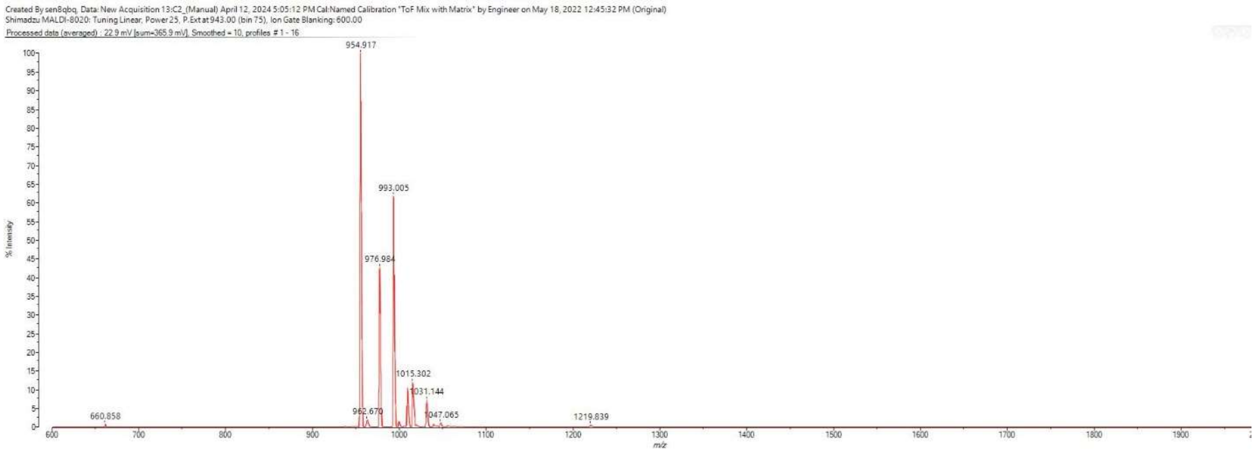

Scheme S20. Synthesis of ANLERTF

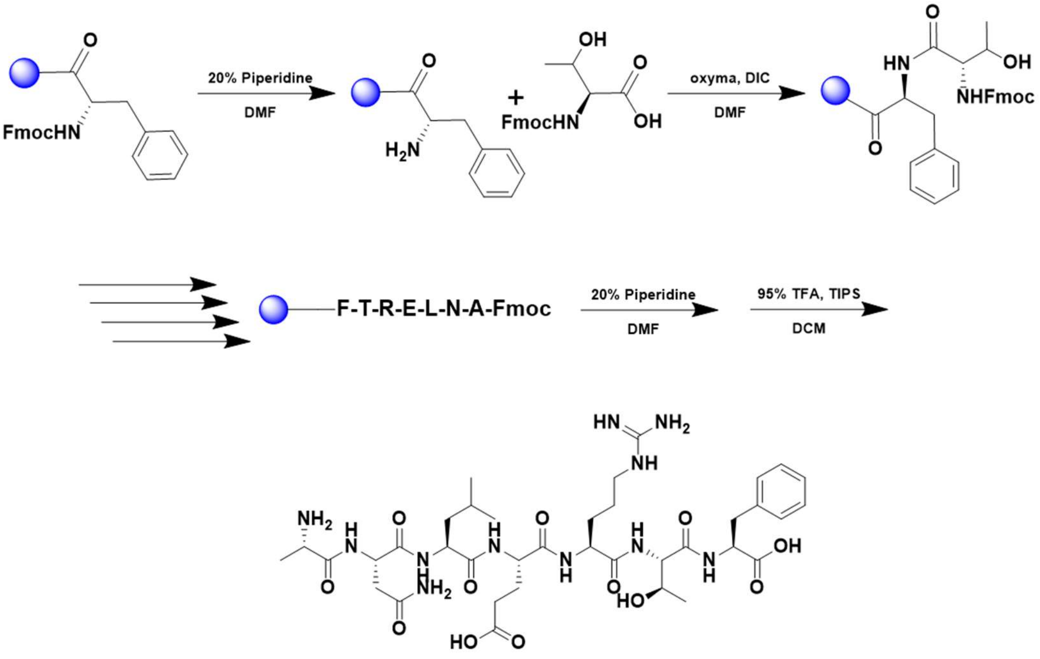

A 25 mL vessel of CEM discover bio manual peptide synthesizer was charged with 0.25 mmol of phenylalanine wang resin. The Fmoc group was removed by using a 20% piperidine solution in DMF (10 mL). Using Synergy software, the deprotection protocol was run. The piperidine solution was drained and the resin was washed with DMF (4 x 10 mL). Fmoc-L-threonine(tBu)-OH (5 eq, 1.25 mM) along with Oxyma (5 eq, 1.25 mM) and DIC (5 eq, 1.35 mmol) in DMF was added to the reaction vessel and the coupling protocol was run. The amino acid solution was drained, and the resin was washed with DMF (2 x 10 mL). The fmoc removal and coupling procedure was repeated as before using the same equivalencies for the remaining amino acids. To remove the peptide from resin, a TFA cocktail solution (95% TFA, 2.5% TIPS, and 2.5% DCM) was added to the resin and agitated for 2 hours. The resin was filtered, and the resulting solution was concentrated in vacuo. The peptide was trituated with cold diethyl ether and purified using reverse phase HPLC using H_2O/CH_3CN. The sample was analyzed for purity using a Waters 1525 Binary HPLC Pump using a Phenomenex Luna 5u C8(2) 100A (250 x 4.60 mm) column; gradient eluted with H_20/CH_3CN. Molecular weight was confirmed using high resolution electrospray ionization mass spectrometry (HRMS, ESI/MS) analyses obtained on an Agilent 6545B Q-TOF LC/MS equipped with 1260 infinity II LC system with auto sampler. The final peptide product was lyophilized and stored at -20°C until further use.

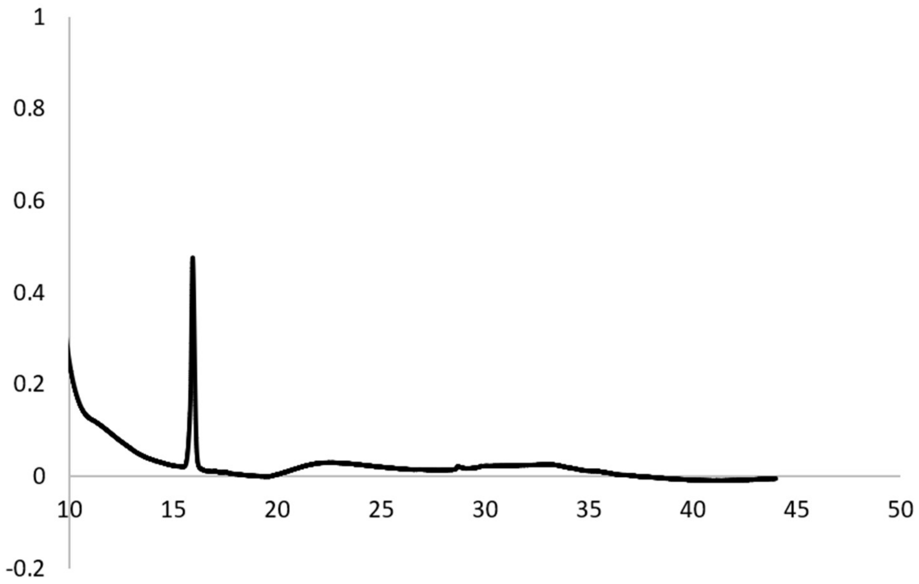

MALDI-TOF MS calculated [M]: 737.4345, found 737.364

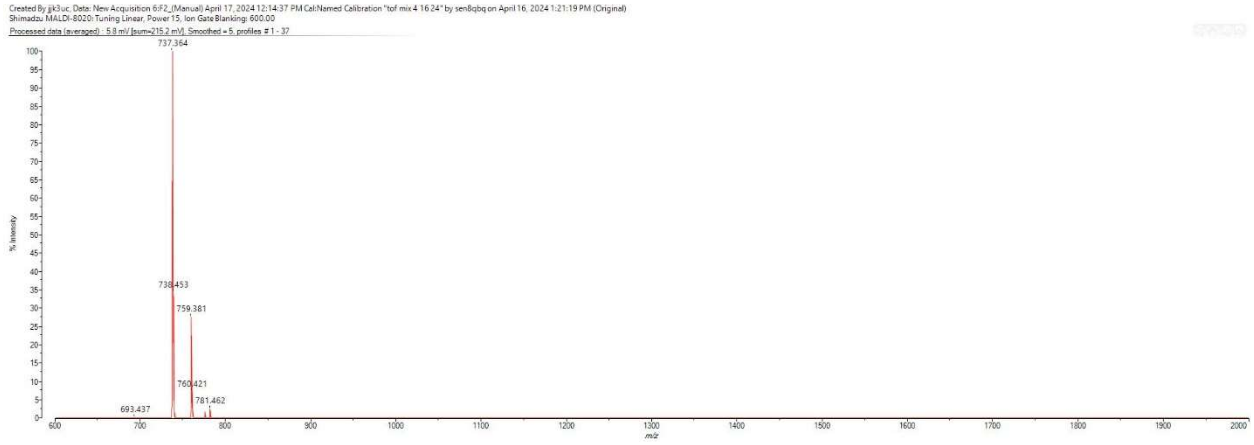

Scheme S21. Synthesis of NAc-ANLERTF

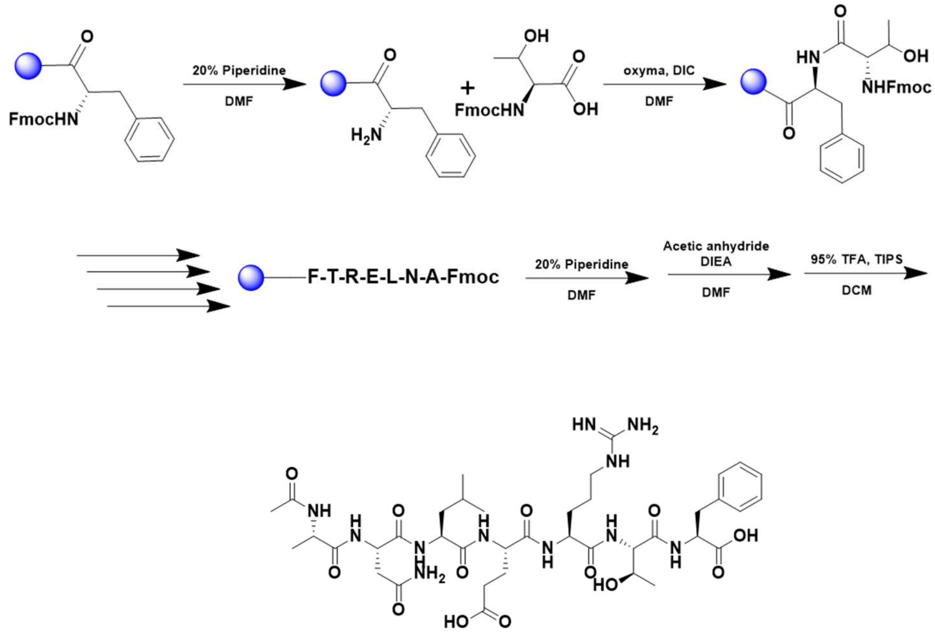

A 25 mL vessel of CEM discover bio manual peptide synthesizer was charged with 0.25 mmol of phenylalanine wang resin. The Fmoc group was removed by using a 20% piperidine solution in DMF (10 mL). Using Synergy software, the deprotection protocol was run. The piperidine solution was drained and the resin was washed with DMF (4 x 10 mL). Fmoc-L-threonine (5 eq, 1.25 mM) along with Oxyma (5 eq, 1.25 mM) and DIC (5 eq, 1.35 mmol) in DMF was added to the reaction vessel and the coupling protocol was run. The amino acid solution was drained, and the resin was washed with DMF (2 x 10 mL). The final amino acid was Fmoc deprotected as described before and was acetylated by agitating the resin for 1 hour in a solution of 5% acetic anhydride (0.5 mL), 8.5% DIEA (0.85 mL), and 86.5% DMF (8.65 mL). To remove the peptide from resin, a TFA cocktail solution (95% TFA, 2.5% TIPS, and 2.5% DCM) was added to the resin and agitated for 2 hours. The resin was filtered, and the resulting solution was concentrated in vacuo. The peptide was triturated with cold diethyl ether and purified using reverse phase HPLC using H_2O/CH_3CN. The sample was analyzed for purity using a Waters 1525 Binary HPLC Pump using a Phenomenex Luna 5u C8(2) 100A (250 x 4.60 mm) column; gradient eluted with H_20/CH_3CN. Molecular weight was confirmed using high resolution electrospray ionization mass spectrometry (HRMS, ESI/MS) analyses obtained on an Agilent 6545B Q-TOF LC/MS equipped with 1260 infinity II LC system with auto sampler. The final peptide product was lyophilized and stored at -20°C until further use.

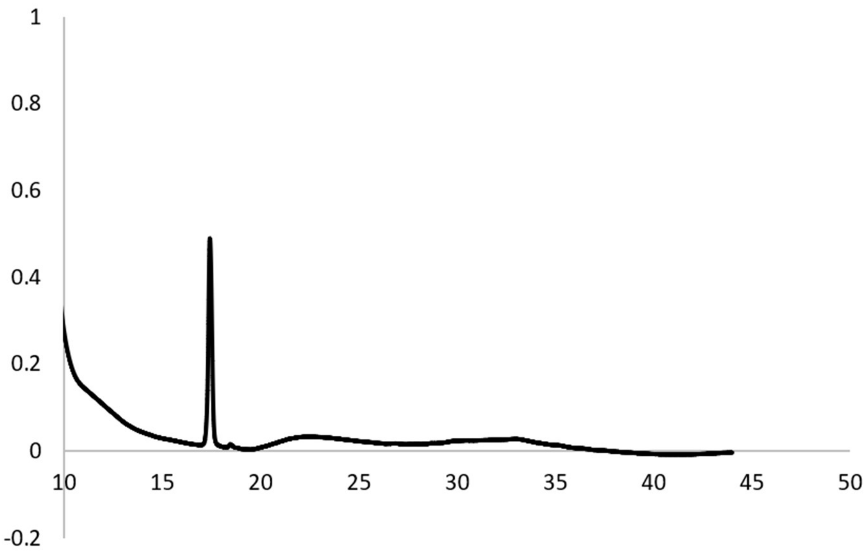

MALDI-TOF MS calculated [M]: 780.4450, found 780.387

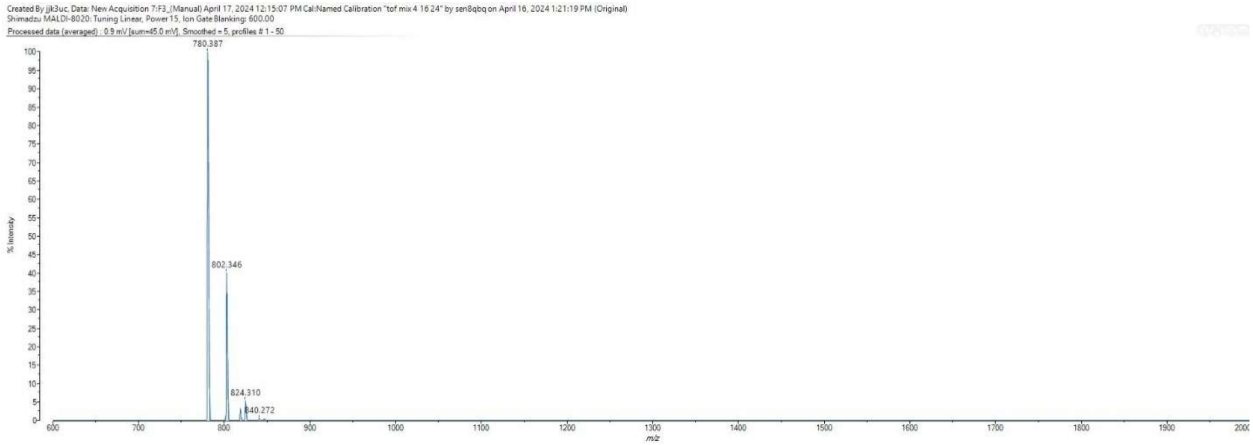

Scheme S22. Synthesis of SAIQNHSF

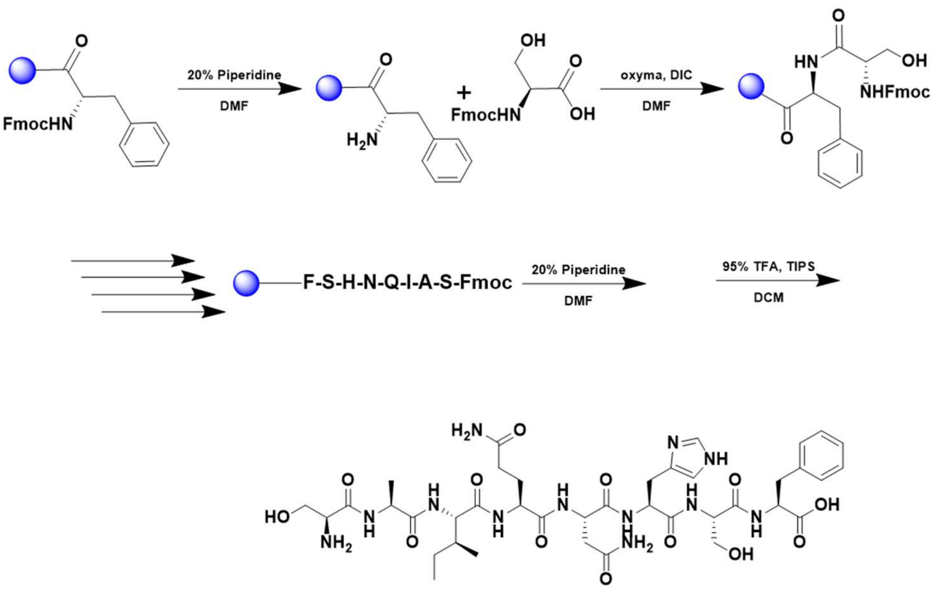

A 25 mL vessel of CEM discover bio manual peptide synthesizer was charged with 0.25 mmol of phenylalanine wang resin. The Fmoc group was removed by using a 20% piperidine solution in DMF (10 mL). Using Synergy software, the deprotection protocol was run. The piperidine solution was drained and the resin was washed with DMF (4 x 10 mL). Fmoc-L-serine(tBu)-OH (5 eq, 1.25 mM) along with Oxyma (5 eq, 1.25 mM) and DIC (5 eq, 1.35 mmol) in DMF was added to the reaction vessel and the coupling protocol was run. The amino acid solution was drained, and the resin was washed with DMF (2 x 10 mL). The fmoc removal and coupling procedure was repeated as before using the same equivalencies for the remaining amino acids. To remove the peptide from resin, a TFA cocktail solution (95% TFA, 2.5% TIPS, and 2.5% DCM) was added to the resin and agitated for 2 hours. The resin was filtered, and the resulting solution was concentrated in vacuo. The peptide was trituated with cold diethyl ether and purified using reverse phase HPLC using H_2O/CH_3CN. The sample was analyzed for purity using a Waters 1525 Binary HPLC Pump using a Phenomenex Luna 5u C8(2) 100A (250 x 4.60 mm) column; gradient eluted with H_20/CH_3CN. Molecular weight was confirmed using high resolution electrospray ionization mass spectrometry (HRMS, ESI/MS) analyses obtained on an Agilent 6545B Q-TOF LC/MS equipped with 1260 infinity II LC system with auto sampler. The final peptide product was lyophilized and stored at -20°C until further use.

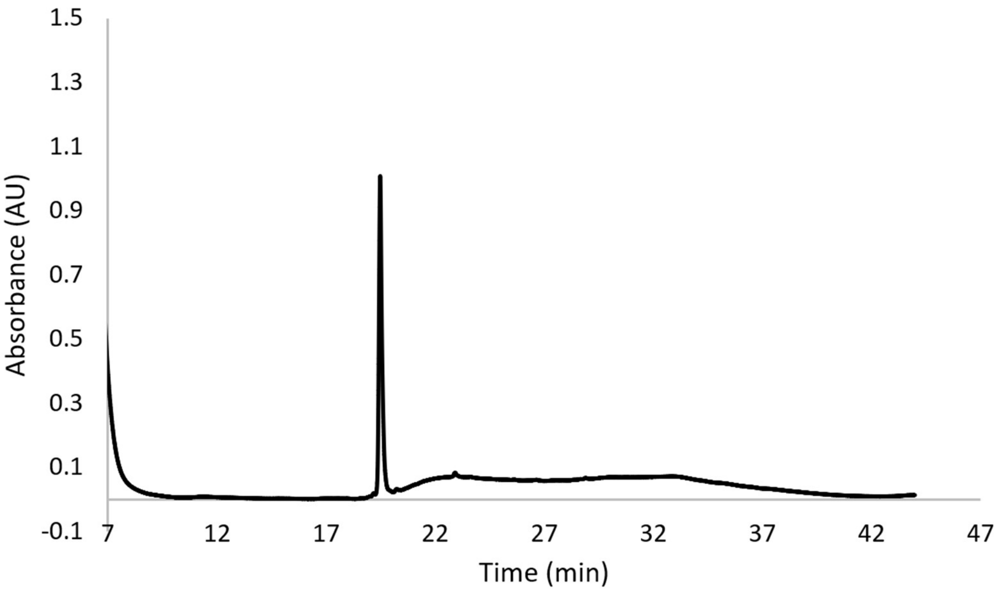

MALDI-TOF MS calculated [M]: 1016.4246, found 1016.687

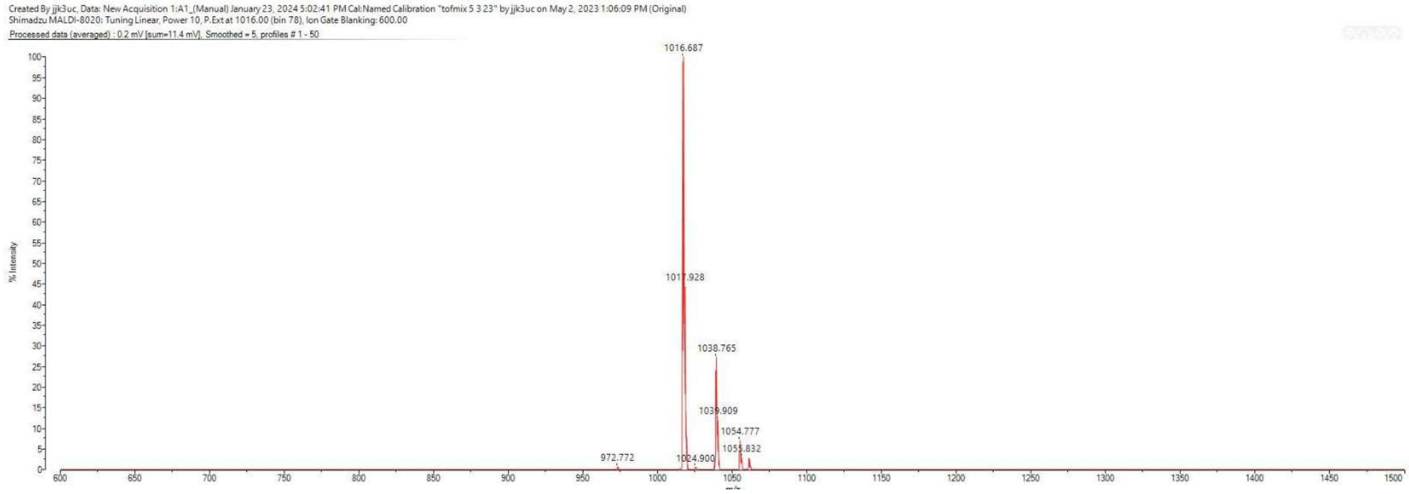

Scheme S23. Synthesis of NAc-SAIQNHSF

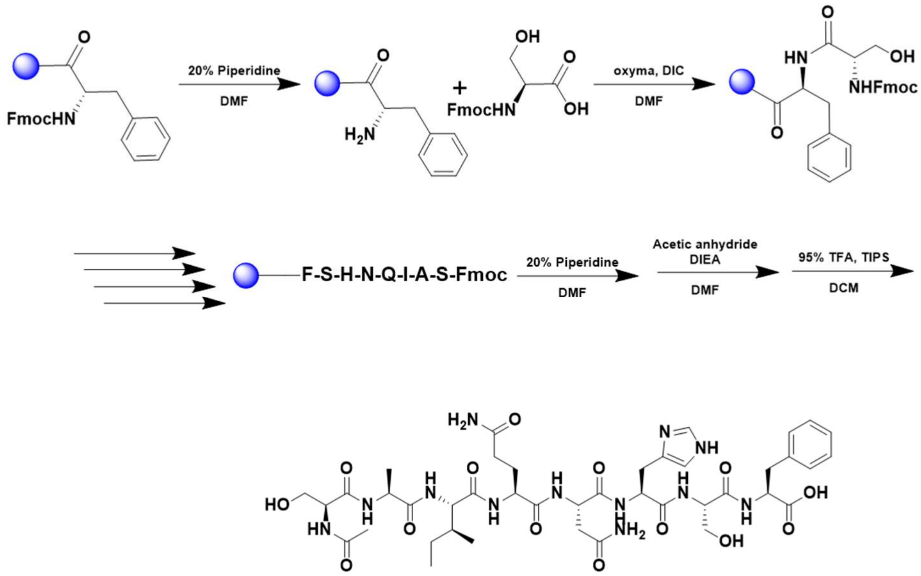

A 25 mL vessel of CEM discover bio manual peptide synthesizer was charged with 0.25 mmol of phenylalanine wang resin. The Fmoc group was removed by using a 20% piperidine solution in DMF (10 mL). Using Synergy software, the deprotection protocol was run. The piperidine solution was drained and the resin was washed with DMF (4 x 10 mL). Fmoc-L-serine-OH (5 eq, 1.25 mM) along with Oxyma (5 eq, 1.25 mM) and DIC (5 eq, 1.35 mmol) in DMF was added to the reaction vessel and the coupling protocol was run. The amino acid solution was drained, and the resin was washed with DMF (2 x 10 mL). The final amino acid was Fmoc deprotected as described before and was acetylated by agitating the resin for 1 hour in a solution of 5% acetic anhydride (0.5 mL), 8.5% DIEA (0.85 mL), and 86.5% DMF (8.65 mL). To remove the peptide from resin, a TFA cocktail solution (95% TFA, 2.5% TIPS, and 2.5% DCM) was added to the resin and agitated for 2 hours. The resin was filtered, and the resulting solution was concentrated in vacuo. The peptide was trituated with cold diethyl ether and purified using reverse phase HPLC using H_2O/CH_3CN. The sample was analyzed for purity using a Waters 1525 Binary HPLC Pump using a Phenomenex Luna 5u C8(2) 100A (250 x 4.60 mm) column; gradient eluted with H_20/CH_3CN. Molecular weight was confirmed using high resolution electrospray ionization mass spectrometry (HRMS, ESI/MS) analyses obtained on an Agilent 6545B Q-TOF LC/MS equipped with 1260 infinity II LC system with auto sampler. The final peptide product was lyophilized and stored at -20°C until further use.

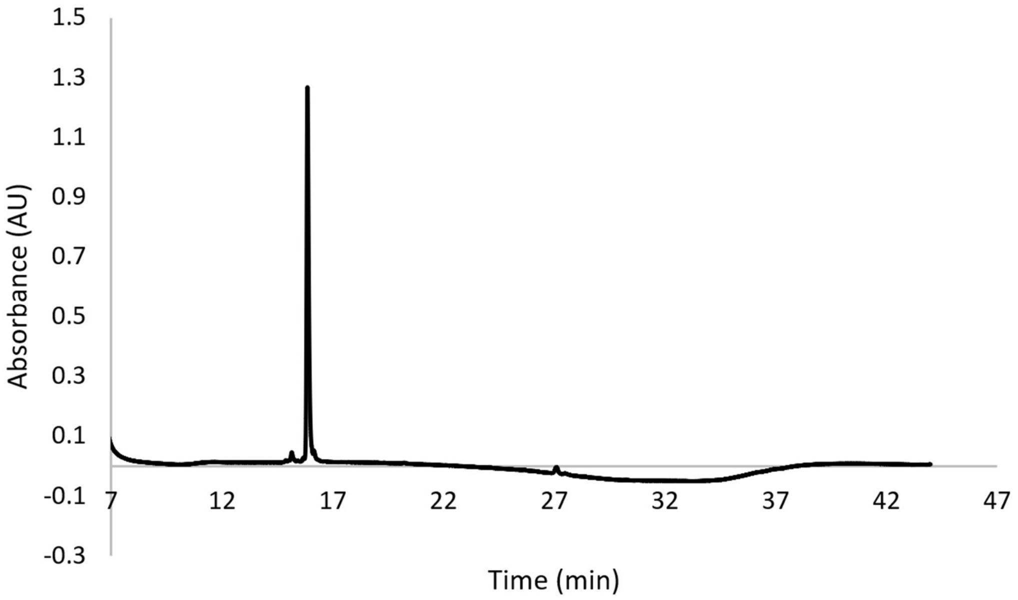

ESI-MS calculated [M]: 1059.4351, found 1059.

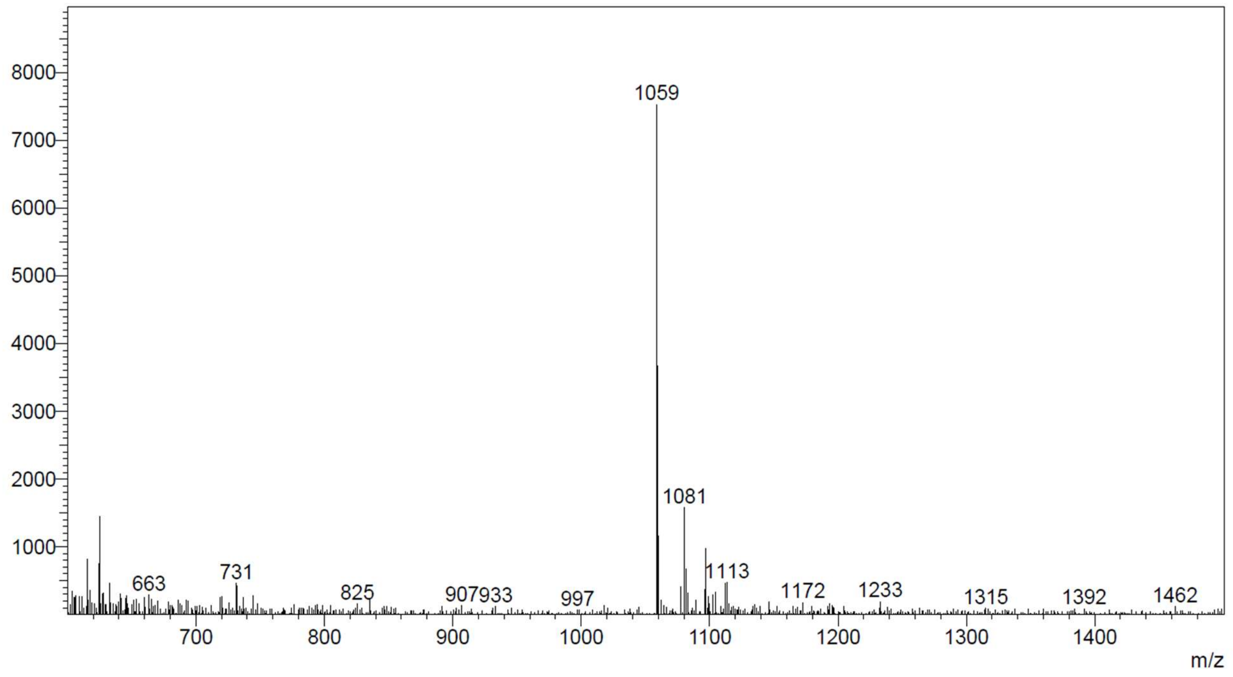

Scheme S24. Synthesis of TVFVFKRA

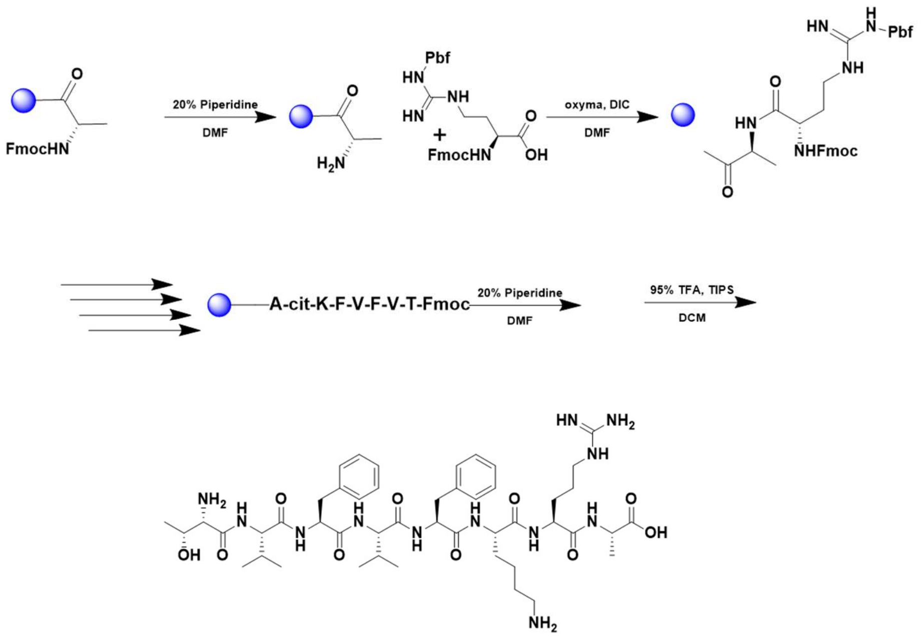

A 25 mL vessel of CEM discover bio manual peptide synthesizer was charged with 0.25 mmol of alanine wang resin. The Fmoc group was removed by using a 20% piperidine solution in DMF (10 mL). Using Synergy software, the deprotection protocol was run. The piperidine solution was drained and the resin was washed with DMF (4 x 10 mL). Fmoc-L-arginine(Pbf)-OH (5 eq, 1.25 mM) along with Oxyma (5 eq, 1.25 mM) and DIC (5 eq, 1.35 mmol) in DMF was added to the reaction vessel and the coupling protocol was run. The amino acid solution was drained, and the resin was washed with DMF (2 x 10 mL). The fmoc removal and coupling procedure was repeated as before using the same equivalencies for the remaining amino acids. To remove the peptide from resin, a TFA cocktail solution (95% TFA, 2.5% TIPS, and 2.5% DCM) was added to the resin and agitated for 2 hours. The resin was filtered, and the resulting solution was concentrated in vacuo. The peptide was trituated with cold diethyl ether and purified using reverse phase HPLC using H_2O/CH_3CN. The sample was analyzed for purity using a Waters 1525 Binary HPLC Pump using a Phenomenex Luna 5u C8(2) 100A (250 x 4.60 mm) column; gradient eluted with H_20/CH_3CN. Molecular weight was confirmed using high resolution electrospray ionization mass spectrometry (HRMS, ESI/MS) analyses obtained on an Agilent 6545B Q-TOF LC/MS equipped with 1260 infinity II LC system with auto sampler. The final peptide product was lyophilized and stored at -20°C until further use.

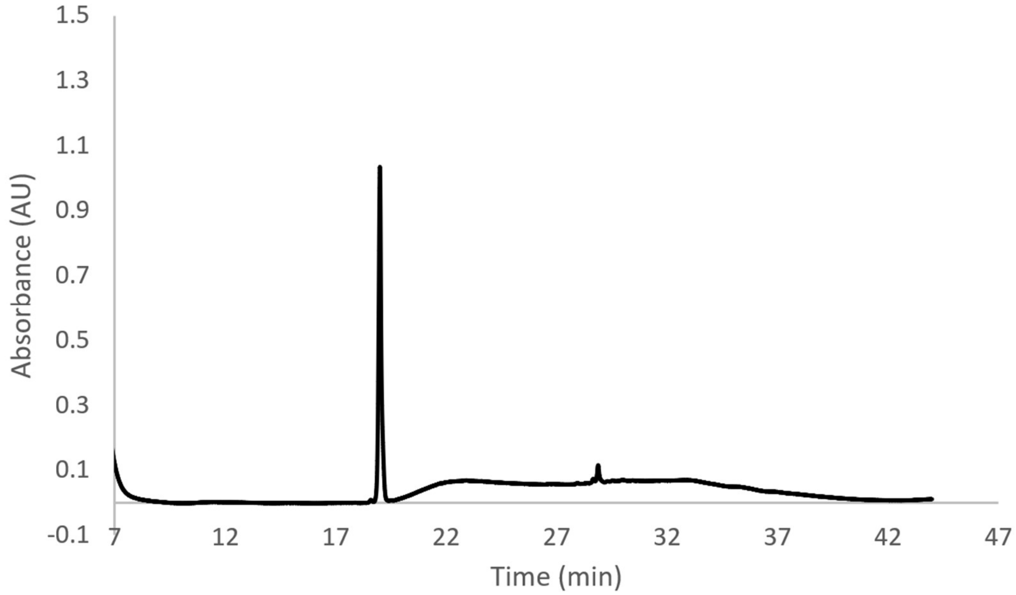

MALDI-TOF MS calculated [M]: 967.5651, found 967.864

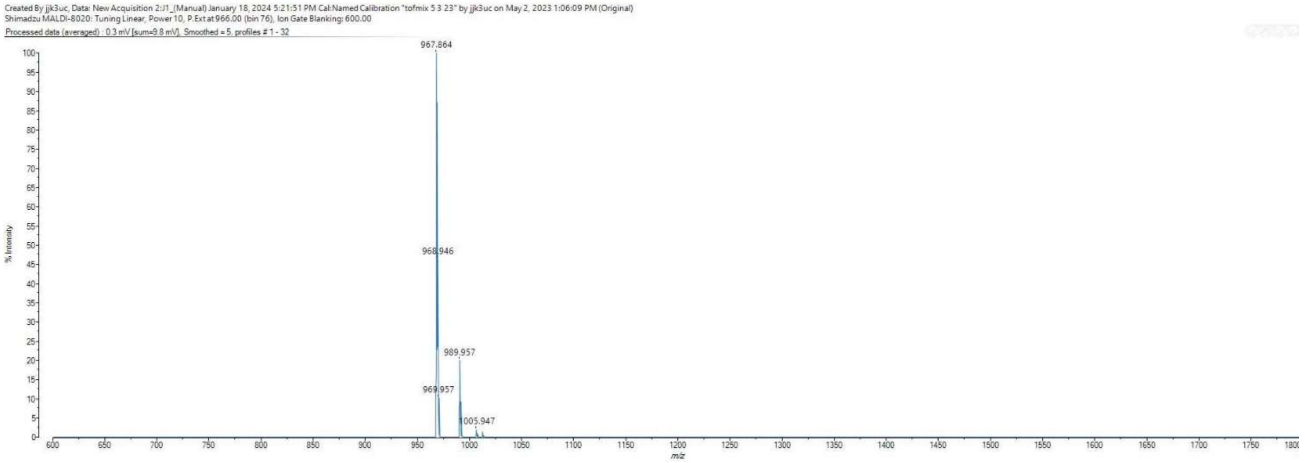

Scheme S25. Synthesis of TVFVFK(cit)A

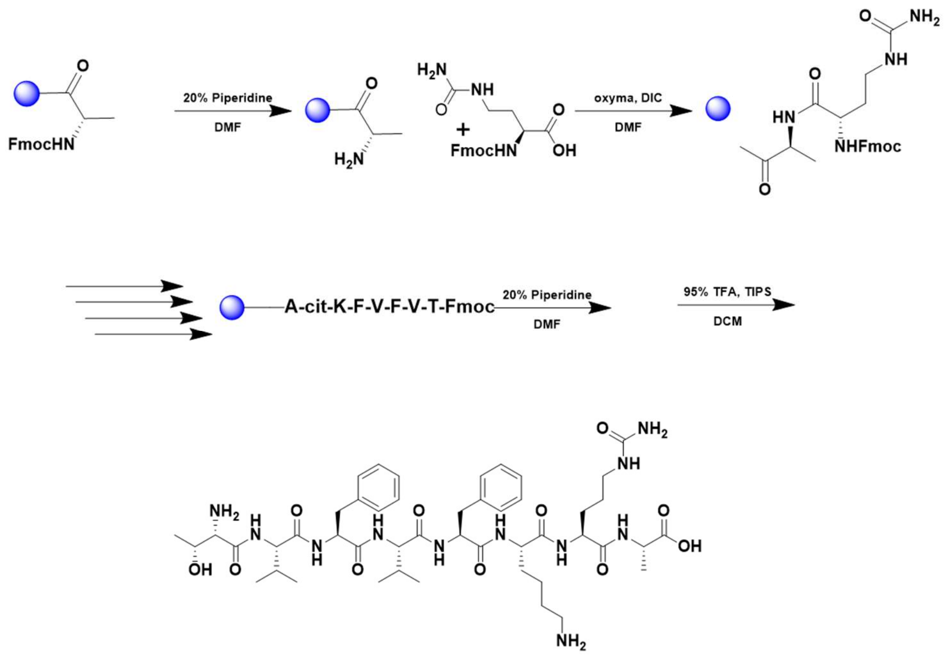

A 25 mL vessel of CEM discover bio manual peptide synthesizer was charged with 0.25 mmol of alanine wang resin. The Fmoc group was removed by using a 20% piperidine solution in DMF (10 mL). Using Synergy software, the deprotection protocol was run. The piperidine solution was drained and the resin was washed with DMF (4 x 10 mL). Fmoc-L-citruline (5 eq, 1.25 mM) along with Oxyma (5 eq, 1.25 mM) and DIC (5 eq, 1.35 mmol) in DMF was added to the reaction vessel and the coupling protocol was run. The amino acid solution was drained, and the resin was washed with DMF (2 x 10 mL). The fmoc removal and coupling procedure was repeated as before using the same equivalencies for the remaining amino acids. To remove the peptide from resin, a TFA cocktail solution (95% TFA, 2.5% TIPS, and 2.5% DCM) was added to the resin and agitated for 2 hours. The resin was filtered, and the resulting solution was concentrated in vacuo. The peptide was trituated with cold diethyl ether and purified using reverse phase HPLC using H_2O/CH_3CN. The sample was analyzed for purity using a Waters 1525 Binary HPLC Pump using a Phenomenex Luna 5u C8(2) 100A (250 x 4.60 mm) column; gradient eluted with H_20/CH_3CN. Molecular weight was confirmed using high resolution electrospray ionization mass spectrometry (HRMS, ESI/MS) analyses obtained on an Agilent 6545B Q-TOF LC/MS equipped with 1260 infinity II LC system with auto sampler. The final peptide product was lyophilized and stored at -20°C until further use.

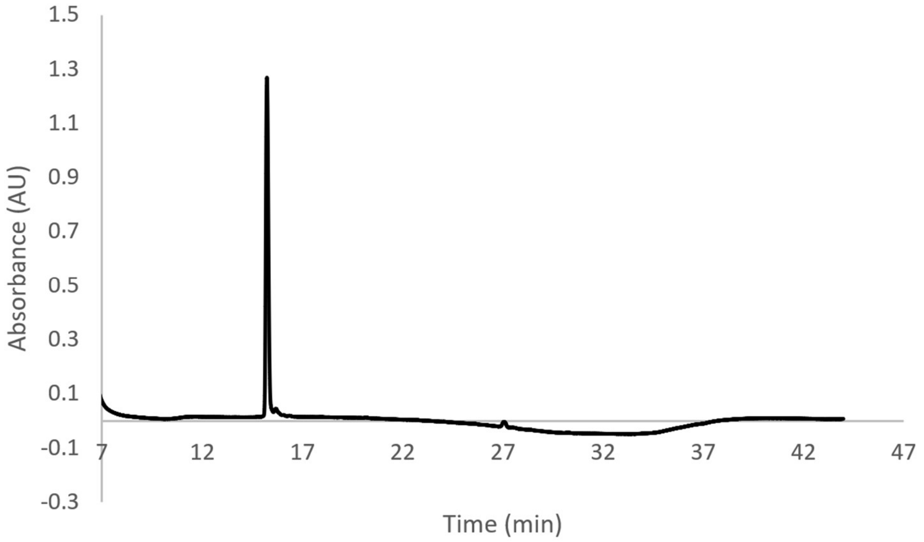

MALDI-TOF MS calculated [M]: 968.5525, found 968.375

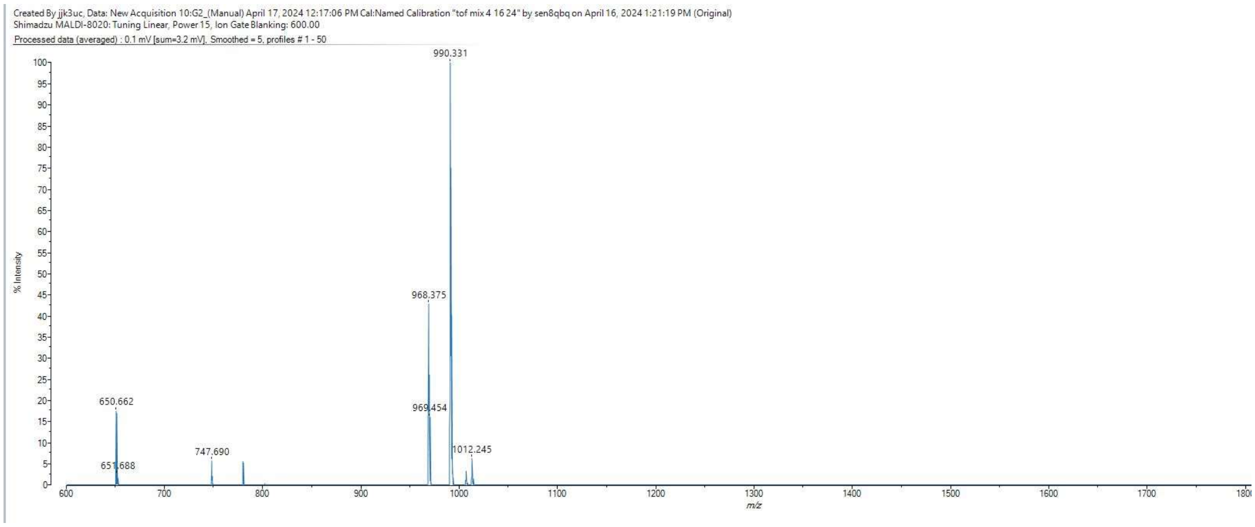

Scheme S26. Synthesis of SGIAKTAL

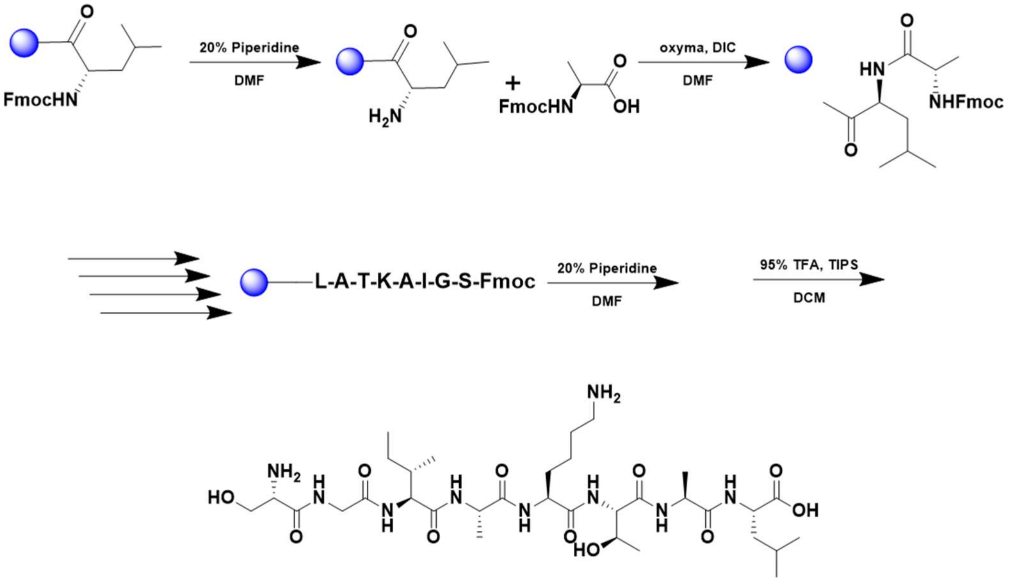

A 25 mL vessel of CEM discover bio manual peptide synthesizer was charged with 0.25 mmol of isoleucine wang resin. The Fmoc group was removed by using a 20% piperidine solution in DMF (10 mL). Using Synergy software, the deprotection protocol was run. The piperidine solution was drained and the resin was washed with DMF (4 x 10 mL). Fmoc-L-alanine (5 eq, 1.25 mM) along with Oxyma (5 eq, 1.25 mM) and DIC (5 eq, 1.35 mmol) in DMF was added to the reaction vessel and the coupling protocol was run. The amino acid solution was drained, and the resin was washed with DMF (2 x 10 mL). The fmoc removal and coupling procedure was repeated as before using the same equivalencies for the remaining amino acids. To remove the peptide from resin, a TFA cocktail solution (95% TFA, 2.5% TIPS, and 2.5% DCM) was added to the resin and agitated for 2 hours. The resin was filtered, and the resulting solution was concentrated in vacuo. The peptide was trituated with cold diethyl ether and purified using reverse phase HPLC using H_2O/CH_3CN. The sample was analyzed for purity using a Waters 1525 Binary HPLC Pump using a Phenomenex Luna 5u C8(2) 100A (250 x 4.60 mm) column; gradient eluted with H_20/CH_3CN. Molecular weight was confirmed using high resolution electrospray ionization mass spectrometry (HRMS, ESI/MS) analyses obtained on an Agilent 6545B Q-TOF LC/MS equipped with 1260 infinity II LC system with auto sampler. The final peptide product was lyophilized and stored at -20°C until further use.

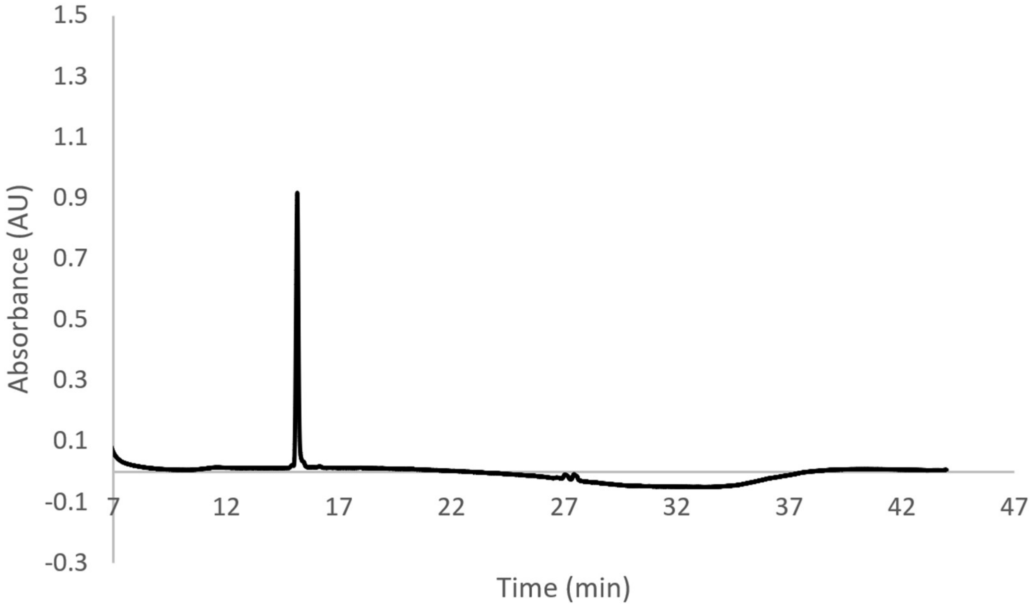

MALDI-TOF MS calculated [M]: 760.4565, found 760.603

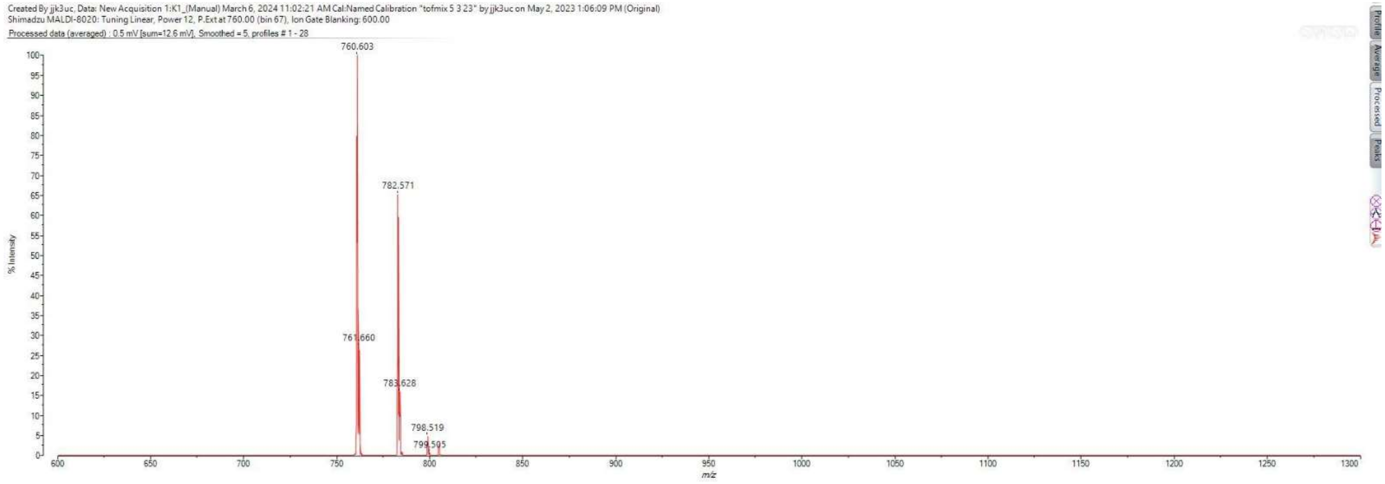

Scheme S27. Synthesis of SGIA(Kac)TAL

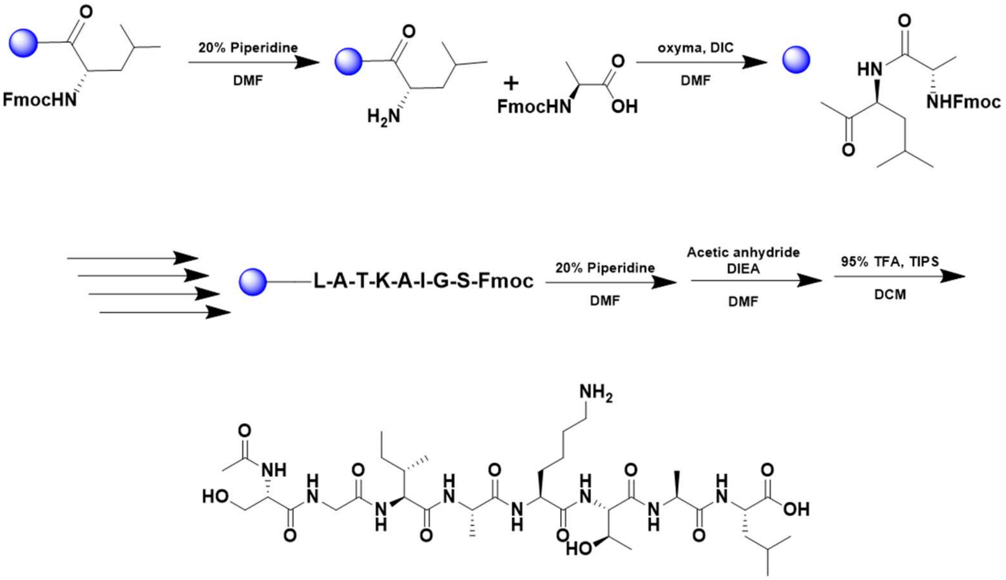

A 25 mL vessel of CEM discover bio manual peptide synthesizer was charged with 0.25 mmol of isoleucine wang resin. The Fmoc group was removed by using a 20% piperidine solution in DMF (10 mL). Using Synergy software, the deprotection protocol was run. The piperidine solution was drained and the resin was washed with DMF (4 x 10 mL). Fmoc-L-alanine (5 eq, 1.25 mM) along with Oxyma (5 eq, 1.25 mM) and DIC (5 eq, 1.35 mmol) in DMF was added to the reaction vessel and the coupling protocol was run. The amino acid solution was drained, and the resin was washed with DMF (2 x 10 mL). The fmoc removal and coupling procedure was repeated as before using the same equivalencies for the remaining amino acids. To remove the peptide from resin, a TFA cocktail solution (95% TFA, 2.5% TIPS, and 2.5% DCM) was added to the resin and agitated for 2 hours. The resin was filtered, and the resulting solution was concentrated in vacuo. The peptide was trituated with cold diethyl ether and purified using reverse phase HPLC using H_2O/CH_3CN. The sample was analyzed for purity using a Waters 1525 Binary HPLC Pump using a Phenomenex Luna 5u C8(2) 100A (250 x 4.60 mm) column; gradient eluted with H_20/CH_3CN. Molecular weight was confirmed using high resolution electrospray ionization mass spectrometry (HRMS, ESI/MS) analyses obtained on an Agilent 6545B Q-TOF LC/MS equipped with 1260 infinity II LC system with auto sampler. The final peptide product was lyophilized and stored at -20°C until further use.

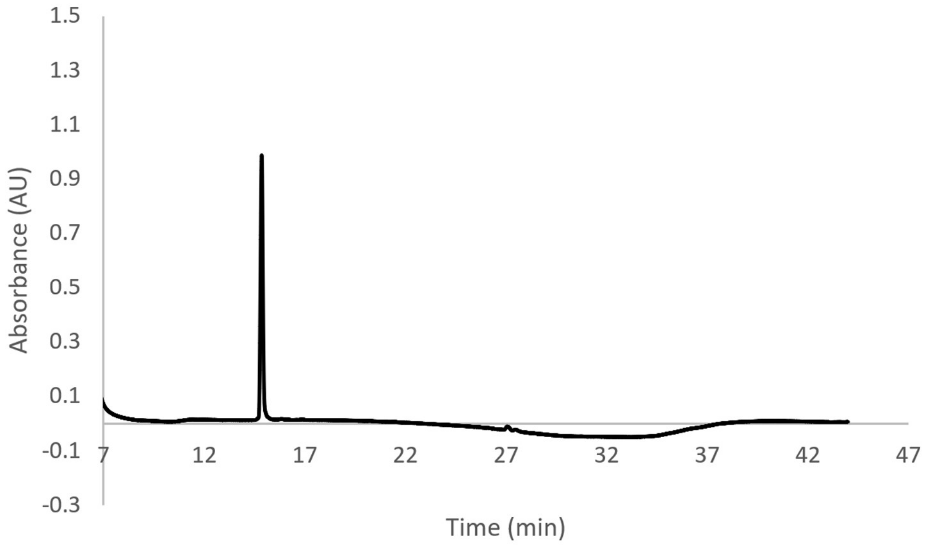

MALDI-TOF MS calculated [M]: 802.4669, found 802.204

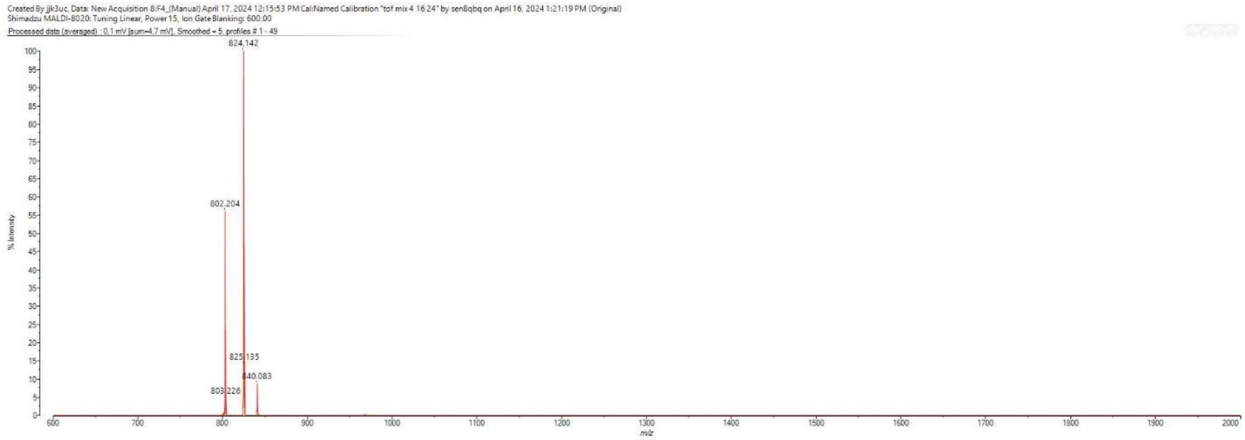

Scheme S28. Synthesis of SNPKPLVL

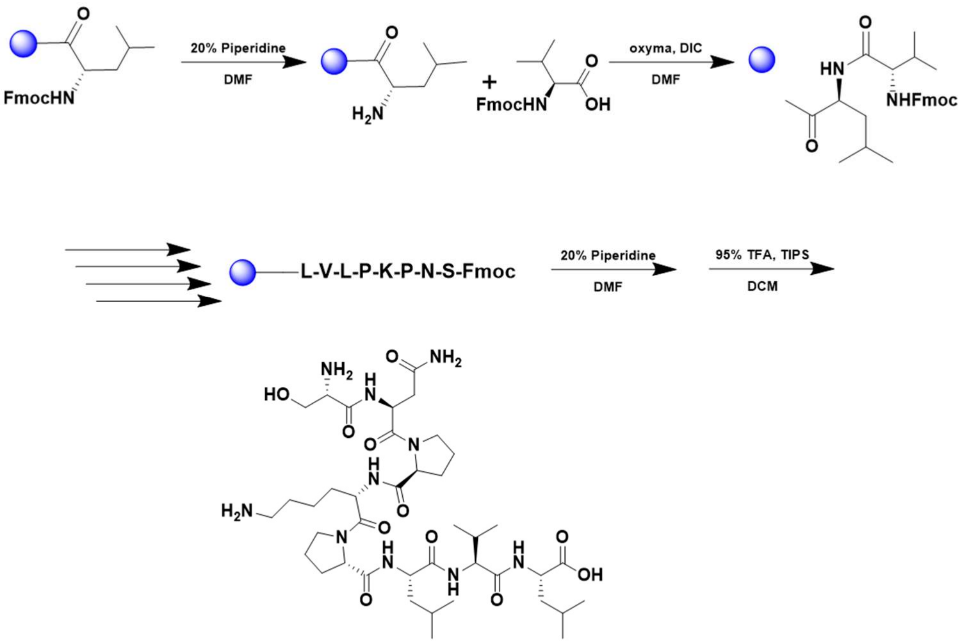

A 25 mL vessel of CEM discover bio manual peptide synthesizer was charged with 0.25 mmol of leucine wang resin. The Fmoc group was removed by using a 20% piperidine solution in DMF (10 mL). Using Synergy software, the deprotection protocol was run. The piperidine solution was drained and the resin was washed with DMF (4 x 10 mL). Fmoc-L-valine (5 eq, 1.25 mM) along with Oxyma (5 eq, 1.25 mM) and DIC (5 eq, 1.35 mmol) in DMF was added to the reaction vessel and the coupling protocol was run. The amino acid solution was drained, and the resin was washed with DMF (2 x 10 mL). The fmoc removal and coupling procedure was repeated as before using the same equivalencies for the remaining amino acids. To remove the peptide from resin, a TFA cocktail solution (95% TFA, 2.5% TIPS, and 2.5% DCM) was added to the resin and agitated for 2 hours. The resin was filtered, and the resulting solution was concentrated in vacuo. The peptide was triturated with cold diethyl ether and purified using reverse phase HPLC using H_2O/CH_3CN. The sample was analyzed for purity using a Waters 1525 Binary HPLC Pump using a Phenomenex Luna 5u C8(2) 100A (250 x 4.60 mm) column; gradient eluted with H_20/CH_3CN. Molecular weight was confirmed using high resolution electrospray ionization mass spectrometry (HRMS, ESI/MS) analyses obtained on an Agilent 6545B Q-TOF LC/MS equipped with 1260 infinity II LC system with auto sampler. The final peptide product was lyophilized and stored at -20°C until further use.

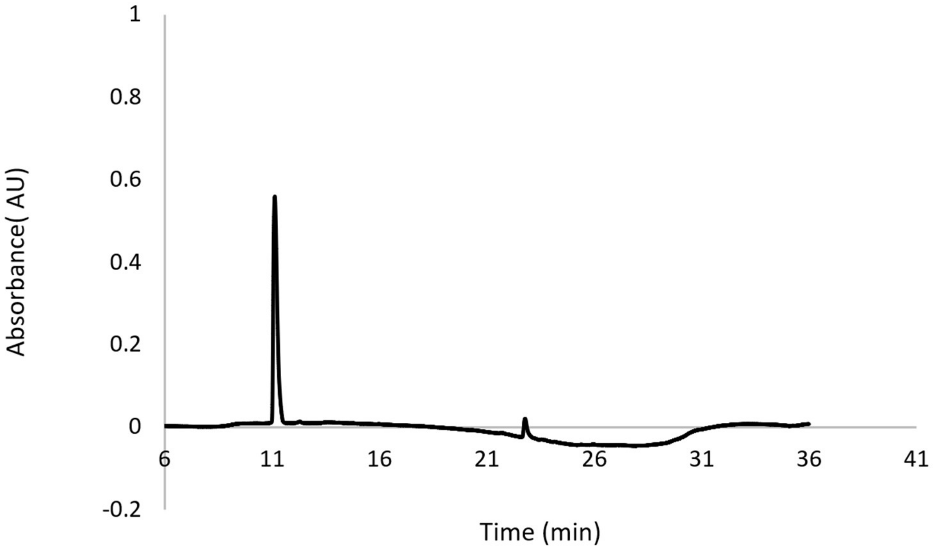

MALDI-TOF MS calculated [M]: 867.5299, found 868.453

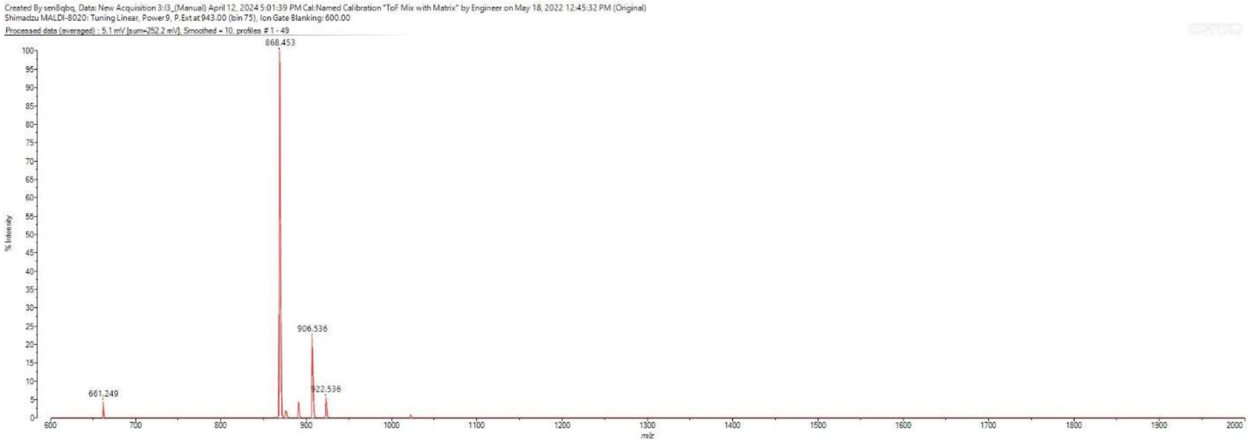

Scheme S29. Synthesis of SNP(Kac)PLVL

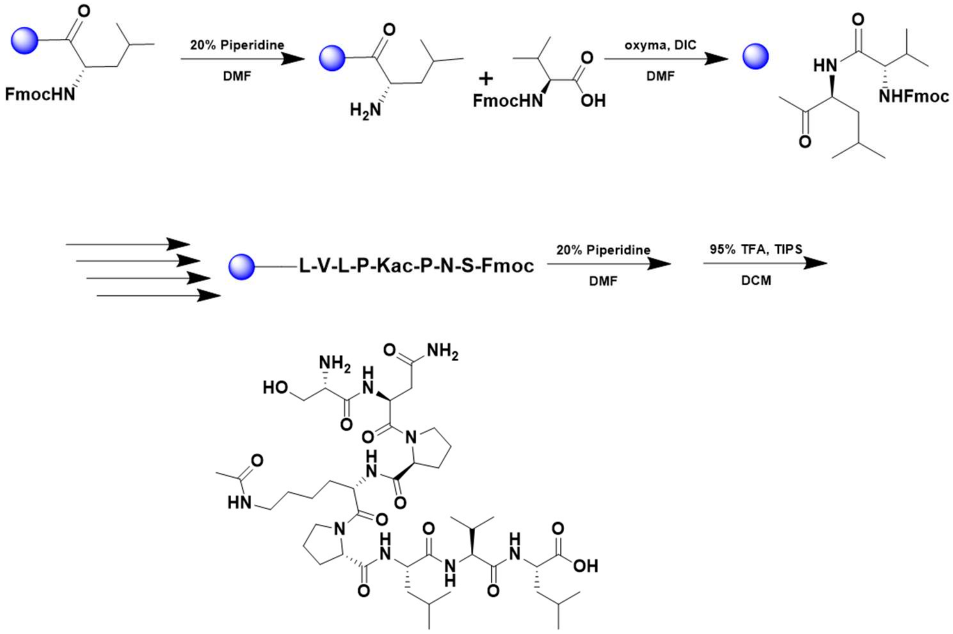

A 25 mL vessel of CEM discover bio manual peptide synthesizer was charged with 0.25 mmol of leucine wang resin. The Fmoc group was removed by using a 20% piperidine solution in DMF (10 mL). Using Synergy software, the deprotection protocol was run. The piperidine solution was drained and the resin was washed with DMF (4 x 10 mL). Fmoc-L-valine (5 eq, 1.25 mM) along with Oxyma (5 eq, 1.25 mM) and DIC (5 eq, 1.35 mmol) in DMF was added to the reaction vessel and the coupling protocol was run. The amino acid solution was drained, and the resin was washed with DMF (2 x 10 mL). The fmoc removal and coupling procedure was repeated as before using the same equivalencies for the remaining amino acids. To remove the peptide from resin, a TFA cocktail solution (95% TFA, 2.5% TIPS, and 2.5% DCM) was added to the resin and agitated for 2 hours. The resin was filtered, and the resulting solution was concentrated in vacuo. The peptide was trituated with cold diethyl ether and purified using reverse phase HPLC using H_2O/CH_3CN. The sample was analyzed for purity using a Waters 1525 Binary HPLC Pump using a Phenomenex Luna 5u C8(2) 100A (250 x 4.60 mm) column; gradient eluted with H_20/CH_3CN. Molecular weight was confirmed using high resolution electrospray ionization mass spectrometry (HRMS, ESI/MS) analyses obtained on an Agilent 6545B Q-TOF LC/MS equipped with 1260 infinity II LC system with auto sampler. The final peptide product was lyophilized and stored at -20°C until further use.

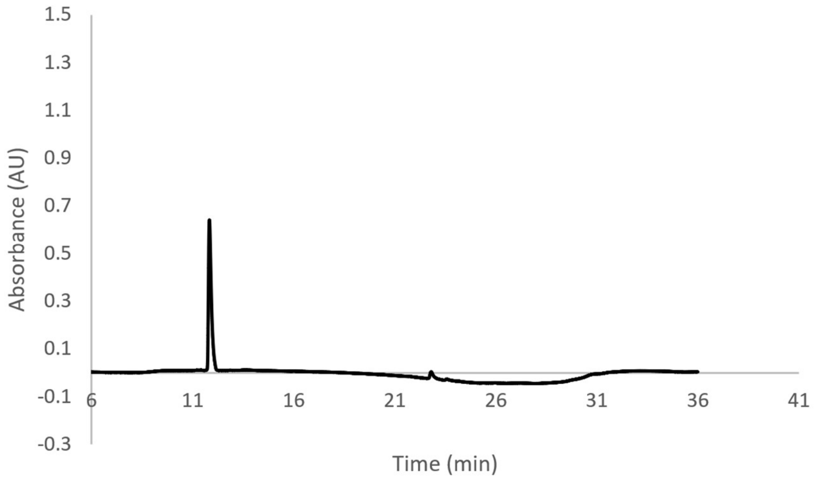

MALDI-TOF MS calculated [M]: 911.5190, found 910.136

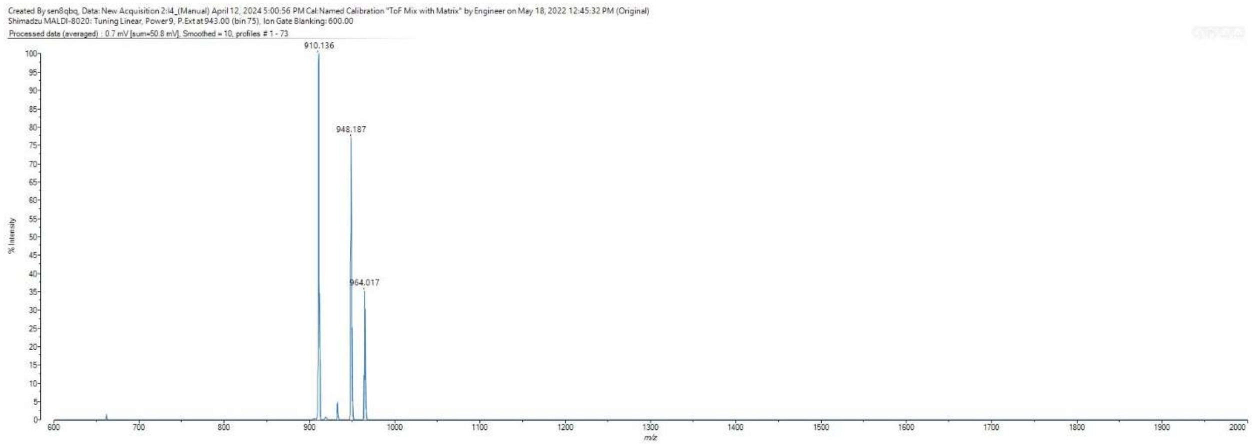

Scheme S30. Synthesis of VVYPWTQRF

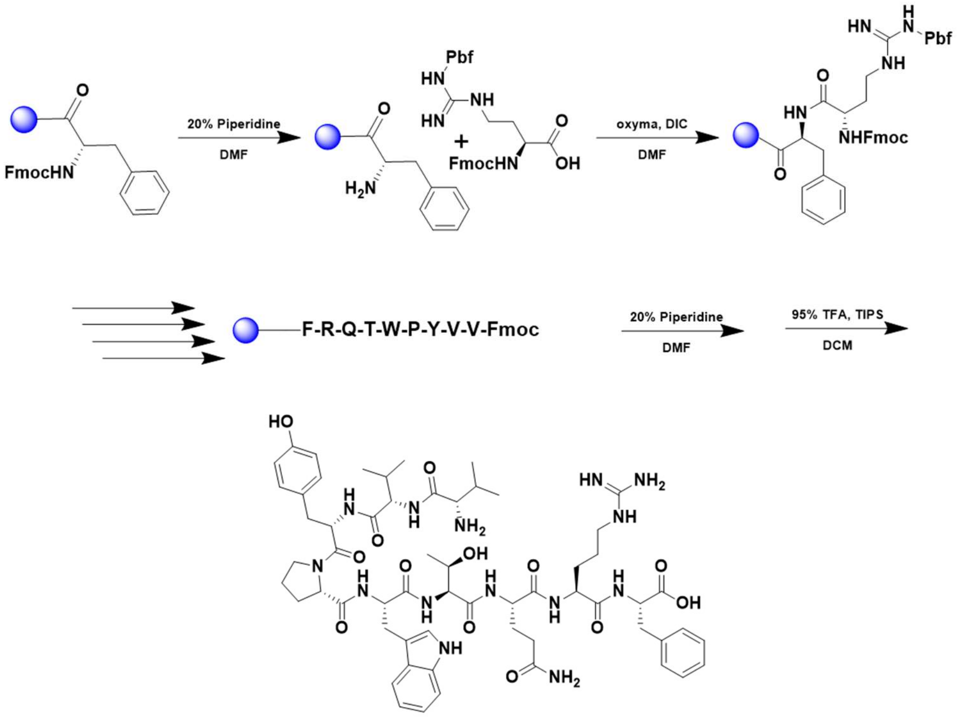

A 25 mL vessel of CEM discover bio manual peptide synthesizer was charged with 0.25 mmol of phenylalanine wang resin. The Fmoc group was removed by using a 20% piperidine solution in DMF (10 mL). Using Synergy software, the deprotection protocol was run. The piperidine solution was drained and the resin was washed with DMF (4 x 10 mL). Fmoc-L-arginine(Pbf)-OH (5 eq, 1.25 mM) along with Oxyma (5 eq, 1.25 mM) and DIC (5 eq, 1.35 mmol) in DMF was added to the reaction vessel and the coupling protocol was run. The amino acid solution was drained, and the resin was washed with DMF (2 x 10 mL). The fmoc removal and coupling procedure was repeated as before using the same equivalencies for the remaining amino acids. To remove the peptide from resin, a TFA cocktail solution (95% TFA, 2.5% TIPS, and 2.5% DCM) was added to the resin and agitated for 2 hours. The resin was filtered, and the resulting solution was concentrated in vacuo. The peptide was trituated with cold diethyl ether and purified using reverse phase HPLC using H_2O/CH_3CN. The sample was analyzed for purity using a Waters 1525 Binary HPLC Pump using a Phenomenex Luna 5u C8(2) 100A (250 x 4.60 mm) column; gradient eluted with H_20/CH_3CN. Molecular weight was confirmed using high resolution electrospray ionization mass spectrometry (HRMS, ESI/MS) analyses obtained on an Agilent 6545B Q-TOF LC/MS equipped with 1260 infinity II LC system with auto sampler. The final peptide product was lyophilized and stored at -20°C until further use.

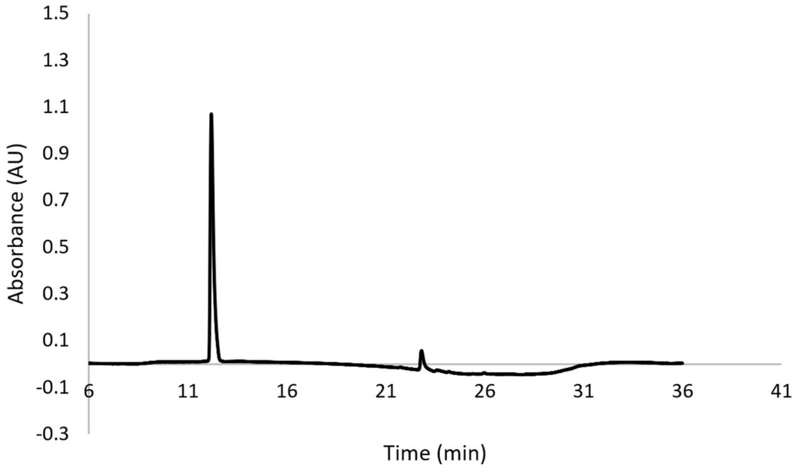

MALDI-TOF MS calculated [M]: 1195.6259, found 1195.078

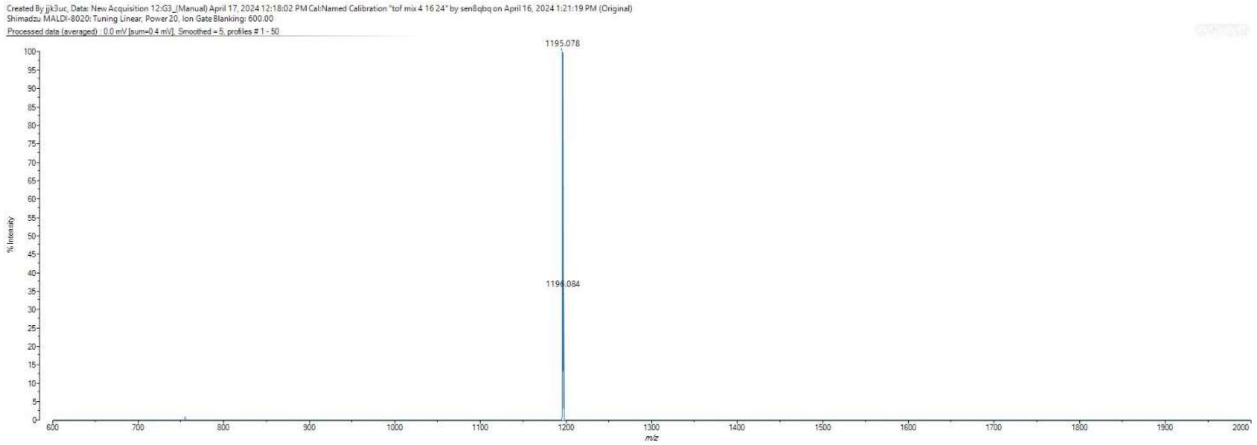

Scheme S31. Synthesis of VVYPWTQ(cit)F

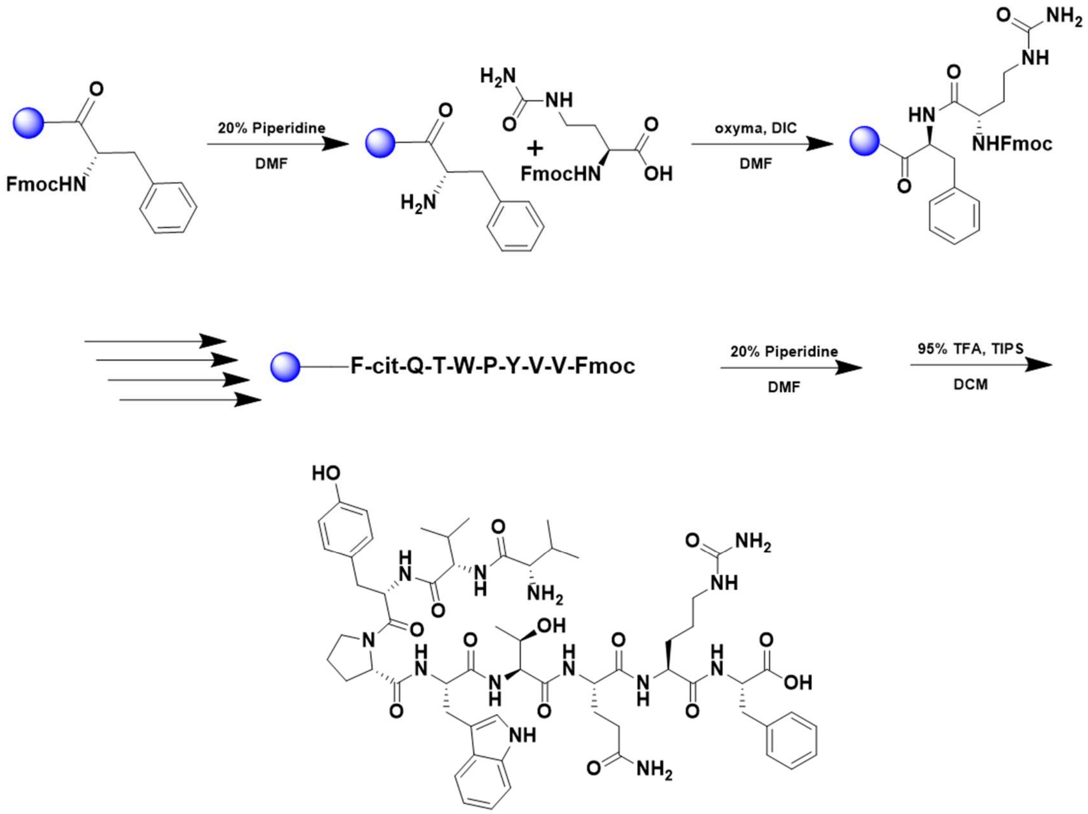

A 25 mL vessel of CEM discover bio manual peptide synthesizer was charged with 0.25 mmol of phenylalanine wang resin. The Fmoc group was removed by using a 20% piperidine solution in DMF (10 mL). Using Synergy software, the deprotection protocol was run. The piperidine solution was drained and the resin was washed with DMF (4 x 10 mL). Fmoc-L-citruline (5 eq, 1.25 mM) along with Oxyma (5 eq, 1.25 mM) and DIC (5 eq, 1.35 mmol) in DMF was added to the reaction vessel and the coupling protocol was run. The amino acid solution was drained, and the resin was washed with DMF (2 x 10 mL). The fmoc removal and coupling procedure was repeated as before using the same equivalencies for the remaining amino acids. To remove the peptide from resin, a TFA cocktail solution (95% TFA, 2.5% TIPS, and 2.5% DCM) was added to the resin and agitated for 2 hours. The resin was filtered, and the resulting solution was concentrated in vacuo. The peptide was trituated with cold diethyl ether and purified using reverse phase HPLC using H_2O/CH_3CN. The sample was analyzed for purity using a Waters 1525 Binary HPLC Pump using a Phenomenex Luna 5u C8(2) 100A (250 x 4.60 mm) column; gradient eluted with H_20/CH_3CN. Molecular weight was confirmed using high resolution electrospray ionization mass spectrometry (HRMS, ESI/MS) analyses obtained on an Agilent 6545B Q-TOF LC/MS equipped with 1260 infinity II LC system with auto sampler. The final peptide product was lyophilized and stored at -20°C until further use.

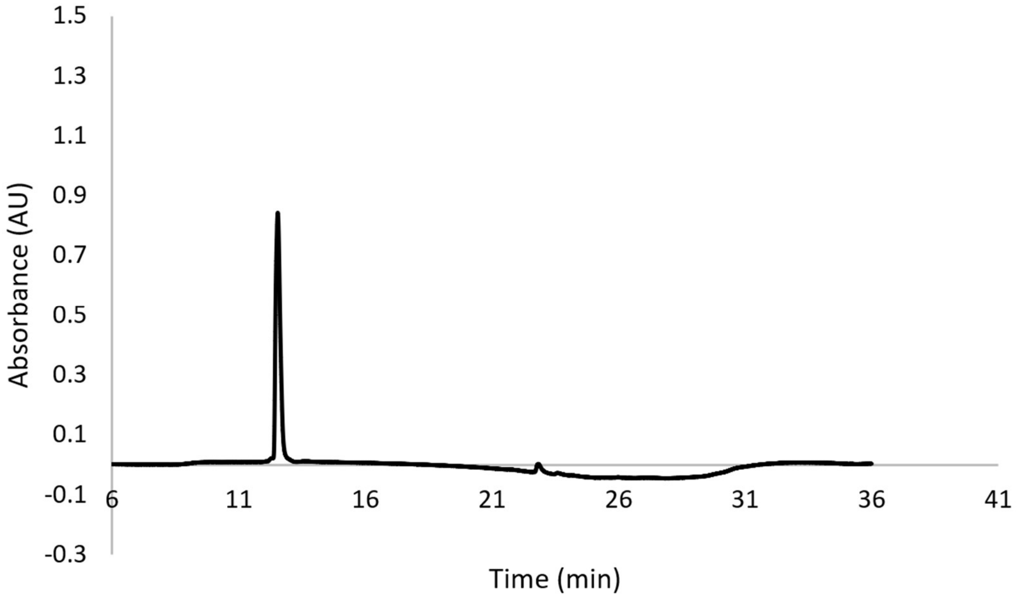

MALDI-TOF MS calculated [M]: 1196.6099, found 1195.704

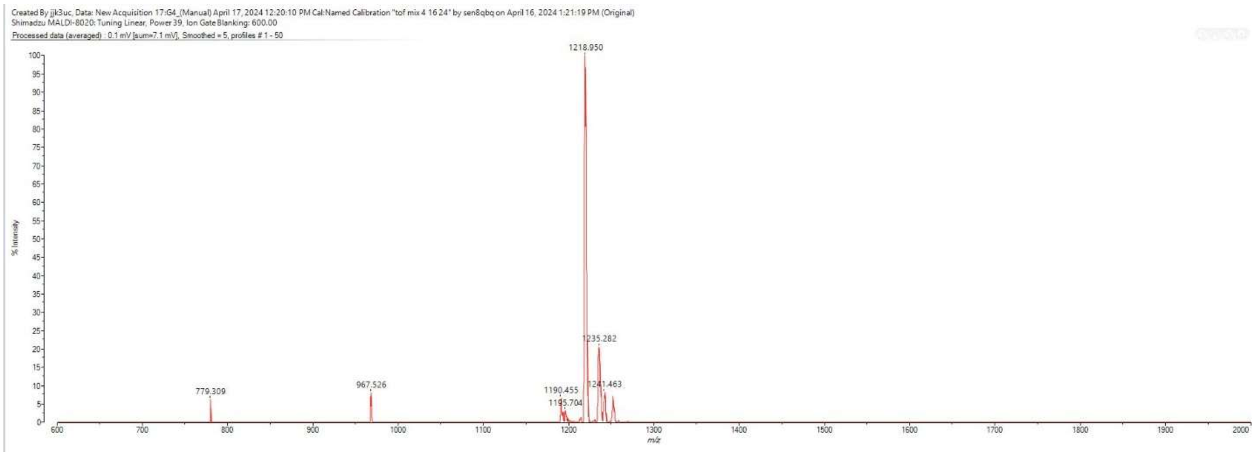

Scheme S32. Synthesis of RQYDKFLTHF

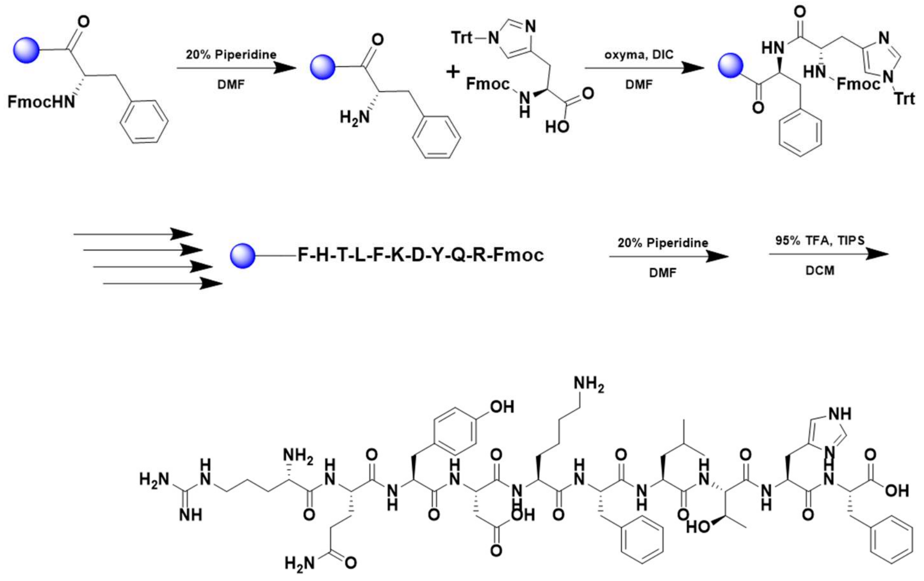

A 25 mL vessel of CEM discover bio manual peptide synthesizer was charged with 0.25 mmol of phenylalanine wang resin. The Fmoc group was removed by using a 20% piperidine solution in DMF (10 mL). Using Synergy software, the deprotection protocol was run. The piperidine solution was drained and the resin was washed with DMF (4 x 10 mL). Fmoc-L-histadine(Trt)-OH (5 eq, 1.25 mM) along with Oxyma (5 eq, 1.25 mM) and DIC (5 eq, 1.35 mmol) in DMF was added to the reaction vessel and the coupling protocol was run. The amino acid solution was drained, and the resin was washed with DMF (2 x 10 mL). The fmoc removal and coupling procedure was repeated as before using the same equivalencies for the remaining amino acids. To remove the peptide from resin, a TFA cocktail solution (95% TFA, 2.5% TIPS, and 2.5% DCM) was added to the resin and agitated for 2 hours. The resin was filtered, and the resulting solution was concentrated in vacuo. The peptide was trituated with cold diethyl ether and purified using reverse phase HPLC using H_2O/CH_3CN. The sample was analyzed for purity using a Waters 1525 Binary HPLC Pump using a Phenomenex Luna 5u C8(2) 100A (250 x 4.60 mm) column; gradient eluted with H_20/CH_3CN. Molecular weight was confirmed using high resolution electrospray ionization mass spectrometry (HRMS, ESI/MS) analyses obtained on an Agilent 6545B Q-TOF LC/MS equipped with 1260 infinity II LC system with auto sampler. The final peptide product was lyophilized and stored at -20°C until further use.

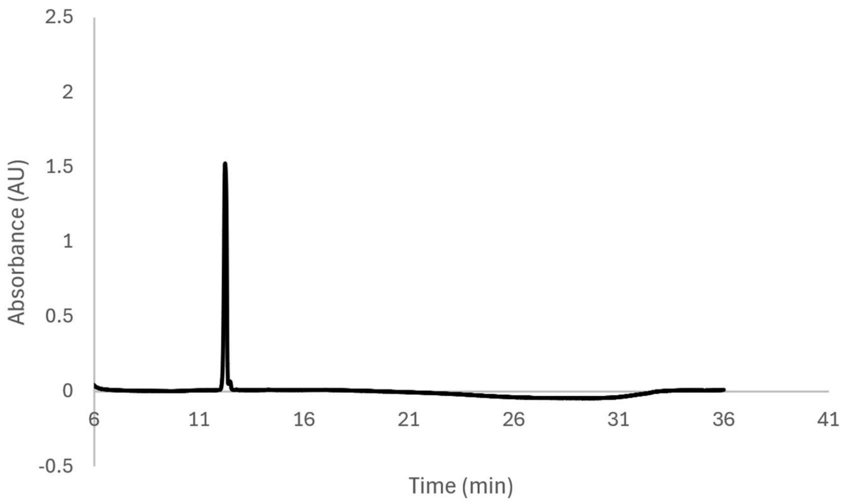

MALDI-TOF MS calculated [M]: 1354.6903, found 1354.156

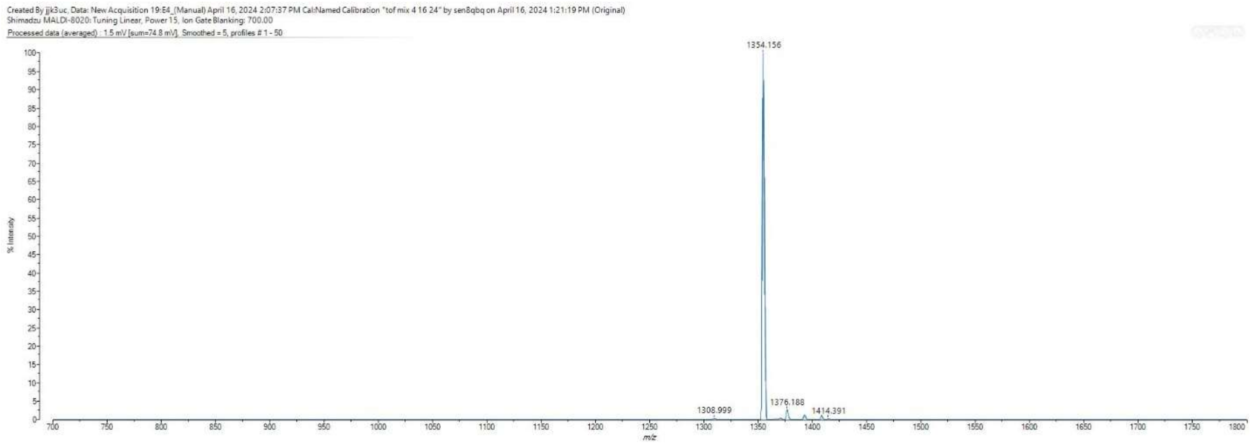

Scheme S33. Synthesis of citQYDKFLTHF

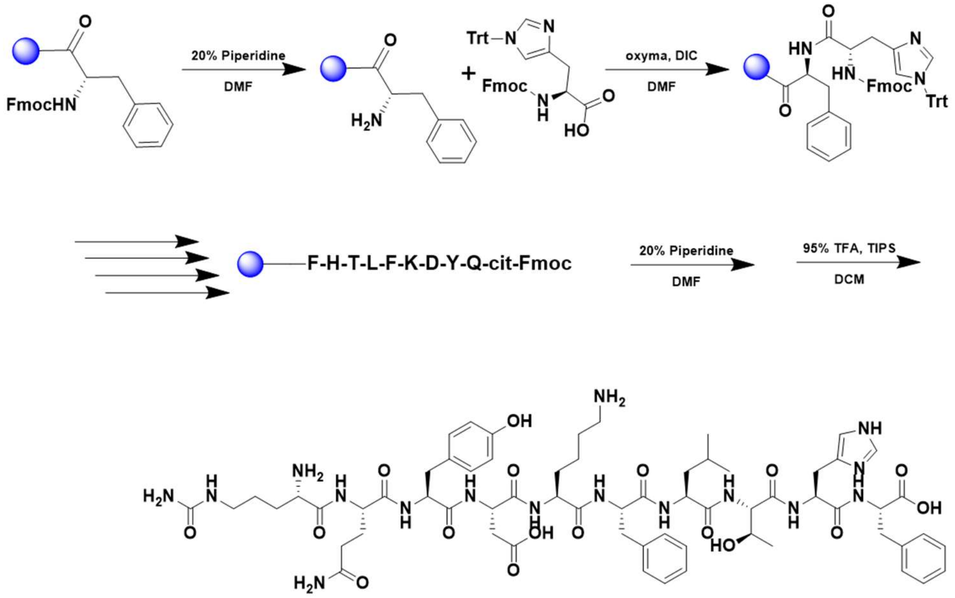

A 25 mL vessel of CEM discover bio manual peptide synthesizer was charged with 0.25 mmol of phenylalanine wang resin. The Fmoc group was removed by using a 20% piperidine solution in DMF (10 mL). Using Synergy software, the deprotection protocol was run. The piperidine solution was drained and the resin was washed with DMF (4 x 10 mL). Fmoc-L-histadine(Trt)-OH (5 eq, 1.25 mM) along with Oxyma (5 eq, 1.25 mM) and DIC (5 eq, 1.35 mmol) in DMF was added to the reaction vessel and the coupling protocol was run. The amino acid solution was drained, and the resin was washed with DMF (2 x 10 mL). The fmoc removal and coupling procedure was repeated as before using the same equivalencies for the remaining amino acids. To remove the peptide from resin, a TFA cocktail solution (95% TFA, 2.5% TIPS, and 2.5% DCM) was added to the resin and agitated for 2 hours. The resin was filtered, and the resulting solution was concentrated in vacuo. The peptide was triturated with cold diethyl ether and purified using reverse phase HPLC using H_2O/CH_3CN. The sample was analyzed for purity using a Waters 1525 Binary HPLC Pump using a Phenomenex Luna 5u C8(2) 100A (250 x 4.60 mm) column; gradient eluted with H_20/CH_3CN. Molecular weight was confirmed using high resolution electrospray ionization mass spectrometry (HRMS, ESI/MS) analyses obtained on an Agilent 6545B Q-TOF LC/MS equipped with 1260 infinity II LC system with auto sampler. The final peptide product was lyophilized and stored at -20°C until further use.

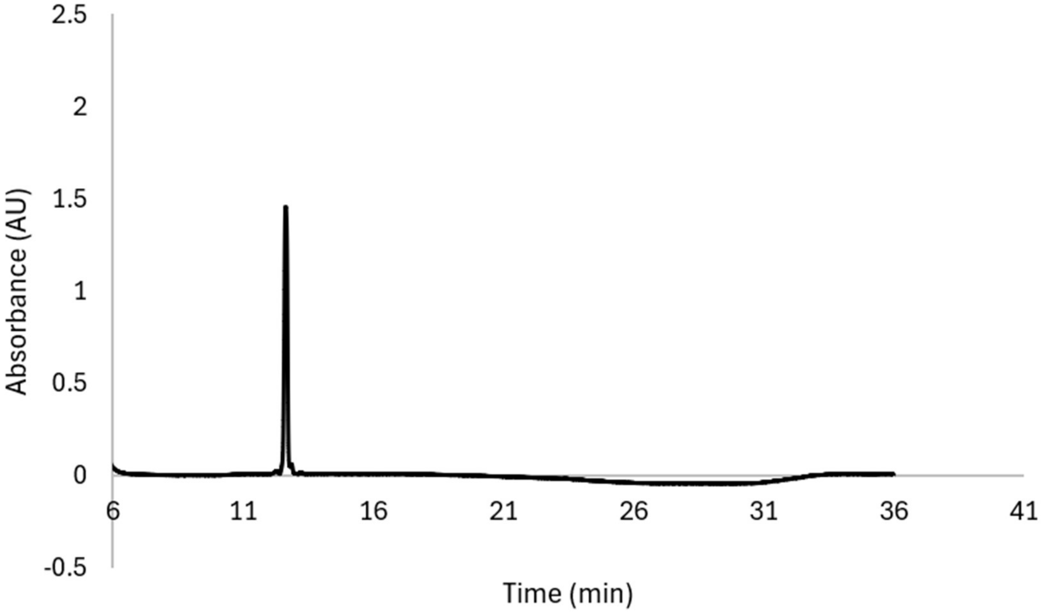

MALDI-TOF MS calculated [M]: 1355.6743, found 1355.338

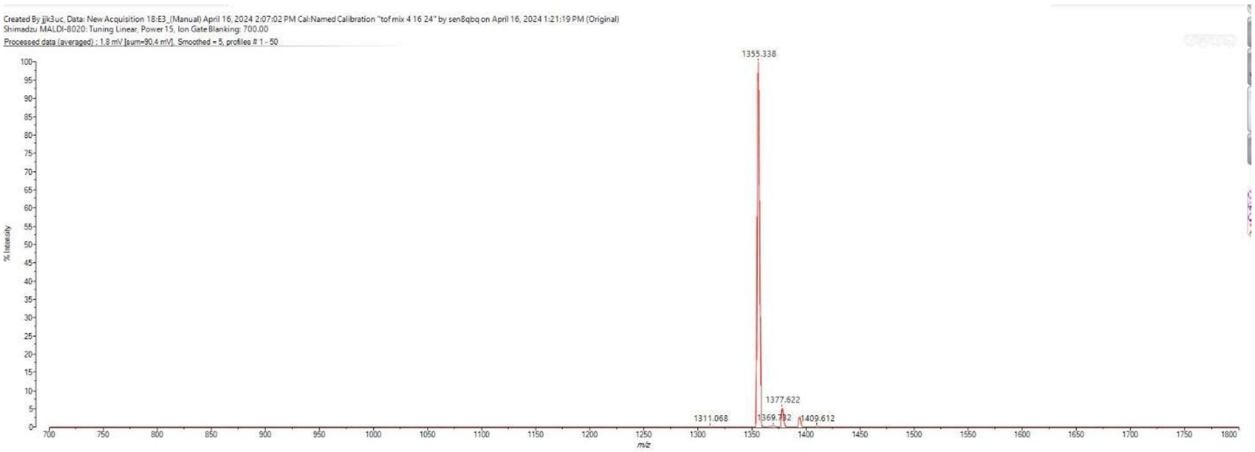

Scheme S34. Synthesis of RSPSPKTSL

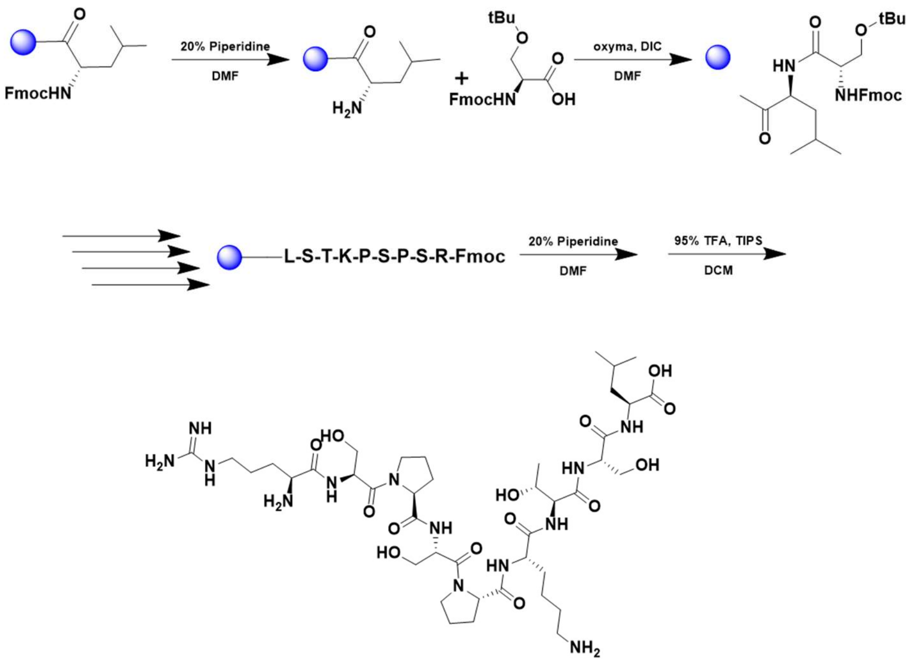

A 25 mL vessel of CEM discover bio manual peptide synthesizer was charged with 0.25 mmol of leucine wang resin. The Fmoc group was removed by using a 20% piperidine solution in DMF (10 mL). Using Synergy software, the deprotection protocol was run. The piperidine solution was drained and the resin was washed with DMF (4 x 10 mL). Fmoc-L-serine(tBu)-OH (5 eq, 1.25 mM) along with Oxyma (5 eq, 1.25 mM) and DIC (5 eq, 1.35 mmol) in DMF was added to the reaction vessel and the coupling protocol was run. The amino acid solution was drained, and the resin was washed with DMF (2 x 10 mL). The fmoc removal and coupling procedure was repeated as before using the same equivalencies for the remaining amino acids. To remove the peptide from resin, a TFA cocktail solution (95% TFA, 2.5% TIPS, and 2.5% DCM) was added to the resin and agitated for 2 hours. The resin was filtered, and the resulting solution was concentrated in vacuo. The peptide was trituated with cold diethyl ether and purified using reverse phase HPLC using H_2O/CH_3CN. The sample was analyzed for purity using a Waters 1525 Binary HPLC Pump using a Phenomenex Luna 5u C8(2) 100A (250 x 4.60 mm) column; gradient eluted with H_20/CH_3CN. Molecular weight was confirmed using high resolution electrospray ionization mass spectrometry (HRMS, ESI/MS) analyses obtained on an Agilent 6545B Q-TOF LC/MS equipped with 1260 infinity II LC system with auto sampler. The final peptide product was lyophilized and stored at -20°C until further use.

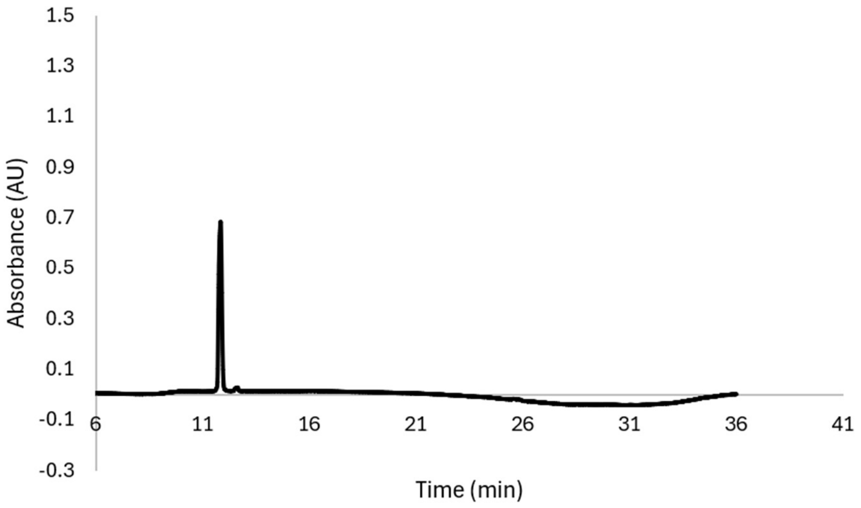

MALDI-TOF MS calculated [M]: 972.5473, found 972.855

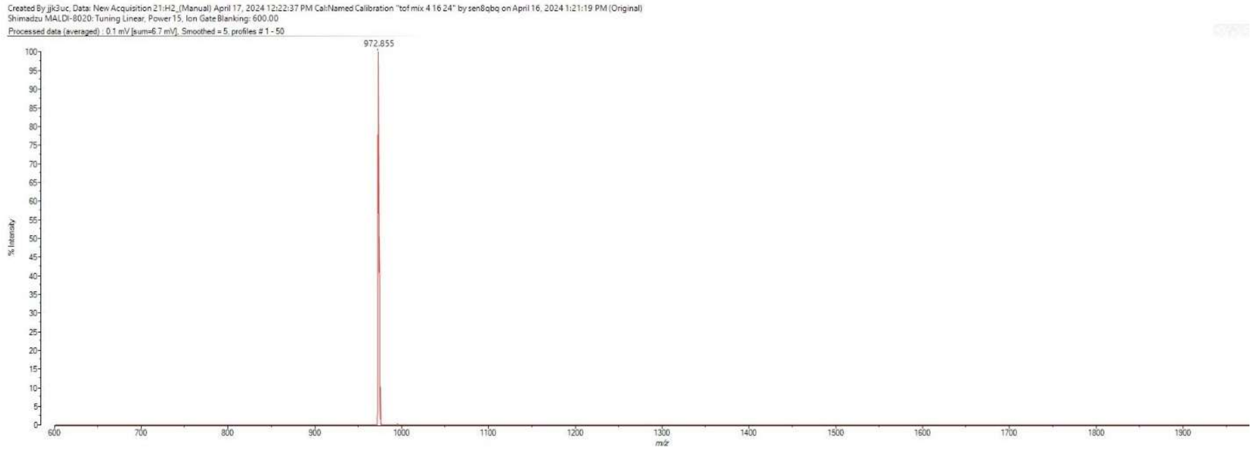

Scheme S35. Synthesis of RSP(SPO4)PKTSL

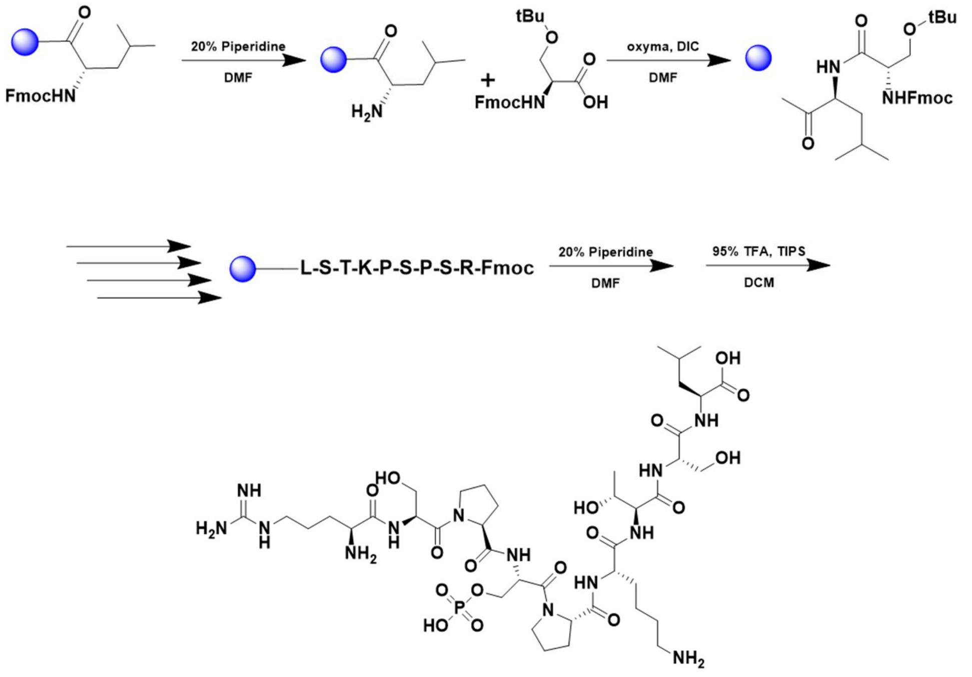

A 25 mL vessel of CEM discover bio manual peptide synthesizer was charged with 0.25 mmol of leucine wang resin. The Fmoc group was removed by using a 20% piperidine solution in DMF (10 mL). Using Synergy software, the deprotection protocol was run. The piperidine solution was drained and the resin was washed with DMF (4 x 10 mL). Fmoc-L-serine(tBu)-OH (5 eq, 1.25 mM) along with Oxyma (5 eq, 1.25 mM) and DIC (5 eq, 1.35 mmol) in DMF was added to the reaction vessel and the coupling protocol was run. The amino acid solution was drained, and the resin was washed with DMF (2 x 10 mL). The fmoc removal and coupling procedure was repeated as before using the same equivalencies for the remaining amino acids. To remove the peptide from resin, a TFA cocktail solution (95% TFA, 2.5% TIPS, and 2.5% DCM) was added to the resin and agitated for 2 hours. The resin was filtered, and the resulting solution was concentrated in vacuo. The peptide was triturated with cold diethyl ether and purified using reverse phase HPLC using H_2O/CH_3CN. The sample was analyzed for purity using a Waters 1525 Binary HPLC Pump using a Phenomenex Luna 5u C8(2) 100A (250 x 4.60 mm) column; gradient eluted with H_20/CH_3CN. Molecular weight was confirmed using high resolution electrospray ionization mass spectrometry (HRMS, ESI/MS) analyses obtained on an Agilent 6545B Q-TOF LC/MS equipped with 1260 infinity II LC system with auto sampler. The final peptide product was lyophilized and stored at -20^oC until further use.

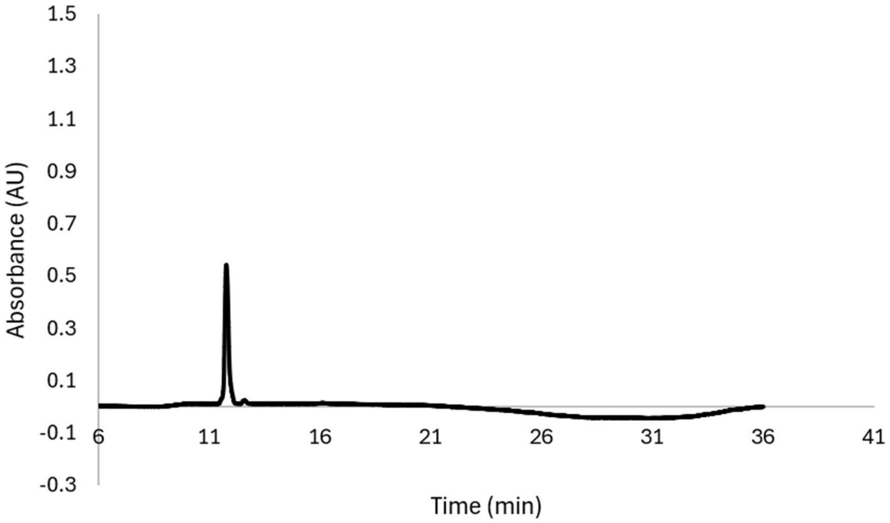

MALDI-TOF MS calculated [M]: 1051.5058, found 1052.519

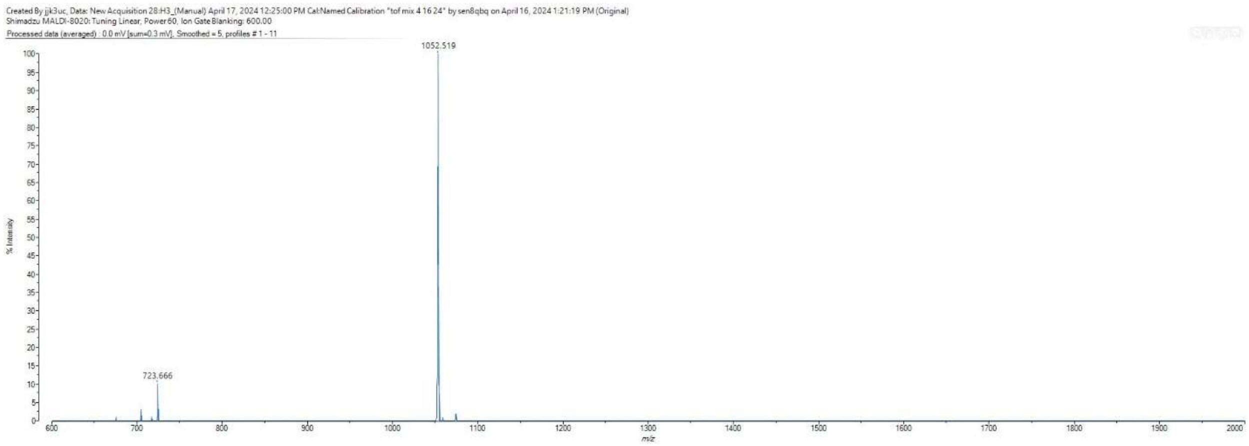

Supplement: Supplementary file 1 — cb4c00312_si_001.pdf [file cb4c00312_si_001.pdf]
